# Histone methyltransferase Ezh2 coordinates mammalian axon regeneration via regulation of key regenerative pathways

**DOI:** 10.1172/JCI163145

**Published:** 2024-02-01

**Authors:** Xue-Wei Wang, Shu-Guang Yang, Ming-Wen Hu, Rui-Ying Wang, Chi Zhang, Anish R. Kosanam, Arinze J. Ochuba, Jing-Jing Jiang, Ximei Luo, Yun Guan, Jiang Qian, Chang-Mei Liu, Feng-Quan Zhou

**Affiliations:** 1 Department of Orthopaedic Surgery, Johns Hopkins University School of Medicine, Baltimore, Maryland, USA.; 2 Department of Molecular Medicine, University of South Florida Morsani College of Medicine, Tampa, Florida, USA.; 3 Department of Ophthalmology and; 4 Department of Anesthesiology and Critical Care Medicine, Johns Hopkins University School of Medicine, Baltimore, Maryland, USA.; 5 State Key Laboratory of Stem Cell and Reproductive Biology, Institute of Zoology, Chinese Academy of Sciences, Beijing, China.; 6 The Solomon H. Snyder Department of Neuroscience, Johns Hopkins University School of Medicine, Baltimore, Maryland, USA.

**Keywords:** Neuroscience, Ophthalmology, Epigenetics, Neurodegeneration, Neurodevelopment

## Abstract

Current treatments for neurodegenerative diseases and neural injuries face major challenges, primarily due to the diminished regenerative capacity of neurons in the mammalian CNS as they mature. Here, we investigated the role of Ezh2, a histone methyltransferase, in regulating mammalian axon regeneration. We found that Ezh2 declined in the mouse nervous system during maturation but was upregulated in adult dorsal root ganglion neurons following peripheral nerve injury to facilitate spontaneous axon regeneration. In addition, overexpression of *Ezh2* in retinal ganglion cells in the CNS promoted optic nerve regeneration via both histone methylation–dependent and –independent mechanisms. Further investigation revealed that Ezh2 fostered axon regeneration by orchestrating the transcriptional silencing of genes governing synaptic function and those inhibiting axon regeneration, while concurrently activating various factors that support axon regeneration. Notably, we demonstrated that GABA transporter 2, encoded by *Slc6a13,* acted downstream of Ezh2 to control axon regeneration. Overall, our study underscores the potential of modulating chromatin accessibility as a promising strategy for promoting CNS axon regeneration.

## Abstract 2

<p>Ezh2 fosters optic nerve regeneration by regulating multiple categories of target genes in retinal ganglion cells.</p>

## Introduction

Axon degeneration and neuronal cell death are common consequences of neurodegenerative diseases and neural injuries. Unfortunately, current clinical therapeutics for neural injuries and neurodegenerative diseases still fall short of success. In the mammalian CNS, the inability of mature neurons to regenerate axons after injury or neurodegeneration results in poor functional recovery and permanent disabilities. Therefore, understanding why mature neurons in the mammalian CNS cannot regrow axons has been a longstanding challenge for the field. Research over the past decades has revealed that the low intrinsic axon growth competency of mature CNS neurons (1–3), together with extrinsic inhibitory molecules (4–6), are the major contributors to unsatisfactory regenerative outcomes. During development, young neurons are intrinsically competent in axon growth to establish neural circuits, whereas adult neurons possess poor axon growth ability to maintain circuit stability. Moreover, the inhibitory extracellular environment also limits unnecessary axon sprouting, acting as another factor to stabilize the neural circuits (7).

During maturation, the cellular state of neurons changes from favoring to limiting axon growth, likely regulated by modifications of the epigenomic and subsequent transcriptomic landscapes in neurons. Unlike CNS neurons, the axon regeneration ability of neurons in the peripheral nervous system (PNS) can be reactivated upon peripheral nerve injury by initiating a transcription-dependent regenerative response (8, 9). Recent studies demonstrated that such a response also involves massive changes in the epigenome and transcriptome of PNS neurons (10–12). It has been revealed that nerve injuries induce a common developmental-like transcriptional program in sensory neurons (12–14). Similar reversal to an embryonic transcriptomic state also occurs in mature corticospinal neurons at an early stage following spinal cord injury, although it cannot be sustained (15). Thus, it is vital to unveil the epigenomic changes that occur during neuronal maturation and PNS axon regeneration. The knowledge gained may be useful for epigenetically remodeling the transcriptomic landscape of mature CNS neurons and enhancing their axon regeneration ability.

In this study, we investigated the role of enhancer of zeste homolog 2 (Ezh2) histone methyltransferase in mammalian axon regeneration. Ezh2 is the catalytic core of the polycomb repressive complex 2 (PRC2), which catalyzes trimethylation of lysine 27 on histone H3 (H3K27me3). H3K27me3 condenses nearby chromatin to downregulate transcription (16). Although most studies focus on the role of Ezh2 as a histone methyltransferase, a number of studies clearly showed that Ezh2 can also methylate nonhistone substrates (17, 18) or exert methylation-independent functions (19–21), suggesting its versatility.

Here, we showed that *Ezh2* loss-of-function impaired spontaneous axon regeneration of dorsal root ganglion (DRG) neurons. In addition, overexpression of *Ezh2* in retinal ganglion cells (RGCs) promoted optic nerve regeneration in both histone methylation-dependent and -independent manners. Mechanistic exploration revealed that Ezh2 orchestrated mammalian axon regeneration by targeting both the intrinsic regenerative ability and the extrinsic hostile environment.

## Results

### Ezh2 is developmentally downregulated in the nervous system and upregulated in adult DRG neurons following peripheral nerve injury.

**Figure 1:**
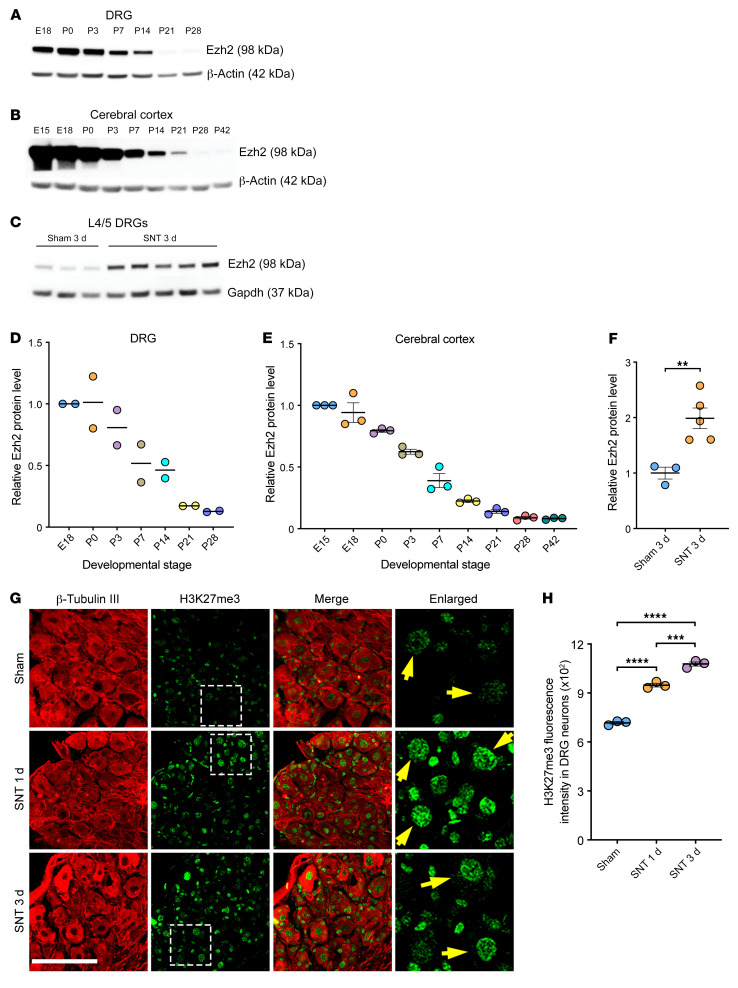
Ezh2 is developmentally downregulated in the nervous system and upregulated in DRG neurons following peripheral nerve injury. (**A** and **B**) Representative immunoblotting showing that Ezh2 is developmentally downregulated in the DRG (**A**) and cerebral cortex (**B**). (**C**) Immunoblotting showing that Ezh2 is significantly increased in L4/5 DRGs 3 days after sciatic nerve transection. (**D**) Quantification of relative protein levels of Ezh2 in **A** (*n* = 2 independent experiments). (**E**) Quantification of relative protein levels of Ezh2 in **B** (1-way ANOVA followed by Tukey’s multiple comparisons; *P* 0.0001; *n* = 3 independent experiments). (**F**) Quantification of relative protein levels of Ezh2 in **C** (unpaired, 2-tailed *t* test; *P* = 0.0092; *n* = 3 for sham, *n* = 5 for sciatic nerve transection). (**G**) Representative immunofluorescence of DRG sections showing increased H3K27me3 levels in nuclei of DRG neurons 1 or 3 days after sciatic nerve transection. DRG sections were stained with anti-H3K27me3 (green) and anti-β-tubulin III (red). The rightmost column displays enlarged images of the areas outlined in white, dashed boxes. Yellow arrows indicate H3K27me3 in nuclei of DRG neurons. Scale bars: 100 μm, 30 μm for enlarged images. (**H**) Quantification of fluorescence intensity of H3K27me3 immunoreactivity in DRG neurons in **G** (1-way ANOVA followed by Tukey’s multiple comparisons; *P* 0.0001; *n* = 3 mice for all). SNT, sciatic nerve transection. **P 0.01, ***P 0.001, ****P 0.0001.

To evaluate how Ezh2 expression was regulated during neural development, we first examined Ezh2 protein levels in the mouse DRG and cerebral cortex at different developmental stages. We found that Ezh2 was abundantly expressed in DRGs and the cortex at the late embryonic stage, remained high during the first several postnatal days, and then gradually declined to become hardly detectable at 3 to 4 weeks after birth (Figure 1, A, B, D, and E). DRG neurons extend a single axon that bifurcates into 2 branches, a peripheral branch that readily regenerates upon injury in a transcription-dependent manner (8), and a central branch lacking the spontaneous regenerative ability. Sensory axons in the mouse sciatic nerve are primarily comprised of peripheral branches of lumbar 4 and 5 (L4/5) DRG neurons. A sharp increase of Ezh2 in L4/5 DRGs was detected 3 days after sciatic nerve transection (Figure 1, C and F). Other PRC2 subunits were not discernibly altered (Supplemental Figure 1, A–E; supplemental material available online with this article; https://doi.org/10.1172/JCI163145DS1). Immunofluorescence of DRG sections showed significantly increased neuronal H3K27me3 following the injury (Figure 1, G and H), suggesting cell-autonomous upregulation of Ezh2 in DRG neurons. These results were consistent with a previous study showing increased Ezh2 and H3K27me3 in the DRG after spinal nerve ligation (22), which also injures peripheral axons of DRG neurons. During development, neurons lose axon growth capacities after reaching their targets, correlating with the decline of Ezh2. On the other hand, the upregulation of Ezh2 in DRG neurons after peripheral nerve injury accompanies the robust regenerative response, suggesting that Ezh2 might facilitate axon regeneration.

### Upregulation of Ezh2 contributes to spontaneous axon regeneration of DRG neurons in vitro and in vivo.

To test our hypothesis, we first investigated if *Ezh2* loss-of-function would impair regenerative axon growth of cultured DRG neurons. Using in vitro electroporation (23), siRNAs targeting *Ezh2* mRNA (si*Ezh2*) were transfected into DRG neurons. Control neurons were electroporated with nontargeting siRNAs (siNT). Immunoblotting confirmed that *Ezh2* was efficiently knocked down 3 days after the electroporation (Supplemental Figure 1, F and H). Thus, on the fourth day, we replated the neurons and cultured them for another 24 hours, as described in our earlier study (9). The results showed that *Ezh2* knockdown significantly reduced regenerative axon growth by approximately 25% (Supplemental Figure 1, J and L). To rule out the possibility that the phenotype was caused by off-target effects of the siRNAs, we crossed *Ezh2^fl/fl^* mice with *Advillin-Cre* mice (24) to generate *Advillin-Cre;Ezh2^fl/fl^* mice, in which *Ezh2* was specifically deleted in sensory neurons. We performed a peripheral nerve conditioning lesion in *Advillin-Cre;Ezh2^fl/fl^* and *Ezh2^fl/fl^* (control) mice and waited for 3 days, and then cultured L4/5 DRG neurons for 24 hours. Successful knockout of *Ezh2* was confirmed by immunoblotting of protein extracted from the cultured cells (Supplemental Figure 1, G and I). The remaining Ezh2 signal likely came from nonneuronal cells in the culture. We found that *Ezh2* deletion significantly impaired regenerative axon growth of conditioning lesioned DRG neurons by approximately 20% (Supplemental Figure 1, K and M). These results demonstrated that Ezh2 supported regenerative axon growth of sensory neurons in vitro.

**Figure 2:**
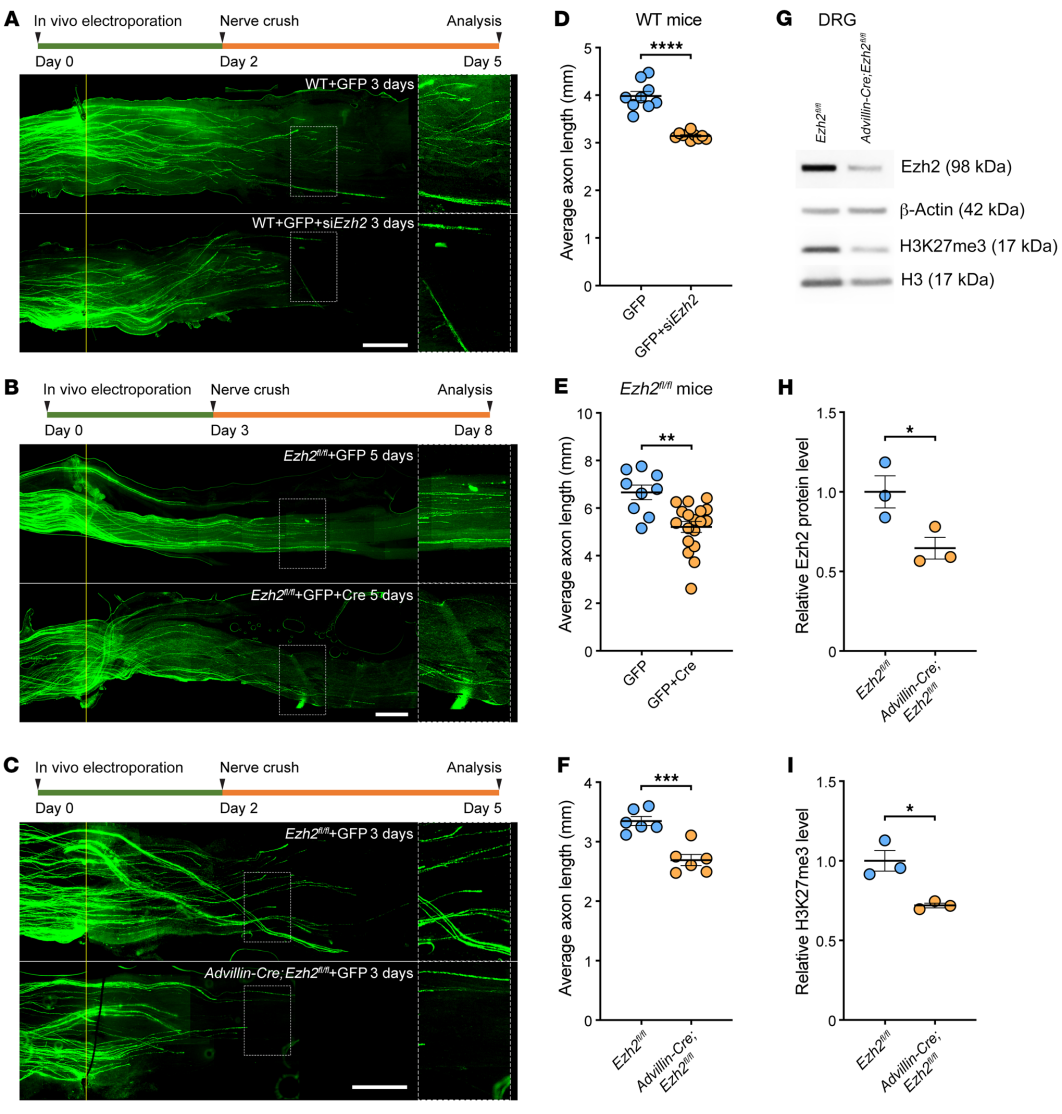
Upregulation of Ezh2 contributes to spontaneous axon regeneration of DRG neurons in vivo. (**A**–**C**) Top: Experimental timeline. Bottom: Representative images of sciatic nerves showing that *Ezh2* knockdown (**A**) or knockout (**B**) in L4/5 DRGs or sensory neuron-specific knockout of *Ezh2* (**C**) impairs spontaneous axon regeneration of DRG neurons in vivo. The right column displays enlarged images of the areas in white, dashed boxes on the left. The crush sites are aligned with the yellow line. Scale bars: 1 mm, 0.5 mm for enlarged images. (**D**) Quantification of lengths of regenerating axons in **A** (unpaired, 2-tailed *t* test; *P* 0.0001; *n* = 9 mice for control, *n* = 10 mice for *Ezh2* knockdown). (**E**) Quantification of lengths of regenerating axons in **B** (unpaired 2-tailed *t* test; *P* = 0.0011; *n* = 9 mice for control, *n* = 18 mice for *Ezh2* knockout). (**F**) Quantification of lengths of regenerating axons in **C** (unpaired 2-tailed *t* test; *P* = 0.0003; *n* = 6 mice for both). (**G**) Representative immunoblotting showing successful knockout of *Ezh2* and downregulation of H3K27me3 in DRG neurons of *Advillin-Cre;Ezh2^fl/fl^* mice. (**H**) Quantification of relative protein levels of Ezh2 in **G** (unpaired 2-tailed *t* test *P* = 0.0436; *n* = 3 independent experiments). (**I**) Quantification of relative levels of H3K27me3 in **G** (unpaired 2-tailed *t* test; *P* = 0.0137; *n* = 3 independent experiments). si*Ezh2*, siRNAs targeting *Ezh2* mRNA. *P 0.05, **P 0.01, ***P 0.001, ****P 0.0001.

To further explore if Ezh2 was also required for axon regeneration of DRG neurons in vivo, we knocked down *Ezh2* in L4/5 DRGs by in vivo electroporation of si*Ezh2*, a technique widely used in our previous studies (25, 26). *CMV-GFP* plasmid was simultaneously electroporated to label the axons. Control mice were electroporated solely with the *CMV-GFP* plasmid, as our previous study demonstrated that electroporation of siNT had no impact on axon regeneration of sensory neurons (26). Two days after the electroporation, the sciatic nerve was crushed. After 3 days, we found that *Ezh2* knockdown in DRGs significantly impaired axon regeneration of sensory neurons in vivo by approximately 20% (Figure 2, A and D). To rule out off-target effects of the siRNAs, we electroporated *CMV-Cre* and *CMV-GFP* plasmids into L4/5 DRGs of *Ezh2^fl/fl^* mice to knockout *Ezh2*. *Ezh2^fl/fl^* mice electroporated with the *CMV-GFP* plasmid only were the control group. To allow sufficient time for Cre-mediated recombination, the sciatic nerve was crushed 3 days later. Five days after the crush, we found that axon regeneration was significantly impaired by *Ezh2* knockout (Figure 2, B and E). To further rule out the possibility that the observed phenotype resulted from *Ezh2* loss-of-function in nonneuronal cells in the DRG, we electroporated the *CMV-GFP* plasmid in *Advillin-Cre;Ezh2^fl/fl^* and *Ezh2^fl/fl^* (control) mice; 2 days later, we crushed the sciatic nerve. Three days after the crush, we found that specific deletion of *Ezh2* in sensory neurons significantly reduced axon regeneration in vivo by approximately 20% (Figure 2, C and F). Successful knockout of *Ezh2* and decrease of H3K27me3 in DRG neurons of *Advillin-Cre;Ezh2^fl/fl^* mice were confirmed by immunoblotting (Figure 2, G–I). Collectively, these results demonstrated that Ezh2 upregulation contributed to spontaneous axon regeneration of DRG neurons triggered by peripheral nerve injury both in vitro and in vivo.

### Ezh2 overexpression enhances optic nerve regeneration via both histone methylation–dependent and –independent mechanisms.

**Figure 3:**
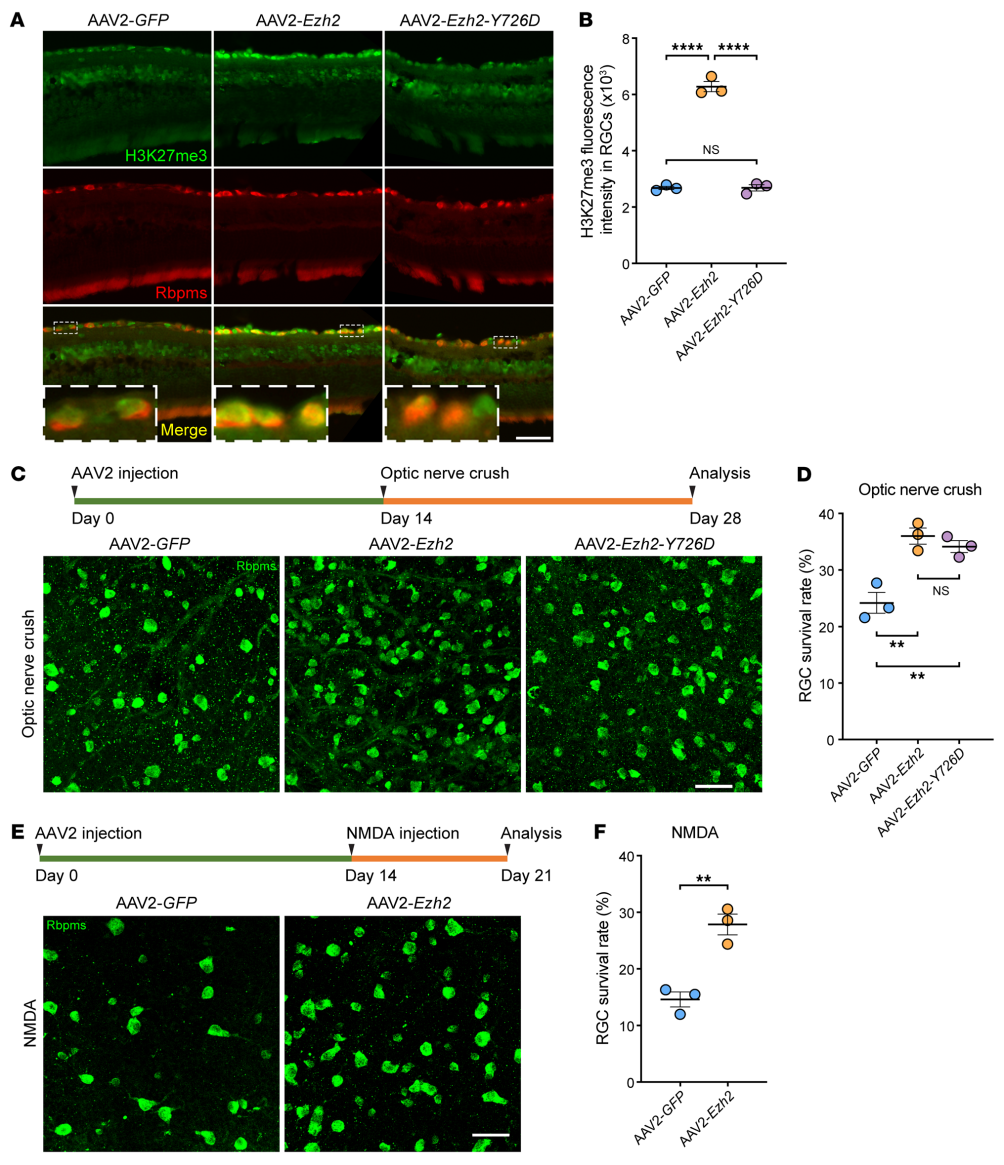
*Ezh2* overexpression enhances RGC survival after ONC or excitotoxic injury. (**A**) Representative immunofluorescence of retinal sections showing increased H3K27me3 levels in RGCs 2 weeks after intravitreal injection of AAV2-*Ezh2*, but not AAV2-*Ezh2-Y726D*. Retinal sections were stained with anti-H3K27me3 (green) and anti-Rbpms (red). Insets display enlarged images of RGCs in white, dashed boxes. Scale bars: 50 μm, 10 μm for enlarged images. (**B**) Quantification of fluorescence intensity of H3K27me3 immunoreactivity in RGCs in **A** (1-way ANOVA followed by Tukey’s multiple comparisons; *P* 0.0001; *n* = 3 mice for all; at least 150 RGCs from 10–12 nonadjacent sections were analyzed for each mouse). (**C**) Top: Experimental timeline. Bottom: Representative immunofluorescence of whole-mount retinas showing that overexpression of *Ezh2* or *Ezh2-Y726D* improves RGC survival 2 weeks after optic nerve crush. Whole-mount retinas were stained with anti-Rbpms (green). Scale bar: 50 μm. (**D**) Quantification of RGC survival rate 2 weeks after ONC in **C** (1-way ANOVA followed by Tukey’s multiple comparisons; *P* = 0.0025; *n* = 3 mice for all; 6–9 fields were analyzed for each mouse). (**E**) Top: Experimental timeline. Bottom: Representative immunofluorescence of whole-mount retinas showing that overexpression of *Ezh2* improves RGC survival 1 week after NMDA-induced excitotoxic injury. Whole-mount retinas were stained with anti-Rbpms (green). Scale bar: 50 μm. (**F**) Quantification of RGC survival rate 1 week after NMDA-induced excitotoxic injury in **E** (unpaired 2-tailed *t* test; *P* = 0.0042; *n* = 3 mice for both; 6–8 fields were analyzed for each mouse). **P 0.01, ****P 0.0001.

Since upregulation of Ezh2 contributed to axon regeneration of regenerative DRG neurons, we questioned whether forced overexpression of *Ezh2* would similarly promote axon regeneration in nonregenerative adult RGCs. We first examined if Ezh2 and other PRC2 subunits in RGCs were changed by optic nerve crush (ONC). By analyzing a single-cell RNA-Seq data set of RGCs (27), we found that their mRNA levels remained relatively stable (Supplemental Figure 2, A and B), consistent with the nonregenerative characteristic of RGCs. We then overexpressed *Ezh2* in RGCs by intravitreal injection of AAV2-*Ezh2*. Control mice were injected with AAV2-*GFP*. Previously, we showed that this approach could successfully transduce approximately 90% of RGCs (26, 28). Successful overexpression of *Ezh2* 2 weeks after virus injection was confirmed by immunoblotting of whole retinas or RGCs enriched from dissociated retinal cells by FACS (Supplemental Figure 3, A–D). In addition, immunofluorescence of retinal sections showed that H3K27me3 levels in RGCs consequently increased (Figure 3, A and B). Therefore, the optic nerve was crushed 2 weeks after virus injection. *Ezh2* overexpression improved RGC survival by approximately 50% 2 weeks after the ONC (Figure 3, C and D and Supplemental Figure 3E). In a different retinal injury model induced by intravitreal injection of N-methyl-D-aspartate (NMDA) (29), *Ezh2* overexpression almost doubled RGC survival 1 week after the excitotoxic injury to the retina (Figure 3, E and F), suggesting that Ezh2 could protect RGCs against various types of injury.

**Figure 4:**
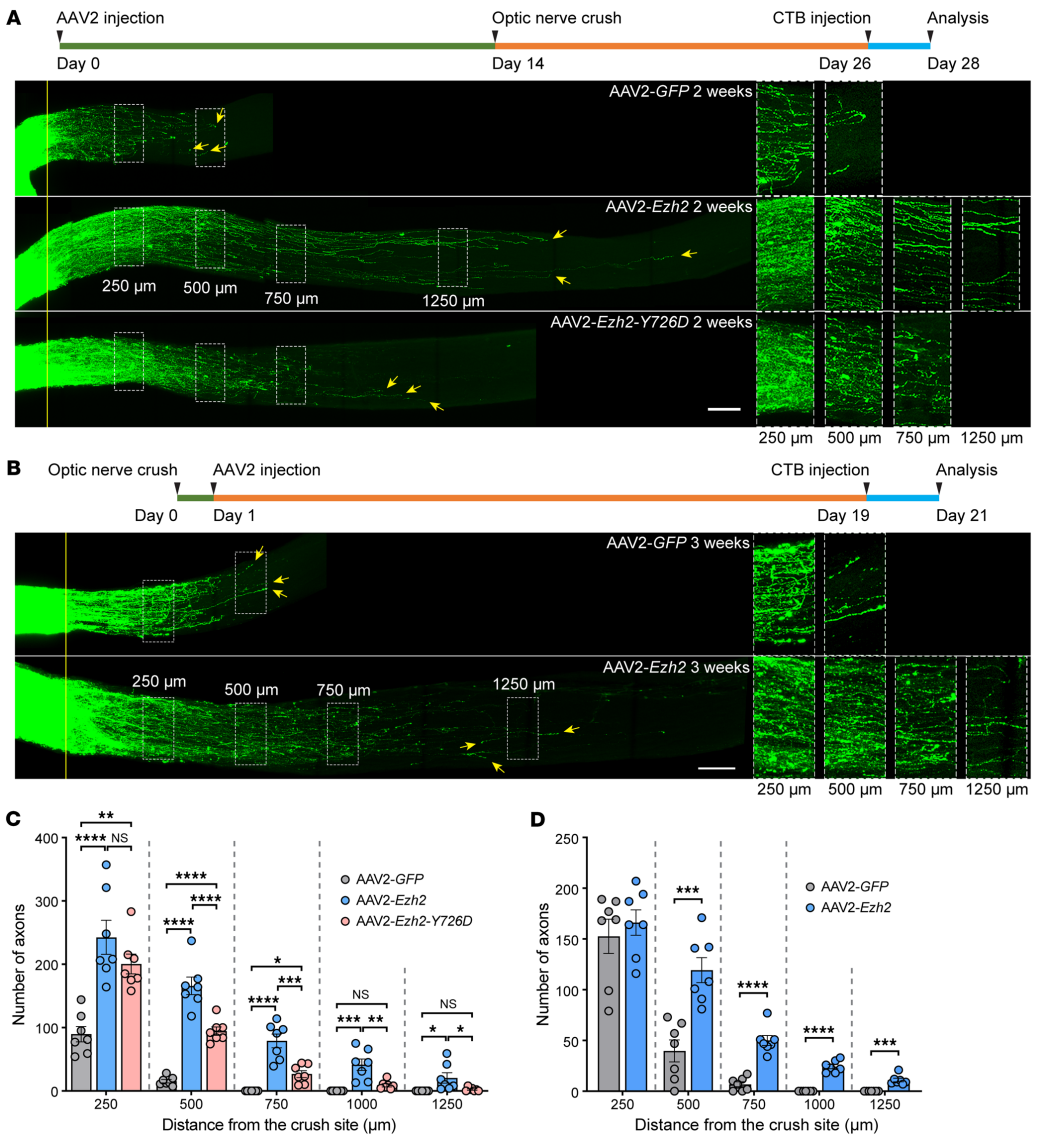
*Ezh2* overexpression enhances optic nerve regeneration via both histone methylation-dependent and -independent mechanisms. (**A** and **B**) Top: Experimental timeline. Bottom: Representative images of optic nerves showing that pre- (**A**) or postinjury (**B**) overexpression of *Ezh2* induces strong optic nerve regeneration 2 (**A**) or 3 weeks (**B**) after optic nerve crush. Preinjury overexpression of *Ezh2-Y726D* also modestly promotes optic nerve regeneration (**A**). Columns on the right display enlarged images of the areas in the white, dashed boxes on the left, showing axons at 250, 500, 750, and 1,250 μm distal to the crush sites, which are aligned with the yellow line. Yellow arrows indicate longest axons in each nerve. Scale bars: 100 μm, 50 μm for enlarged images. (**C**) Quantification of optic nerve regeneration in **A** (1-way ANOVA followed by Tukey’s multiple comparisons; *P* 0.0001 at 250, 500, 750, and 1,000 μm, *P* = 0.0126 at 1,250 μm; *n* = 7 mice for all). (**D**) Quantification of optic nerve regeneration in **B** (unpaired 2-tailed *t* test; *P* = 0.5305 at 250 μm, *P* = 0.0004 at 500 μm, *P* 0.0001 at 750 and 1,000 μm, *P* = 0.0003 at 1,250 μm; *n* = 7 mice for both). *P 0.05, **P 0.01, ***P 0.001, ****P 0.0001.

Optic nerve regeneration was also assessed 2 weeks after the ONC. Regenerating axons were labeled by Alexa Fluor–conjugated cholera toxin subunit B (CTB) intravitreally injected 2 days before tissue harvesting. Optic nerves were tissue-cleared and imaged as previously described (26, 28). Compared to the control group, where only a limited number of axons crossed the crush site, overexpression of *Ezh2* induced markedly enhanced optic nerve regeneration (Figure 4, A and C). Some axons grew over 1,250 μm in 2 weeks.

To investigate if the histone methyltransferase activity of Ezh2 was required for promoting optic nerve regeneration and RGC survival, we overexpressed a mutant form of Ezh2 with the 726th amino acid mutated from a tyrosine to an aspartic acid (Ezh2-Y726D) in RGCs (Supplemental Figure 3, A and C). Previous studies reported that this single amino acid mutation eliminated the methyltransferase activity of human and mouse Ezh2 (30, 31). Immunostaining of retinal sections confirmed that overexpression of *Ezh2-Y726D* did not increase H3K27me3 levels in RGCs (Figure 3, A and B). Surprisingly, this catalytically dead Ezh2 mutant exhibited comparable ability to enhance RGC survival as WT Ezh2 at 2 weeks after the ONC (Figure 3, C and D and Supplemental Figure 3E), suggesting a histone methylation–independent neuroprotective role of Ezh2. Moreover, Ezh2-Y726D also induced optic nerve regeneration, albeit to a much lesser extent (Figure 4, A and C), indicating that both histone methylation–dependent and –independent mechanisms contributed to promoting axon regeneration.

To explore the translational potential of *Ezh2* gain-of-function in CNS axon regeneration, we tested if postinjury overexpression of *Ezh2* in RGCs could also promote optic nerve regeneration. AAV2-*GFP* or AAV2-*Ezh2* was intravitreally injected 1 day after the ONC. 3 weeks later, we found that postinjury *Ezh2* overexpression still evidently promoted optic nerve regeneration (Figure 4, B and D), albeit weaker than that induced by preinjury overexpression of *Ezh2*. Specifically, more regenerating axons could be observed at 500–1,250 μm from the crush site after *Ezh2* overexpression. However, the numbers of regenerating axons at 250 μm from the crush site were equivalent between the 2 conditions. This was likely caused by the extended regeneration period in the control group (3 weeks in Figure 4, B and D versus 2 weeks in Figure 4, A and C) and delayed *Ezh2* expression in RGCs. These results demonstrated the translational potential of *Ezh2* gain-of-function for enhancing axon regeneration in the CNS.

### Ezh2 modifies the RGC transcriptome to regulate multiple categories of target genes.

To gain mechanistic insights into how Ezh2 supports RGC axon regeneration, we profiled the transcriptomic and epigenomic changes in RGCs induced by *Ezh2* or *Ezh2-Y726D* overexpression with RNA-Seq and assay for transposase-accessible chromatin with sequencing (ATAC-Seq). We intravitreally injected AAV2-*GFP* (control), AAV2-*Ezh2*, or AAV2-*Ezh2-Y726D* and crushed the optic nerve after 2 weeks. 3 days after the ONC, RGCs were enriched from dissociated retinal cells by FACS to construct RNA-Seq and ATAC-Seq libraries. All sequencing libraries exhibited high quality (Supplemental Figure 4, A–F and Supplemental Figure 5, A–S), except for 1 RNA-Seq library from the *Ezh2* overexpression condition, which was excluded from subsequent data analysis based on results of principle component analysis (PCA) and hierarchical clustering (Supplemental Figure 4, B and C). Chromatin accessibility at the promoter region moderately correlated with RNA expression within each condition (Supplemental Figure 6, A–D), suggesting consistency between the RNA-Seq and ATAC-Seq.

**Figure 5:**
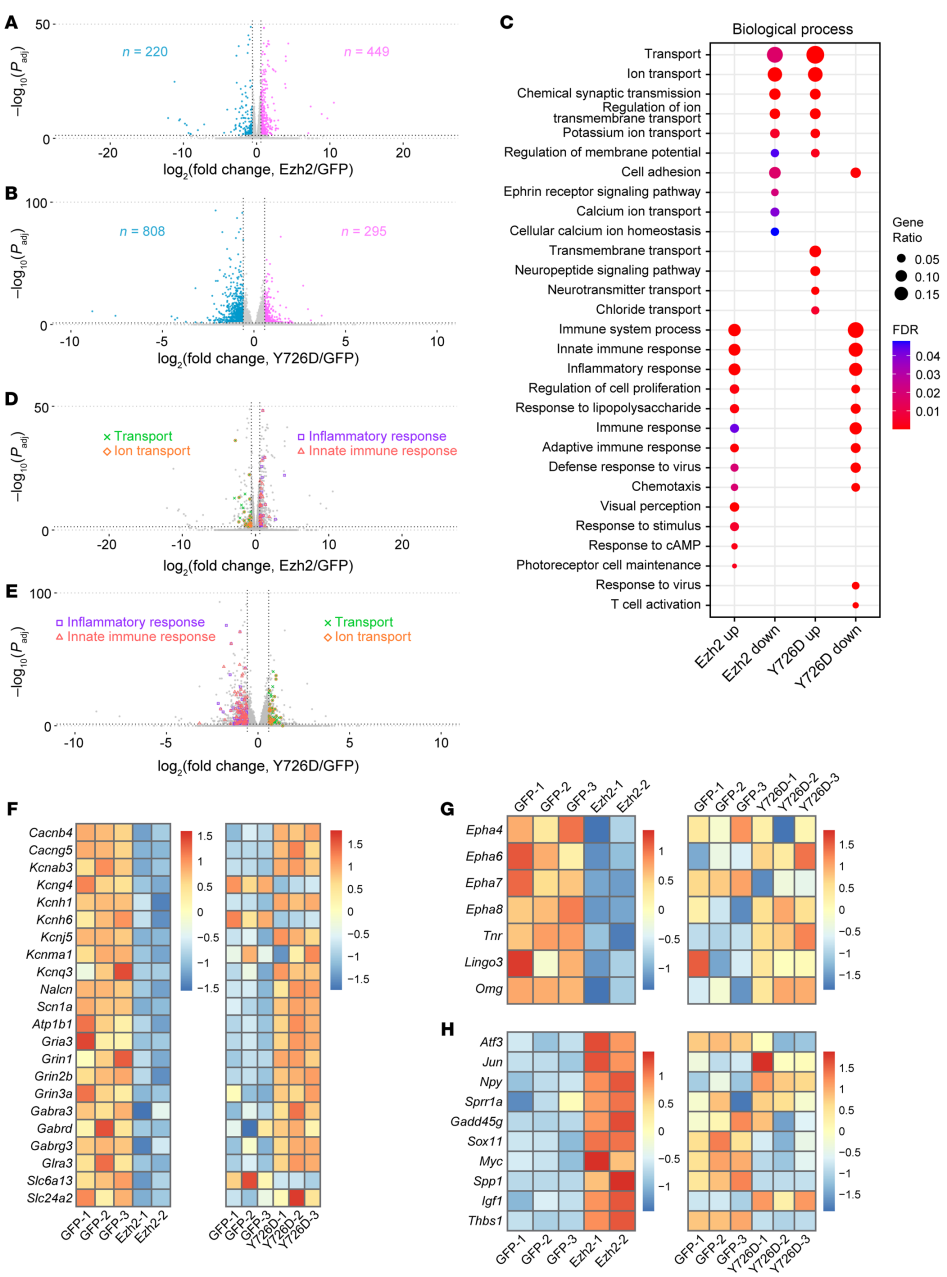
Ezh2 modifies the RGC transcriptome to regulate multiple categories of target genes. (**A** and **B**) Volcano plots showing differences in gene expression between control and *Ezh2* overexpression conditions (**A**) or between control and *Ezh2-Y726D* overexpression conditions (**B**). Note that 12 genes with –log_10_ (*P*_adj_) 50 and 3 genes with –log_10_(*P*_adj_) 100 are not plotted in **A** and **B**, respectively. (**C**) GO analysis of DEGs induced by *Ezh2* or *Ezh2-Y726D* overexpression. A subset of most significantly enriched GO terms in the biological process category are shown here. (**D** and **E**) Volcano plots described in **A** and **B** with DEGs in 4 enriched GO terms labeled. (**F** and **G**) Heatmaps of mRNA levels of neuronal excitability and synaptic transmission regulators (**F**) and axon regeneration inhibitory factors (**G**) downregulated by *Ezh2* overexpression in the control versus *Ezh2* overexpression RNA-Seq (left) and the control versus *Ezh2-Y726D* overexpression RNA-Seq (right). (**H**) Heatmaps of mRNA levels of axon regeneration positive regulators upregulated by *Ezh2* overexpression in the control versus *Ezh2* overexpression RNA-Seq (left) and the control versus *Ezh2-Y726D* overexpression RNA-Seq (right). Note that the control versus. *Ezh2* overexpression RNA-Seq and the control versus *Ezh2-Y726D* overexpression RNA-Seq were performed separately. Therefore, control (GFP) libraries in one RNA-Seq are independent of those in the other.

We identified 669 differentially expressed genes (DEGs) in the RNA-Seq between control and *Ezh2* overexpression conditions at the threshold of adjusted *P* less than 0.05 and fold change greater than 1.5 (Figure 5A, Supplemental Figure 4G, and Supplemental Table 1). Surprisingly, despite being a catalytically dead form of *Ezh2*, *Ezh2-Y726D* overexpression resulted in more DEGs (1,103, Figure 5B and Supplemental Table 1). This was consistent with our ATAC-Seq results, in which considerably more differentially accessible regions were found after *Ezh2-Y726D* overexpression (Supplemental Table 2), suggesting that unknown transcriptional regulatory activities of Ezh2, independent of the methyltransferase function, remain to be discovered. We then examined how the 669 DEGs induced by *Ezh2* overexpression were regulated by *Ezh2-Y726D* overexpression. Although not all of them were significantly regulated by Ezh2-Y726D, most showed opposite patterns of regulation after *Ezh2* or *Ezh2-Y726D* overexpression (Supplemental Figure 4G). Indeed, among the 236 common DEGs regulated by both Ezh2 and Ezh2-Y726D, 203 (86%) changed in opposite directions (Supplemental Figure 4H and Supplemental Table 1). Gene ontology (GO) analysis of the DEGs further revealed that Ezh2 and Ezh2-Y726D inversely modified the RGC transcriptome (Figure 5C and Supplemental Table 3). Specifically, *Ezh2* overexpression downregulated a series of ion transport and synaptic transmission-related genes and upregulated many immune response programs, both of which were oppositely regulated by *Ezh2-Y726D* overexpression (Figure 5, C–E). Similarly, in the ATAC-Seq, a large number of GO terms (immune response genes) were shared between genes whose promoter regions became more open after *Ezh2* overexpression and those whose promoter regions became more closed after *Ezh2-Y726D* overexpression (Supplemental Figure 6E and Supplemental Table 4). These results implied that Ezh2-Y726D might inhibit functions of endogenous Ezh2 in a dominant-negative manner.

Because Ezh2 primarily functions to repress gene transcription through H3K27me3, we first focused on genes downregulated by *Ezh2* overexpression. GO analysis showed that *Ezh2* overexpression decreased transcription of many genes coding for ion channels and transporters as well as neurotransmitter receptors and transporters (Figure 5, C, D, and F), which are all important regulators of neuronal excitability and synaptic transmission. Except for a few genes, most of them were upregulated by Ezh2-Y726D (Figure 5F), consistent with its dominant-negative role. Because neuronal excitability and synaptic transmission are fundamental biological functions of mature neurons, these results suggested that Ezh2 might turn mature RGCs to a developmental-like state at the transcriptomic level, favoring axon regeneration.

Close examination of the downregulated genes further revealed that Ezh2 suppressed transcription of multiple axon regeneration inhibitory factors or their receptors, including ephrin receptors (encoded by *Epha4*, *6*, *7*, and *8*; note that *Epha4* was not among the 669 DEGs but had an adjusted *P* value under 0.05), tenascin-R (encoded by *Tnr*), Lingo3, and oligodendrocyte myelin glycoprotein (OMgp; encoded by *Omg*) (Figure 5G). The available single-cell RNA-Seq data set of RGCs (27) or immunostaining of retinal sections confirmed their expression in RGCs (Supplemental Figure 7, A–H). Moreover, Ezh2-Y726D did not transcriptionally downregulate these genes (Figure 5G), suggesting H3K27me3-dependent regulation.

In addition to suppressing the above genes, *Ezh2* overexpression also upregulated many positive regulators of axon regeneration (Figure 5H). Among them, *Atf3*, *Jun*, *Npy*, *Sprr1a*, *Gadd45g*, and *Sox11* are well-known regeneration-associated genes (32–37). Others, including *Myc*, *Spp1* (which codes for osteopontin), *Igf1*, and *Thbs1* (which codes for thrombospondin-1), have been well documented to promote CNS axon regeneration (38–42). In contrast, *Ezh2-Y726D* overexpression downregulated many of these genes (Figure 5H), indicating that their upregulation by *Ezh2* overexpression was largely H3K27me3 dependent. Since H3K27me3 is associated with transcriptional suppression of its targets, these changes were likely secondary to increased H3K27me3.

### Ezh2 overexpression enhances optic nerve regeneration by downregulating GABA transporter 2.

**Figure 6:**
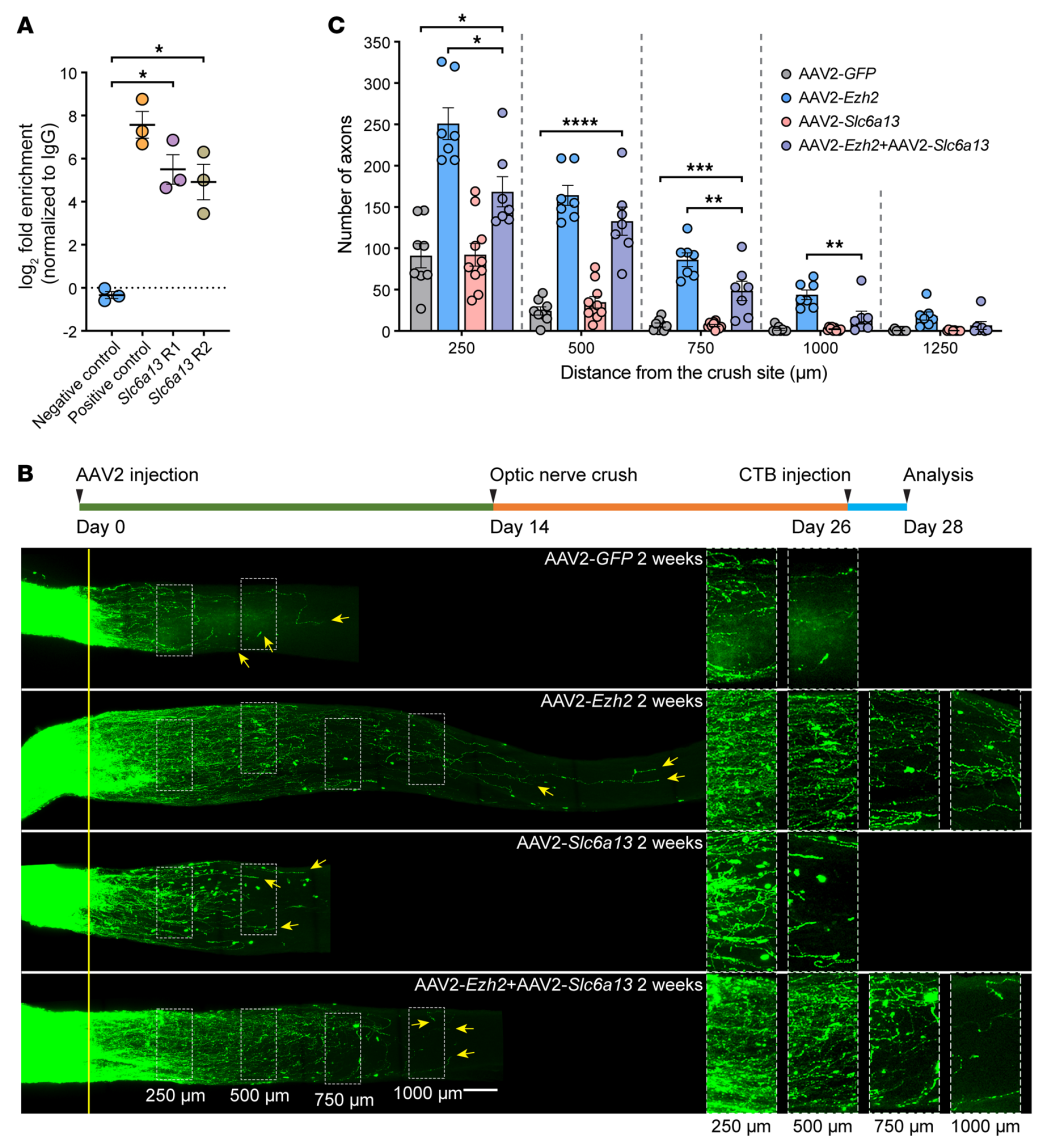
*Ezh2* overexpression enhances optic nerve regeneration by transcriptionally suppressing *Slc6a13*. (**A**) CUTTag followed by qPCR showing H3K27me3 enrichment in the promoter region of *Slc6a13* (paired 2-tailed *t* test; *P* = 0.0161 between negative control and *Slc6a13* R1, *P* = 0.0306 between negative control and *Slc6a13* R2; *n* = 3 independent experiments). (**B**) Top: Experimental timeline. Bottom: Representative images of optic nerves showing that *Slc6a13* overexpression partially blocks *Ezh2* overexpression–induced optic nerve regeneration. Columns on the right display enlarged images of the areas in the white, dashed boxes on the left, showing axons at 250, 500, 750, and 1,000 μm distal to the crush sites, which are aligned with the yellow line. Yellow arrows indicate longest axons in each nerve. Scale bars: 100 μm, 50 μm for enlarged images. (**C**) Quantification of optic nerve regeneration in **B** (1-way ANOVA followed by Tukey’s multiple comparisons; *P* 0.0001 at 250, 500, 750, and 1,000 μm, *P* = 0.0010 at 1,250 μm; *n* = 8 mice for control, *n* = 10 mice for *Slc6a13* overexpression, *n* = 7 mice for others). *P 0.05, **P 0.01, ***P 0.001, ****P 0.0001.

To determine if genes regulated by Ezh2 functionally acted downstream to regulate axon regeneration, we first examined the function of *Slc6a13* (which codes for GABA transporter 2 [Gat2]), one of the most significantly downregulated genes after *Ezh2* overexpression (see Figure 5F and Supplemental Table 1). We verified that *Slc6a13* was broadly expressed by RGCs (Supplemental Figure 7, I and J). Additionally, employing the cleavage under targets and tagmentation (CUTTag) method (43) followed by quantitative PCR (qPCR), we showed H3K27me3 enrichment at *Slc6a13* promoter region (Figure 6A), indicating its transcription could be directly regulated by Ezh2 via H3K27me3. Functionally, when *Slc6a13* was overexpressed along with *Ezh2* in RGCs by intravitreal injection of AAV2-*Slc6a13*, the strong optic nerve regeneration stimulated by *Ezh2* overexpression was partially blocked, whereas *Slc6a13* overexpression per se had no effect (Figure 6, B and C). These results demonstrated that *Slc6a13* downregulation was required, at least in part, by Ezh2 to enhance optic nerve regeneration, and suggested a pivotal role of extracellular GABA levels in regulating axon regeneration, in line with insights from several prior studies (44–46). *Slc6a13* overexpression also had a mild but significant inhibitory effect on regenerative axon growth of DRG neurons (Supplemental Figure 8, A and B), indicating its broadly consistent role in inhibiting axon regeneration.

**Figure 7:**
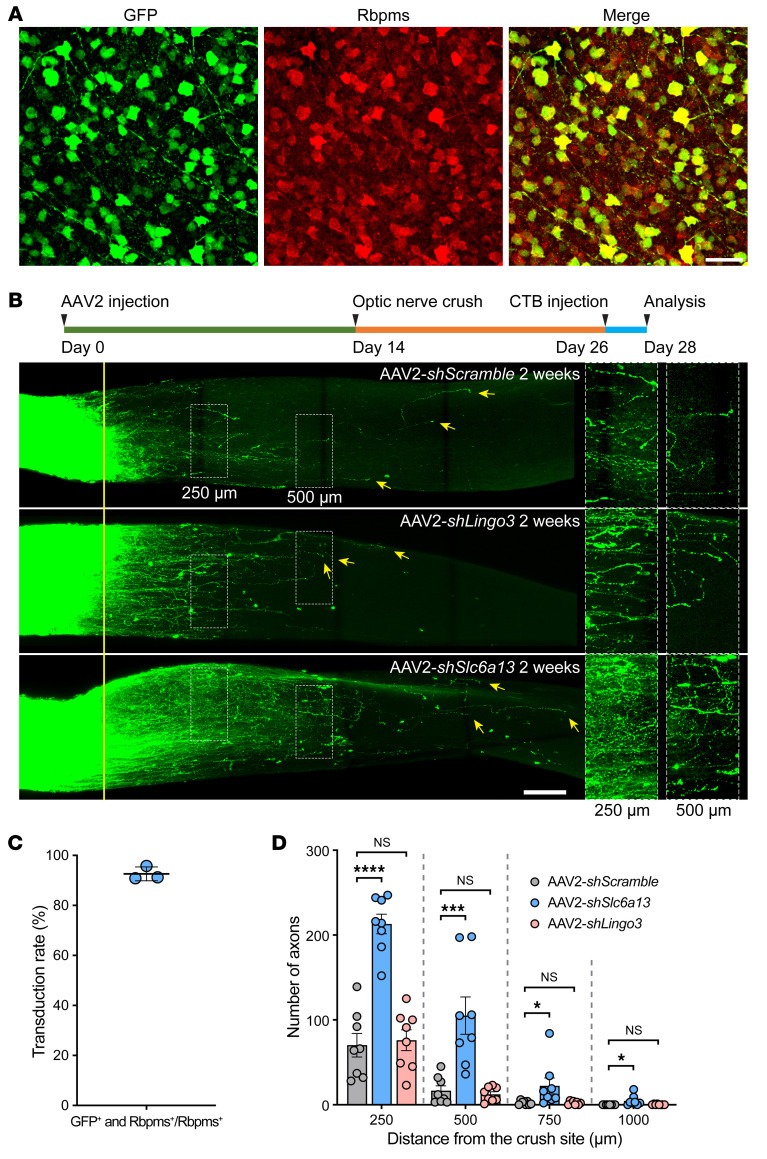
*Slc6a13* loss-of-function promotes optic nerve regeneration. (**A**) Representative immunofluorescence of whole-mount retinas showing high transduction efficiency of AAV2-*shSlc6a13-EGFP* in RGCs by intravitreal injection. Whole-mount retinas were stained with anti-GFP (green) and anti-Rbpms (red). Scale bar: 50 μm. (**B**) Top: Experimental timeline. Bottom: Representative images of optic nerves showing that knockdown of *Slc6a13*, but not *Lingo3*, modestly promotes optic nerve regeneration 2 weeks after optic nerve crush. Columns on the right display enlarged images of the areas in the white, dashed boxes on the left, showing axons at 250 and 500 μm distal to the crush sites, which are aligned with the yellow line. Yellow arrows indicate longest axons in each nerve. Scale bars: 100 μm, 50 μm for enlarged images. (**C**) Quantification of the percentage of GFP-positive RGCs in **A**. The average transduction rate was 92.65% ± 2.743% (*n* = 3 mice; 8 fields were analyzed for each mouse). Data represent mean ± SD. (**D**) Quantification of optic nerve regeneration in **B** (1-way ANOVA followed by Tukey’s multiple comparisons; *P* 0.0001 at 250 and 500 μm, *P* = 0.0248 at 750 μm, *P* = 0.0263 at 1,000 μm; *n* = 8 mice for all). *P 0.05, ***P 0.001, ****P 0.0001.

Based on these results, we wondered if downregulation of *Slc6a13* could sufficiently induce optic nerve regeneration. We knocked down *Slc6a13* in RGCs by intravitreal injection of AAV2 vectors encoding an shRNA against *Slc6a13* mRNA. The transduction efficiency in RGCs was 92.65% ± 2.743% (Figure 7, A and C). We found that *Slc6a13* loss-of-function effectively induced optic nerve regeneration (Figure 7, B and D). Together, these results demonstrated that *Slc6a13* was a key downstream target of Ezh2 that mediates axon regeneration.

### Ezh2 overexpression enhances optic nerve regeneration by suppressing major axon regeneration inhibitory signaling.

**Figure 8:**
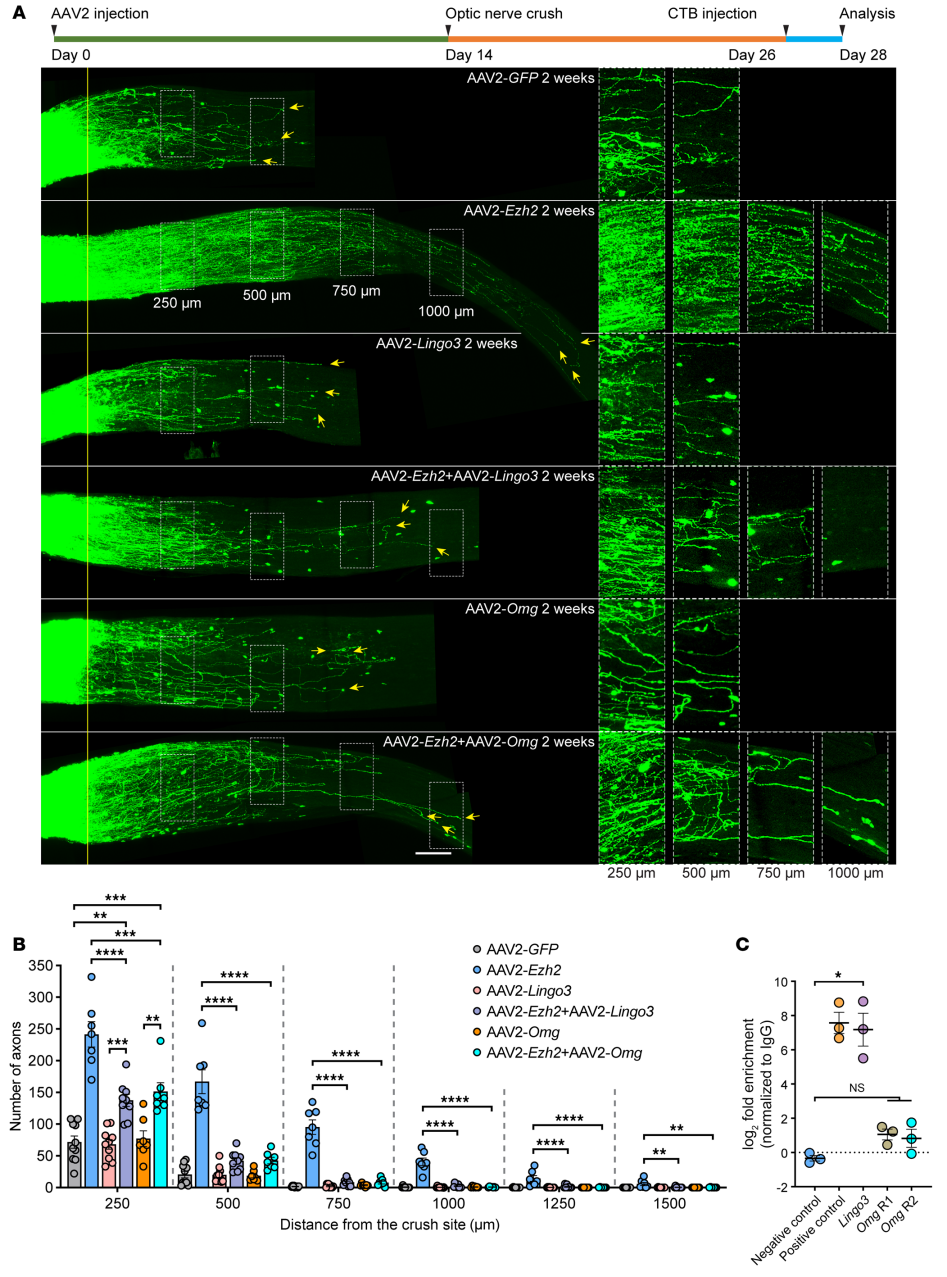
*Ezh2* overexpression enhances optic nerve regeneration by transcriptionally suppressing *Lingo3* and *Omg*. (**A**) Top: Experimental timeline. Bottom: Representative images of optic nerves showing that *Lingo3* or *Omg* overexpression almost completely blocks *Ezh2* overexpression-induced optic nerve regeneration. Columns on the right display enlarged images of the areas in white, dashed boxes on the left, showing axons at 250, 500, 750, and 1,000 μm distal to the crush sites, which are aligned with the yellow line. Yellow arrows indicate longest axons in each nerve. Scale bars: 100 μm, 50 μm for enlarged images. (**B**) Quantification of optic nerve regeneration in **A** (1-way ANOVA followed by Tukey’s multiple comparisons; *P* 0.0001 at 250, 500, 750, 1,000, and 1,250 μm, *P* = 0.0003 at 1,500 μm; *n* = 10 mice for control and *Lingo3* overexpression, *n* = 9 mice for *Ezh2* and *Lingo3* cooverexpression, *n* = 7 mice for others). (**C**) CUTTag followed by qPCR showing H3K27me3 enrichment in the promoter region of *Lingo3*, but not that of *Omg* (paired 2-tailed *t* test; *P* = 0.0197 between negative control and *Lingo3* R1, *P* = 0.1010 between negative control and *Omg* R1, *P* = 0.2329 between negative control and *Omg* R2; *n* = 3 independent experiments). Note that the negative control and positive control are identical to those in Figure 6A. *P 0.05, **P 0.01, ***P 0.001, ****P 0.0001.

Next, we tested if downregulation of axon regeneration inhibitory signaling contributed to *Ezh2* overexpression–induced optic nerve regeneration. Overexpression of *Omg* or *Lingo3*, per se, had no effect on optic nerve regeneration, but almost completely blocked *Ezh2* overexpression–induced regeneration (Figure 8, A and B). Only at 250 μm from the crush site were more axons observed in the cooverexpression groups than in the control group. CUTTag followed by qPCR revealed H3K27me3 binding at the *Lingo3* promoter region (Figure 8C), suggesting H3K27me3-mediated transcriptional repression of *Lingo3*. Unlike *Slc6a13*, *Lingo3* loss-of-function did not promote optic nerve regeneration (Figure 7, B and D). In contrast, no binding of the *Omg* promoter region by H3K27me3 was detected (Figure 8C), suggesting that *Omg* downregulation might be a secondary effect of elevated H3K27me3. Collectively, these results suggested that downregulation of *Lingo3* and *Omg* contributed to optic nerve regeneration induced by *Ezh2* overexpression, and that Ezh2 might be a key suppressor of signaling pathways that impede CNS axon regeneration (see Discussion).

### Ezh2 activates multiple axon regeneration enhancing pathways.

**Figure 9:**
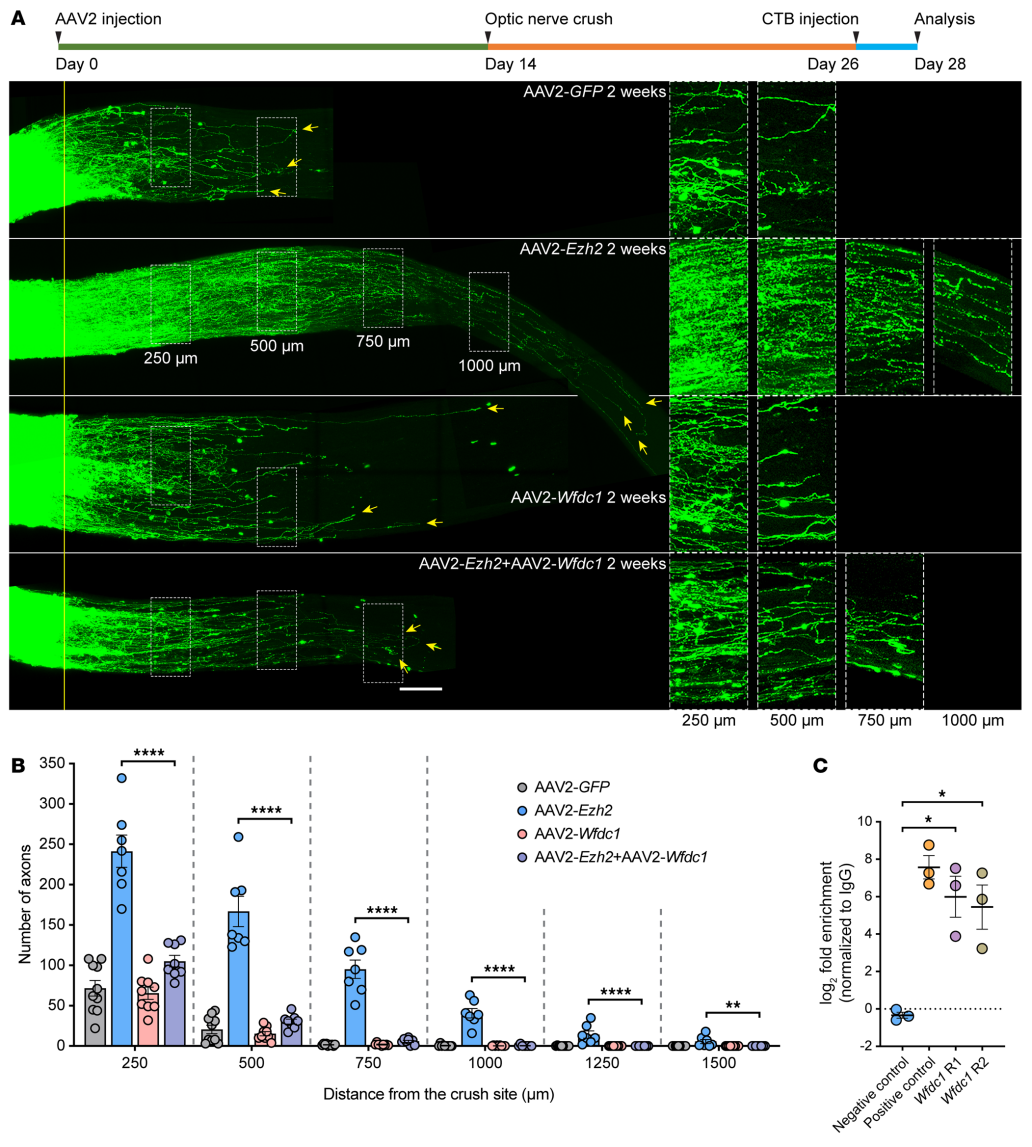
*Ezh2* overexpression enhances optic nerve regeneration by transcriptionally suppressing *Wfdc1*. (**A**) Top: Experimental timeline. Bottom: Representative images of optic nerves showing that *Wfdc1* overexpression completely blocks *Ezh2* overexpression–induced optic nerve regeneration. Columns on the right display enlarged images of the areas in the white, dashed boxes on the left, showing axons at 250, 500, 750, and 1,000 μm distal to the crush sites, which are aligned with the yellow line. Yellow arrows indicate longest axons in each nerve. Scale bars: 100 μm, 50 μm for enlarged images. (**B**) Quantification of optic nerve regeneration in **A** (1-way ANOVA followed by Tukey’s multiple comparisons; *P* 0.0001 at 250, 500, 750, 1,000, and 1,250 μm, *P* = 0.0015 at 1,500 μm; *n* = 10 mice for control, *n* = 7 mice for *Ezh2* overexpression, *n* = 9 mice for *Wfdc1* overexpression, *n* = 8 mice for *Ezh2* and *Wfdc1* cooverexpression). Note that the control and *Ezh2* overexpression conditions are identical to those in Figure 8B. (**C**) CUTTag followed by qPCR showing H3K27me3 enrichment in the promoter region of *Wfdc1* (paired 2-tailed *t* test; *P* = 0.0357 between negative control and *Wfdc1* R1, *P* = 0.0478 between negative control and *Wfdc1* R2; *n* = 3 independent experiments). Note that the negative control and positive control are identical to those in Figure 6A. *P 0.05, **P 0.01, ***P 0.001, ****P 0.0001.

*Ezh2* overexpression in RGCs resulted in upregulation of osteopontin (encoded by *Spp1*, see Figure 5H and Supplemental Table 1), which selectively promotes axon regeneration of αRGCs (40). In addition to retinal repair, increased osteopontin expression was also shown to underlie the enhanced tissue repair induced by knocking out *Wfdc1* (47), known as a tumor suppressor (48) and a wound repair inhibitor (47). Interestingly, mRNA levels of *Wfdc1* in RGCs were also reduced by Ezh2 in our RNA-Seq (see Supplemental Table 1), suggesting that it might regulate Ezh2-induced optic nerve regeneration via osteopontin. Indeed, overexpression of *Wfdc1* completely blocked the optic nerve regeneration induced by *Ezh2* overexpression (Figure 9, A and B), suggesting that Wfdc1 was a strong axon regeneration inhibitor transcriptionally suppressed by Ezh2. CUTTag followed by qPCR showed that the *Wfdc1* promoter region was bound by H3K27me3 (Figure 9C), again suggesting histone methylation–dependent regulation by Ezh2. These results provided a potential mechanism of how Ezh2 indirectly upregulated axon regeneration–enhancing factors.

Among other upregulated genes (Supplemental Table 1), *Ascl1* and *Neurog2* are important regulators of neurogenesis and axon guidance during development and direct reprogramming factors converting glial cells into neurons (49). Moreover, Ascl1, Neurog2, and Ezh2 were identified as key factors driving neuronal differentiation in a Crispr-based screening (50), suggesting similar or cooperative functions. Interestingly, Ascl1 was shown to support PNS axon regeneration in mice (51) and CNS axon regeneration in zebrafish and rats (52). We therefore investigated if Neurog2 could also regulate optic nerve regeneration. Overexpression of *Neurog2* in RGCs had little effect on optic nerve regeneration (Supplemental Figure 9, A and B), suggesting that distinct mechanisms mediate neurogenesis and axon regeneration.

### Ezh2 overexpression does not alter the epigenetic aging clock of RGCs.

A recent study discovered that ONC increased the DNA methylation age of RGCs, and that polycistronic expression of reprogramming factors Oct4, Sox2, and Klf4 counteracted the aging effect of ONC and promoted optic nerve regeneration (53). Ezh2 is also critical for efficient cellular reprogramming (54, 55) and was shown to participate in shaping the aging epigenome (56). Moreover, we showed that *Ezh2* overexpression specifically silenced transcription of many genes functionally involved in synaptic activities of mature neurons, in some way turning adult RGCs to a developmental-like state at the transcriptomic level. We thus wondered if *Ezh2* overexpression could also reverse the aging effect of ONC on the RGC epigenome. We intravitreally injected AAV2-*GFP*, AAV2-*Ezh2*, or AAV2-*Ezh2-Y726D* into mice of exactly the same age, performed ONC 2 weeks after the injection, and extracted DNA from FACS-enriched RGCs 3 days after the ONC. Uninjured groups only received AAV2 injection but did not undergo ONC. Reduced representation bisulfite sequencing (RRBS) libraries were constructed from RGC DNA and sequenced to obtain the DNA methylation landscape. A predictive PCA model (53) was used to estimate changes in the DNA methylation age of RGCs. The results confirmed that ONC accelerated epigenetic aging of RGCs, but neither WT Ezh2 nor Ezh2-Y726D could reverse the changes (Supplemental Figure 10A). Consistently, our RNA-Seq did not detect significant changes in mRNA levels of most 5mC DNA methyltransferases or demethylases (Supplemental Figure 10, B and C). These results indicated that *Ezh2* overexpression was not able to rejuvenate mature RGCs epigenetically.

Collectively, our study not only revealed a role of Ezh2 in coordinating axon regeneration via regulation of multiple key regenerative pathways, but also identified chromatin accessibility as a promising target to promote CNS axon regeneration.

## Discussion

### Developmental decline of axon regeneration ability with transcriptomic changes regulated by Ezh2.

Axon regeneration ability of mammalian neurons declines as they mature. While PNS neurons can reactivate their regenerative ability upon peripheral axonal injury, most adult CNS neurons permanently lose their ability to regenerate axons. Given that every single cell in an organism has completely the same genome, and so does a neuron in different states (e.g., developmental versus mature or healthy versus injured), it is conceivable that the tuning of the axon regeneration ability in neurons is largely regulated by changes in the epigenomic and transcriptomic landscapes. Here we found that Ezh2, an epigenetic regulator that controls chromatin accessibility and gene transcription via histone methylation, was developmentally downregulated in both the PNS and CNS and could be upregulated in PNS neurons by peripheral nerve injury. Thus, Ezh2 levels in the nervous system and the axon growth and regeneration potential of neurons rise and fall in parallel. Indeed, we found that *Ezh2* loss-of-function impaired spontaneous axon regeneration of mature PNS neurons, while *Ezh2* gain-of-function promoted axon regeneration in nonregenerative adult CNS neurons. Mechanistic exploration revealed that *Ezh2* overexpression in RGCs suppressed transcription of a large number of genes regulating synaptic transmission and neuronal excitability, which are housekeeping functions of mature neurons. Therefore, our study suggested that Ezh2 upregulation might turn mature neurons into a developmental-like cellular state at the transcriptomic level to empower them with stronger axon regeneration ability. In support of this, several previous studies also found a negative correlation between synaptic functions and axon regeneration ability (44, 57–59). On the other hand, *Ezh2* overexpression also resulted in upregulation of many factors known to enhance axon regeneration, some of which are highly expressed in developing neurons (60–62).

When the epigenetic aging biomarker, the DNA methylation clock, was examined in RGCs (53), we confirmed that optic nerve injury significantly accelerated RGC epigenetic aging. However, *Ezh2* overexpression was not able to reverse it. These results indicated that physiological aging at the transcriptomic level could be uncoupled from the DNA methylation aging clock. The discrepancy between DNA methylation–based aging clock and transcriptomic landscape–based cellular state was not surprising. A recent study (63) of naked mole rats (NMRs), which live an exceptionally long life and are considered a nonaging mammal, showed a normal aging progress in many tissues at the epigenetic level without significant overlap with age-related transcriptomic changes. Interestingly, cell reprogramming was able to rejuvenate the DNA methylation clock of NMR cells, consistent with a recent study in which partial reprogramming of RGCs with 3 reprogramming factors, Oct4, Sox2, and Klf4, reversed the DNA methylation aging induced by optic nerve injury (53). Collectively, we think that *Ezh2* overexpression in mature RGCs switched their transcriptomic landscape to a developmental-like state with stronger axon growth ability.

### Ezh2 is a master suppressor of CNS axon regeneration inhibitory signaling.

Here, we demonstrated that *Ezh2* overexpression transcriptionally silenced OMgp (1 of the 3 major MAIs), Lingo3, tenascin-R, and several ephrin receptors. OMgp and other MAIs (MAG and Nogo) act through the Nogo receptor 1 (NgR1) complex or PirB to inhibit axon growth (64–66). *Lingo3* is a paralog of *Lingo1* that codes for a critical component of the NgR1 complex (67), which blocks axon regeneration via RhoA when activated by MAIs and CSPGs (68). *Lingo1* loss-of-function also promotes axon regeneration and neuronal survival in various CNS injury and disease models (69–71). Additionally, Lingo family receptors can form heteromers with one another in the mouse brain (72), strongly suggesting functional overlap between the paralogs. Tenascin-R, an extracellular matrix molecule, is a repulsive guidance cue in zebrafish during development (73) and an inhibitor of mouse optic nerve regeneration (74). Ephrin receptors are chemorepellent axon guidance molecules that can cause growth cone collapse when activated by their ligands, ephrins (75). MAIs, CSPGs, and repulsive axon guidance cues are 3 major classes of extracellular axon regeneration inhibitors in the mature CNS (76). Ezh2 downregulated transcription of regeneration inhibitors or their receptors associated with all 3 classes. Functionally, we found that overexpression of *Omg* or *Lingo3* blocked optic nerve regeneration induced by *Ezh2* overexpression to a great extent. Thus, Ezh2 might be a master suppressor of CNS axon regeneration inhibitory signaling pathways. Notably, our study indicated that, besides glial cells, neurons per se could also contribute to the production of extracellular CNS regeneration inhibitors, such as OMgp and tenascin-R. It would be interesting for future studies to investigate the mechanisms of neuron-secreted axon regeneration inhibitors.

### Ezh2 enhances optic nerve regeneration via both methylation-dependent and -independent pathways.

Most previous studies of Ezh2 focused on H3K27me3-mediated transcriptional repression in various biological processes. Evidence has been emerging, however, to suggest that Ezh2 also has activities unrelated to protein methylation. For example, Ezh2 can transactivate the androgen receptor by directly binding to its promoter region (20). Similarly, a ternary complex of Ezh2, RelA, and RelB bind to promoters of *Il6* and *Tnf* to enhance their transcription (19). Furthermore, Ezh2 can even regulate protein translation via interacting with fibrillarin and controlling rRNA methylation, completely independent of its methyltransferase function (77). In our study, an Ezh2 mutant lacking the methyltransferase activity, Ezh2-Y726D, was still able to modestly promote optic nerve regeneration, clearly indicating that methyltransferase-independent activities of Ezh2 also contributed to the enhanced axon regeneration.

In the current study, we did not further investigate these methyltransferase-independent mechanisms. Our RNA-Seq results implied that Ezh2-Y726D acted in a dominant-negative manner of WT Ezh2 to control gene transcription. Such results appeared perplexing, as Ezh2-Y726D still promoted RGC survival and optic nerve regeneration. A likely explanation is that the overall effect of Ezh2-Y726D overexpression was to enhance the maturation state of RGCs, which was unlikely to further reduce the already very low intrinsic axon growth ability. However, Ezh2-Y726D did regulate some genes in the same way as Ezh2. For example, both *Ezh2* and *Ezh2-Y726D* overexpression significantly upregulated *Jun*, *Npy*, and *Igf1*, all of which have been shown to support axon regeneration (78–80). Likewise, both Ezh2 and Ezh2-Y726D downregulated mRNA levels of corticotropin releasing hormone binding protein (encoded by *Crhbp*) and *Slc6a13* (see Figure 5F and Supplemental Table 1). *Crhbp* is selectively expressed in RGC subtypes susceptible to ONC, and its loss-of-function significantly promotes RGC survival and optic nerve regeneration (27). Similarly, our current study showed that knocking down *Slc6a13* induced optic nerve regeneration (see Figure 7, B and D). Upregulation of axon regeneration enhancers and downregulation of *Crhbp* and *Slc6a13* might be methylation-independent mechanisms by which Ezh2-Y726D modestly promoted optic nerve regeneration. Future studies are needed to further explore the roles noncanonical pathways of Ezh2 play in mammalian axon regeneration.

Although AAV2 also transduces other retinal cells besides RGCs, our data indicated that *Ezh2* overexpression–induced increase of H3K27me3 was mostly observed in cells within the ganglion cell layer of the retina (see Figure 3A), which contains mainly RGCs and some displaced amacrine cells. However, we cannot exclude the possibility that non-RGC-autonomous factors also contributed to the optic nerve regeneration observed here (80). Future studies using *Vglut2-Cre* mice to restrict *Ezh2* expression in RGCs would provide a clearer answer.

## Methods

### Mice.

Adult C57BL/6J mice (6–10 weeks old) of both sexes were used unless otherwise stated. The *Ezh2^fl/fl^* (stock no. 015499-UNC) mouse strain was obtained from the Mutant Mouse Resource and Research Center (MMRRC) at University of North Carolina at Chapel Hill, an NIH-funded strain repository, and was donated to the MMRRC by Alexander Tarakhovsky (the Rockefeller University [New York, New York, USA]). The *Advillin-Cre* mouse line (the Jackson Laboratory) stock no. 032536) was a gift from Fan Wang at Duke University in Durham, North Carolina, and was crossed with *Ezh2^fl/fl^* to obtain *Advillin-Cre;Ezh2^fl/fl^* conditional knockout mice. Because female *Advillin-Cre* mice have weak Cre expression in oocytes, only male *Advillin-Cre^tg/+^;Ezh2^fl/fl^* mice and female *Ezh2^fl/fl^* mice were used for breeding. Therefore, the resulting offspring was either heterozygous or negative for *Advillin-Cre*. Both male and female *Advillin-Cre^tg/+^;Ezh2^fl/fl^* mice were used for experiments. Genotypes of the mice were determined by PCR using primers and programs provided by the MMRRC and the Jackson Laboratory. All mouse surgeries were performed under anesthesia induced by i.p. injection of ketamine (100 mg/kg) and xylazine (10 mg/kg) diluted in sterile saline. Details of surgeries are described below.

### Immunoblotting.

Total protein was extracted from mouse DRGs, cultured DRG cells, retinas, or FACS-enriched RGCs using the RIPA buffer (Sigma-Aldrich) containing the protease inhibitor cocktail (Sigma-Aldrich) and phosphatase inhibitor cocktail (Sigma-Aldrich). Identical amounts of total protein from each condition were separated by SDS-PAGE on 4-to-12% Bis-Tris gels and transferred onto polyvinylidene difluoride membranes. Membranes were blocked with TBST containing 5% blotting-grade blocker (Bio-Rad), incubated in primary antibodies against target molecules overnight at 4°C, washed 4 times (5, 5, 10, and 10 minutes) with TBST, incubated in corresponding HRP-linked secondary antibodies (1:2,000; Cell Signaling Technology 7074 or 7076) for 1 hour at room temperature, and washed 4 times (5, 5, 10, and 10 minutes) again with TBST. All antibodies were diluted with TBST containing 5% blotting-grade blocker. Primary antibodies used for immunoblotting in this study include rabbit anti-Ezh2 (1:1,000, Cell Signaling Technology 5246), rabbit anti-H3 (1:1,000, Cell Signaling Technology 9715), mouse anti-H3K27me3 (1:10,000, Sigma-Aldrich 05-1951), rabbit anti-Ezh1 (1:2,000, Sigma-Aldrich ABE281), rabbit anti-Suz12 (1:1,000, Cell Signaling Technology 3737), rabbit anti-Eed (1: 1,000, Sigma-Aldrich 09-774), rabbit anti-Rbap46/48 (1:1,000, Active Motif 39199), mouse anti-β-actin (1:10,000, Sigma-Aldrich A1978), and mouse anti-Gapdh (1:20,000, Sigma-Aldrich G8795). See complete unedited blots in the supplemental material.

### In vivo DRG electroporation.

Under anesthesia, a small dorsolateral laminectomy was performed on the left side to expose left L4/5 DRGs. Using a pulled glass micropipette (World Precision Instruments) connected to a Picospritzer III (pressure: 20 psi, pulse duration: 6 ms, Parker Hannifin), 1 μL plasmid vectors (2 μg/μL) and/or si*Ezh2* (100 μM, Horizon Discovery, see Supplemental Table 5 for target sequences) containing 0.05% fast green FCF (Sigma-Aldrich) were injected into each DRG. After injection, in vivo electroporation was performed by applying 5 electric pulses (voltage: 35 V, pulse duration: 15 ms, pulse interval: 950 ms) using a platinum tweezertrode (BTX) powered by an ECM 830 Electro Square Porator (BTX). The wound was then closed with sutures.

### Sciatic nerve crush or transection.

Under anesthesia, sciatic nerves were exposed right below the pelvis and crushed with Dumont #5 forceps (Fine Science Tools) for 15 seconds or cut with scissors, and the wound was closed by sutures. In sham surgeries, sciatic nerves were only exposed but not injured. Both sciatic nerve transection and sciatic nerve crush result in axotomy of all axons in the sciatic nerve. Sciatic nerve crush was performed in in vivo DRG neuron axon regeneration experiments and was only done on the left side. The crush site was marked with 10-0 nylon epineural sutures that are identifiable during dissection and imaging data analysis. Sciatic nerve transection was performed in other experiments to ensure completeness of the axotomy and was done bilaterally.

### Analysis of in vivo DRG neuron axon regeneration.

Three or 5 days after sciatic nerve crush, mice were anesthetized and transcardially perfused with PBS followed by 4% PFA. Sciatic nerve segments (proximal end: 5 mm proximal to the crush site; distal end: the point where the sciatic nerve branches into 3 nerves) were dissected and postfixed in 4% PFA overnight at 4°C. The next day, nerve segments were mounted in Fluoroshield (Sigma-Aldrich) onto microscope slides, covered with coverslips, and flattened by applying a heavy weight on coverslips. Tiled fluorescent images of whole-mount nerve segments were obtained with a Zeiss inverted fluorescence microscope controlled by the AxioVision software using a 5× objective. Nerve segments were imaged from approximately 1 mm proximal to the crush site to approximately 0.5 mm distal to the end of the longest axon. Using the built-in “measure/curve spline” function of the AxioVision software, GFP-labeled axons were manually traced from the crush site to axonal tips to determine the lengths. The mean length of all axons traced in 1 nerve segment was used as the average axon length of this nerve. Nerves whose epineural sutures were missing or with less than 10 identifiable GFP-labeled axons were excluded from data analysis. Measurement was done by experimenters blinded to experimental conditions. Nerve images were put on a black background when figures were generated.

### ONC and regeneration.

Intravitreal virus injection, ONC, and RGC axon labeling were performed as previously described (28). Briefly, under anesthesia, 1.5 μL AAV2 virus (approximately 1 × 10^13^ genome copies/mL) was injected into the vitreous humor with a Hamilton syringe (33-gauge needle). The position and direction of the needle were well controlled to avoid injury to the lens. Two weeks after the virus injection, under anesthesia, a small incision was made in the skin right behind the eye and the conjunctiva was incised to expose the extraocular muscles. The muscles were pushed aside with forceps to expose the optic nerve, and the optic nerve was crushed with Dumont #5 forceps (Fine Science Tools) for 5 seconds at approximately 0.5 mm behind the optic disc. Care was taken to avoid damage to the ophthalmic artery. For the postinjury treatment model, ONC was done 1 day before virus injection. To label RGC axons in the optic nerve, under anesthesia, 1.5 μL Alexa Fluor 555 or 647–conjugated CTB (1 μg/μL, Thermo Fisher Scientific C22843 or C34778) was injected into the vitreous humor with a Hamilton syringe (33-gauge needle) 2 days before tissue harvesting. Mice with lens injury, hemorrhage, or incomplete crush evidenced by continuous CTB labeling through the chiasm were excluded from data analysis.

### Analysis of optic nerve regeneration.

Z-stacked (step size: 2 μm) and tiled fluorescent images of tissue-cleared whole-mount optic nerves were obtained with a Zeiss LSM 800 confocal microscope using a 20× objective. Optic nerves were imaged from approximately 0.5 mm proximal to the crush site to approximately 0.5 mm distal to the end of the longest axon. To quantify the number of regenerating axons in each optic nerve, every 8 consecutive planes were Z-projected in maximum intensity to generate a series of Z-projection images of 16-μm-thick optical sections. At each 250-μm interval from the crush site, the number of CTB-labeled axons was counted in each Z-projection image and summed over all optical sections. Nerve images were put on a black background when figures were generated.

### NMDA-induced excitotoxicity model.

Under anesthesia, 1.5 μL AAV2 virus (approximately 1 × 10^13^ genome copies/mL) was injected into the vitreous humor with a Hamilton syringe (33-gauge needle). Two weeks after the virus injection, under anesthesia, 1.5 μL NMDA (20 mM, Sigma-Aldrich) was injected into the vitreous humor with a Hamilton syringe (33-gauge needle). The position and direction of the needle were well controlled to avoid injury to the lens.

### RGC enrichment.

Retinas were dissected from euthanized mice, digested with papain (Thermo Fisher Scientific 88285) containing 0.005% DNase (Worthington) at 37°C for 8 minutes, washed 3 times with HBSS, and dissociated into cell suspension by trituration in NeuroBasal medium containing 1% BSA. Cells were filtered with a 40 μm cell strainer, pelleted by centrifugation (500*g* for 5 minutes at room temperature), resuspended in NeuroBasal medium containing 1% BSA, blocked with rat anti-mouse CD16/CD32 (1:50, BD Biosciences 553141) for 5 minutes on ice, and labeled with PE-conjugated rat anti-CD90.2 (1:100, Thermo Fisher Scientific 12-0902-81) for 30 minutes on ice. After that, cells were washed twice with HBSS containing 1% BSA, pelleted by centrifugation (500*g* for 5 minutes at room temperature), and again resuspended in Neurobasal medium containing 1% BSA. Propidium iodide (PI) or DAPI was mixed with the cell suspension to label dead cells 2 minutes before cells were loaded into a Beckman Coulter MoFlo Legacy Cell Sorter. CD90.2-positive and PI or DAPI–negative cells were sorted into NeuroBasal medium containing 1% BSA with a 70 μm nozzle.

### CUTTag and qPCR.

ChIP-Seq libraries were constructed from FACS-enriched RGCs (100,000 cells for each library) using rabbit anti-H3K27me3 (1:50, Active Motif 39155) or normal rabbit IgG (1:50, Sigma-Aldrich NI01) and the CUTTag-IT Assay Kit (Active Motif) following the manufacture’s manual. Identical amounts of DNA from each library were used in qPCR to determine the enrichment of H3K23me3 in the promoter region of each gene. Two pairs of primers were designed for each gene. One pair for *Lingo3* promoter region resulted in no amplification and was excluded from data analysis. Sequences of primers used in qPCR are in Supplemental Table 5. Positive control (71020) and negative control primers (71013) were purchased from Active Motif. Fold enrichment of H3K27me3 binding was determined using the ddCt method and normalized to IgG. All qPCR experiments were done in triplicate.

### Immunofluorescence of whole-mount retinas.

Retinas were dissected from transcardially perfused mice and postfixed in 4% PFA overnight at 4°C. On the next day, retinas were postfixed in ice-cold methanol for 20 minutes, washed 3 times for 5 minutes each time with PBS, radially cut into a petal shape, and blocked with PBST (1%) containing 10% goat serum for 1 hour at room temperature. After blocking, retinas were incubated in primary antibodies against target molecules overnight at 4°C, washed 4 times for 15 minutes each time with PBST (0.3%), incubated in corresponding Alexa Fluor–conjugated secondary antibodies (1:500, Thermo Fisher Scientific) for 2 hours at room temperature, and washed 4 times for 15 minutes each time again with PBST (0.3%). All antibodies were diluted with PBST (1%) containing 10% goat serum. Retinas were flat-mounted in Fluoroshield (Sigma-Aldrich) onto microscope slides and covered by coverslips. Fluorescent images of whole-mount retinas were obtained with a Zeiss LSM 800 confocal microscope using a 20× objective.

### Analysis of RGC survival rate.

To quantify RGC survival rate, mice were transcardially perfused 2 weeks after ONC or 1 week after NMDA injection and both retinas of each mouse were dissected. Retinas were stained with guinea pig anti-Rbpms (1:100, Sigma-Aldrich ABN1376) following the steps described above (see Immunofluorescence of whole-mount retinas). Randomly, 6–9 fields were taken from the peripheral regions of each retina. For each mouse, RGC survival rate was calculated by dividing the average number of Rbpms-positive cells in 1 field in the injured retina by that in the uninjured retina. Only cells in the ganglion cell layer were counted.

### Analysis of RGC transduction efficiency.

To quantify RGC transduction efficiency, mice were transcardially perfused 2 weeks after intravitreal injection of AAV2-*shSlc6a13-EGFP*. Retinas were stained with guinea pig anti-Rbpms (1:100, PhosphoSolutions 1832-RBPMS) and chicken anti-GFP (1:100, Thermo Fisher Scientific A10262) following the steps described above (see Immunofluorescence of whole-mount retinas). Eight fields were randomly taken from the peripheral regions of each retina. For each mouse, RGC transduction efficiency was calculated by dividing the total number of GFP and Rbpms double-positive cells in all fields by the total number of Rbpms-positive cells in all fields. Only cells in the ganglion cell layer were counted.

### Immunofluorescence of retinal sections.

Fixed retinas were immersed in 30% sucrose overnight at 4°C. On the next day, retinas were embedded in OCT compound, frozen, and cut into 10 μm sections with a cryostat. Sections were transferred onto microscope slides and warmed on a slide warmer for 1 hour at 37°C. Sections on slides were rinsed once in PBS, soaked in 100°C citrate buffer (pH 6) for 15 minutes, cooled down in the buffer to room temperature, washed 2 times for 5 minutes each time with PBS, and blocked with PBST (0.3%) containing 10% goat serum for 1 hour at room temperature. After blocking, sections were incubated in primary antibodies against target molecules overnight at 4°C, washed 4 times (5, 5, 10, and 10 minutes) with PBST (0.3%), incubated in corresponding Alexa Fluor–conjugated secondary antibodies (1:500, Thermo Fisher Scientific) for 1 hour at room temperature, and washed 4 times (5, 5, 10, and 10 minutes) again with PBST (0.3%). All antibodies were diluted with PBST (0.3%) containing 10% goat serum. Sections were mounted in Fluoroshield (Sigma-Aldrich) and covered by coverslips. Fluorescent images of retinal sections were obtained with a Zeiss inverted fluorescence microscope controlled by the AxioVision software using a 20× objective.

### Analysis of H3K27me3 levels in RGCs.

To analyze H3K27me3 levels in RGCs, retinas were dissected from transcardially perfused mice 2 weeks after intravitreal injection of AAV2-*GFP*, AAV2-*Ezh2*, or AAV2-*Ezh2-Y726D* and sectioned with a cryostat. Retinal sections were stained with guinea pig anti-Rbpms (1:500, Sigma-Aldrich ABN1376) and mouse anti-H3K27me3 (1:100, Sigma-Aldrich 05-1951) following the steps described above (see Immunofluorescence of retinal sections).

To quantify H3K27me3 levels in RGCs, fluorescence intensity of H3K27me3 immunoreactivity of at least 150 Rbpms-positive cells from 10–12 nonadjacent retinal sections acquired with identical imaging configurations was analyzed for each retina. Fluorescence intensity was measured using the “outline spline” function of the AxioVision software and the background fluorescence intensity was subtracted.

### Statistics.

Statistical analysis was done with GraphPad Prism 10 and the significance level was set as *P* 0.05. Data represent mean ± SEM unless otherwise stated. For comparisons between 2 conditions, 2-tailed unpaired or paired *t* test was used. For comparisons among 3 or more conditions, 1-way ANOVA followed by Tukey’s multiple comparisons was used to determine the statistical significance. All details of statistics, including tests used, *P* values, and sample sizes, are described in figure legends. *P* values of posthoc analyses are illustrated in figures. A *P* value less than 0.05 was considered significant.

### Study approval.

Protocols for animal experiments in this study were approved by the Animal Care and Use Committee of Johns Hopkins University.

### Data availability.

Raw and processed sequencing data are available in the Gene Expression Omnibus (GSE247320). Values for all data points in graphs are reported in the Supporting Data Values file.

## Author contributions

XWW, CML, and FQZ conceived the study and designed the project; XWW, SGY, and RYW performed the experiments. MWH, XL, and JQ analyzed the sequencing data. XWW and CZ performed data analysis. JJJ, ARK, and AJO helped with data analysis. YG helped with manuscript revision. XWW and FQZ wrote the manuscript with contributions from all authors. The order among cofirst authors was assigned based on contributions.

## Supplementary Material

Supplemental data

Unedited blot and gel images

Supplemental table 1

Supplemental table 2

Supplemental table 3

Supplemental table 4

Supporting data values

## Version 1 11/28/2023

In-Press Preview

## Version 2 02/01/2024

Electronic publication
