# Supplementary material for: Histone methyltransferase Ezh2 coordinates mammalian axon regeneration via regulation of key regenerative pathways
Source: J Clin Invest. 2024 Feb 1;134(3):e163145. doi: 10.1172/JCI163145 (PMC10849760; doi:10.1172/JCI163145)
Supplement: Supplemental data [file jci-134-163145-s010.pdf]

Supplemental Material for

Histone methyltransferase Ezh2 coordinates mammalian axon regeneration via regulation of key regenerative pathways

Xue-Wei Wang, Shu-Guang Yang, Ming-Wen Hu, Rui-Ying Wang, Chi Zhang, Anish R. Kosanam, Arinze J. Ochuba, Jing-Jing Jiang, Ximei Luo, Yun Guan, Jiang Qian, Chang-Mei Liu, and Feng-Quan Zhou

*Corresponding authors: Feng-Quan Zhou, Email: fzhou4@zju.edu.cn
Chang-Mei Liu, Email: liuchm@ioz.ac.cn
Xue-Wei Wang, Email: xueweiwang@usf.edu

This PDF file includes:

Supplemental Figure 1-10
Supplemental Table 5
Supplemental Methods
Supplemental References

Other Supplemental information for this manuscript includes:
Supplemental Table 1-4

Supplemental Figure 1

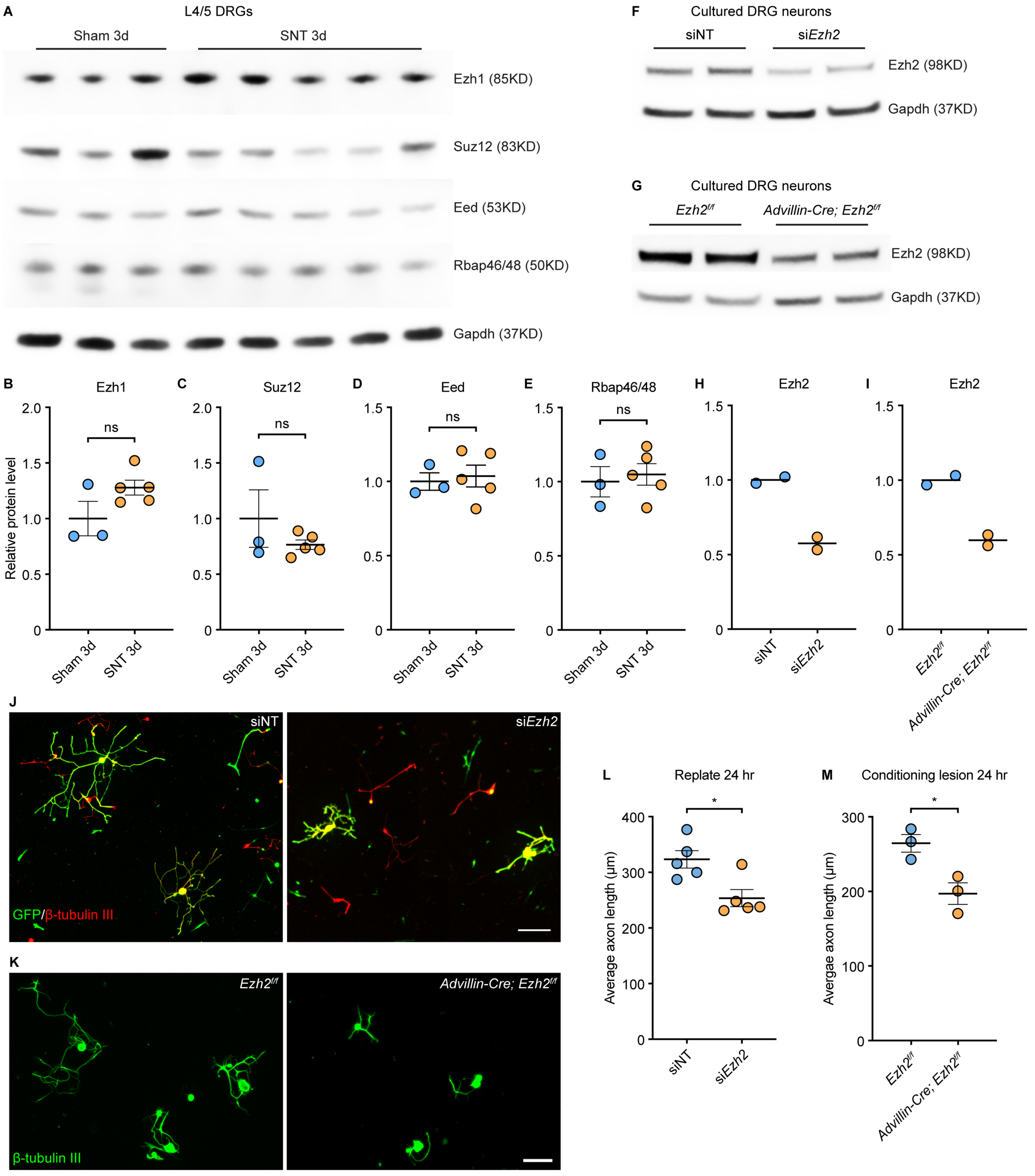

Supplemental Figure 1. *Ezh2* loss-of-function impairs regenerative axon growth of DRG neurons in vitro. (A) Immunoblotting showing that Ezh1, Suz12, Eed, and Rbap46/48 are not altered in lumbar 4 and 5 DRGs 3 days after sciatic nerve transection.

(B-E) Quantification of relative protein levels of Ezh1 (B), Suz12 (C), Eed (D), and Rbap46/48 (E) in (A) (two-tailed t test, unpaired; P = 0.1007 for Ezh1, P = 0.2791 for Suz12, P = 0.7412 for Eed, P = 0.6982 for Rbap46/48; n = 3 for sham, n = 5 for sciatic nerve transection).

(F) Immunoblotting showing decreased Ezh2 in cultured DRG neurons three days after electroporation of siRNAs targeting *Ezh2* mRNA.

(G) Immunoblotting showing decreased Ezh2 in cultured DRG neurons of *Advillin-Cre; Ezh2*$^{f/f}$ mice.

(H, I) Quantification of relative protein levels of Ezh2 in (F) and (G), respectively (n = 2 for all).

(J) Representative immunofluorescence of cultured DRG neurons showing that *Ezh2* knockdown impairs regenerative axon growth of sensory neurons in vitro. Cells were stained with anti-GFP (green) and anti-β-tubulin III (red). Scale bar, 200 μm.

(K) Representative immunofluorescence of cultured DRG neurons showing that *Ezh2* knockout impairs regenerative axon growth of conditioning-lesioned sensory neurons in vitro. Cells were stained with anti-β-tubulin III (green). Scale bar, 100 μm.

(L) Quantification of the average length of the longest axon of each neuron in (J) (two-tailed t test, unpaired; P = 0.0132; n = 5 independent experiments; at least 60 neurons were analyzed for each condition in each independent experiment, except in one experiment only 26 neurons were analyzed for *Ezh2* knockdown).

(M) Quantification of the average length of the longest axon of each neuron in (K) (two-tailed t test, unpaired; P = 0.0227; n = 3 independent experiments; at least 100 neurons were analyzed for each condition in each independent experiment).

L4/5, lumbar 4 and 5. SNT, sciatic nerve transection. siNT, non-targeting siRNAs. si*Ezh2*, siRNAs targeting *Ezh2* mRNA.

Supplemental Figure 2

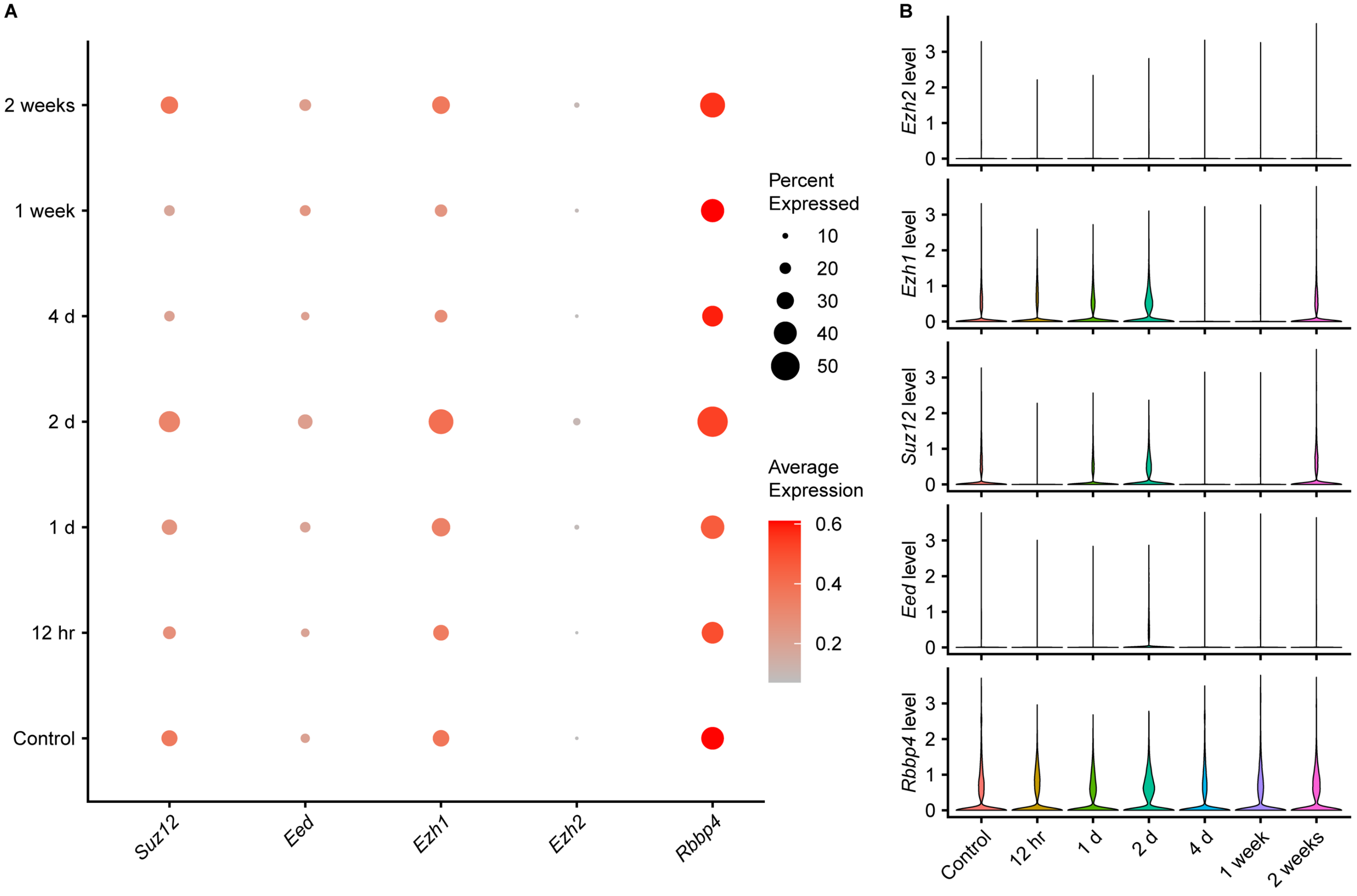

Supplemental Figure 2. Optic nerve crush does not significantly change mRNA levels of *Ezh2* or other PRC2 subunits in RGCs.

(A, B) Dot plot (A) and violin plots (B) showing that optic nerve crush does not significantly change mRNA levels of *Ezh2*, *Ezh1*, *Suz12*, *Eed*, or *Rbbp4* (encoding Rbap48).

Supplemental Figure 3

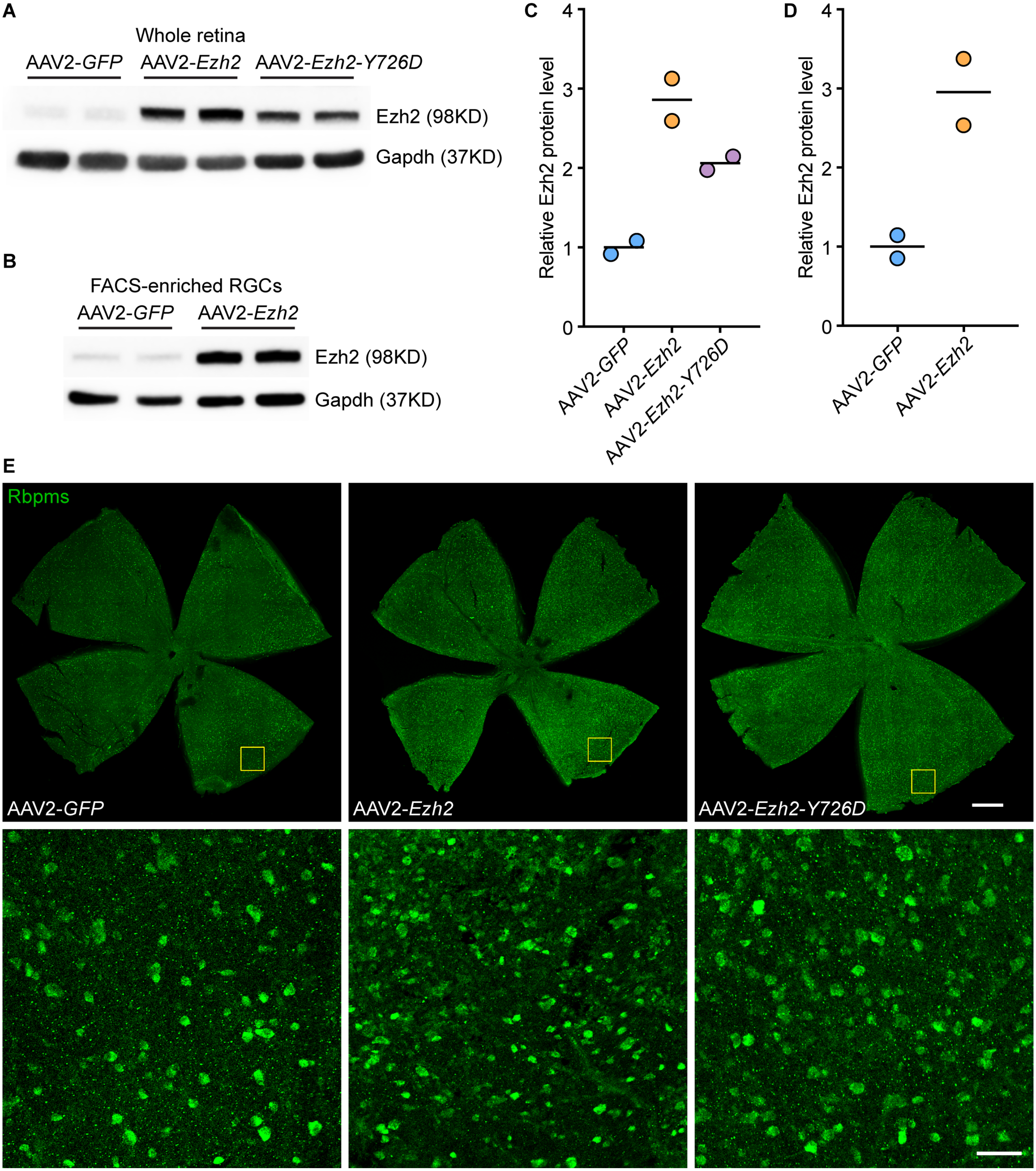

Supplemental Figure 3. *Ezh2* overexpression enhances RGC survival after optic nerve crush.
(A, B) Immunoblotting showing increased Ezh2 in retinas (A) or FACS-enriched RGCs (B) two weeks after intravitreal injection of AAV2-*Ezh2* or AAV2-*Ezh2-Y726D*.

(C, D) Quantification of relative protein levels of Ezh2 in (A) and (B), respectively (n = 2 for all).

(E) Immunofluorescence of whole-mount retinas showing that overexpression of *Ezh2* or *Ezh2-Y726D* improves RGC survival two weeks after optic nerve crush. Whole-mount retinas were stained with anti-Rbpms (green). The lower row displays enlarged images of the areas in yellow boxes in the upper row. Scale bar, 500 μm for the upper row, 50 μm for the lower row.

Supplemental Figure 4

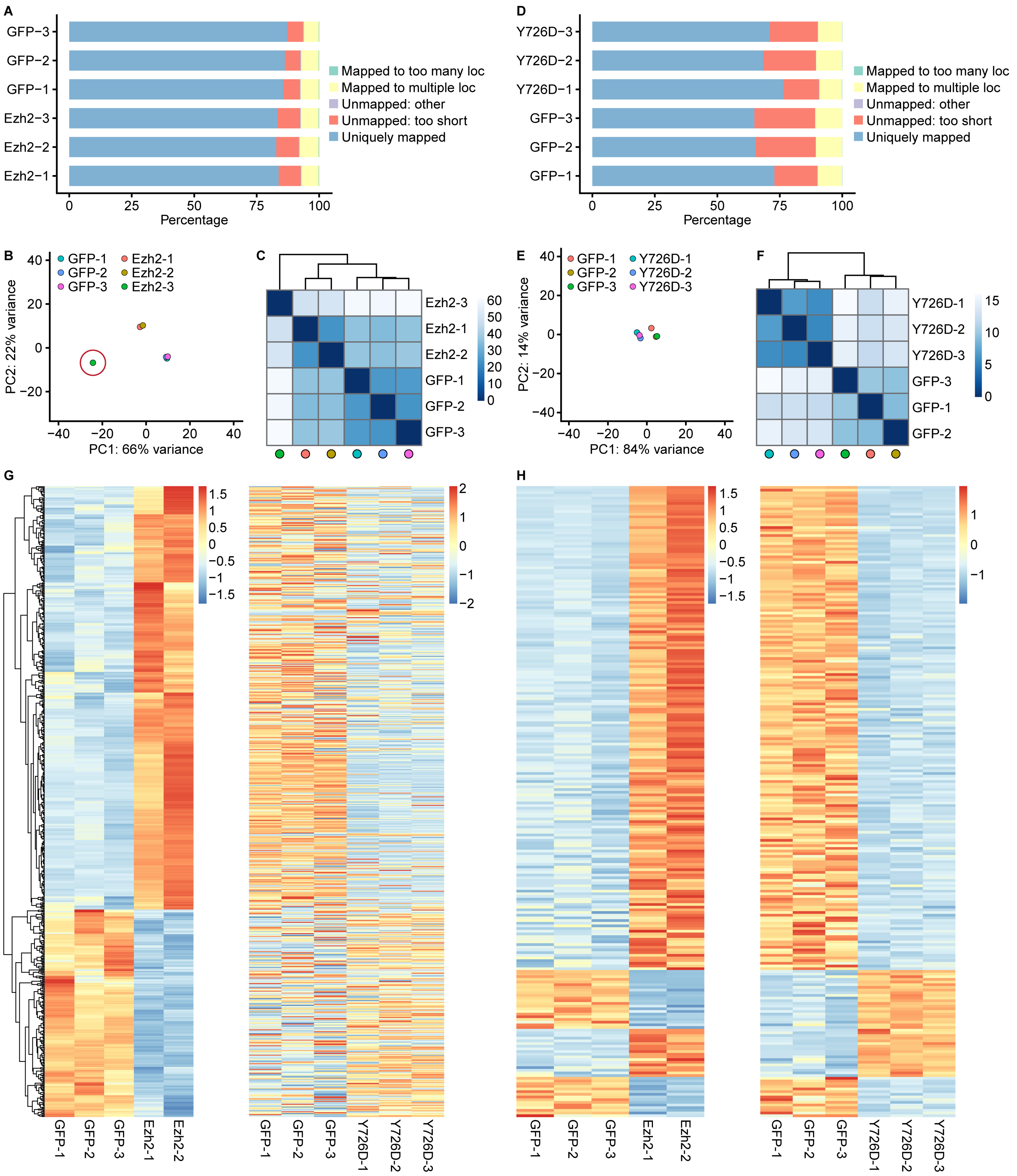

Supplemental Figure 4. Ezh2-Y726D acts as a dominant-negative form of Ezh2 in RGCs.
(A-C) Alignment score (A), principal component analysis (B), and hierarchical clustering (C) of libraries in the control vs. *Ezh2* overexpression RNA-seq. Note that one library in the *Ezh2* overexpression condition (circled in B) was excluded from further analysis due to low repeatability with other two libraries.

(D-F) Alignment score (D), principal component analysis (E), and hierarchical clustering (F) in the control vs. *Ezh2-Y726D* overexpression RNA-seq. Note that the control (GFP) libraries in (D-F) are independent of those in (A-C).

(G) Heatmaps of the 669 differentially expressed genes (DEGs) regulated by *Ezh2* overexpression in the control vs. *Ezh2* overexpression RNA-seq (left) or the control vs. *Ezh2-Y726D* overexpression RNA-seq (right). The same gene is represented in the same row on the left and right.

(H) Heatmaps of the 236 common DEGs regulated by both *Ezh2* overexpression and *Ezh2-Y726D* overexpression in the control vs. *Ezh2* overexpression RNA-seq (left) or the control vs. *Ezh2-Y726D* overexpression RNA-seq (right). The same gene is represented in the same row on the left and right.

Supplemental Figure 5

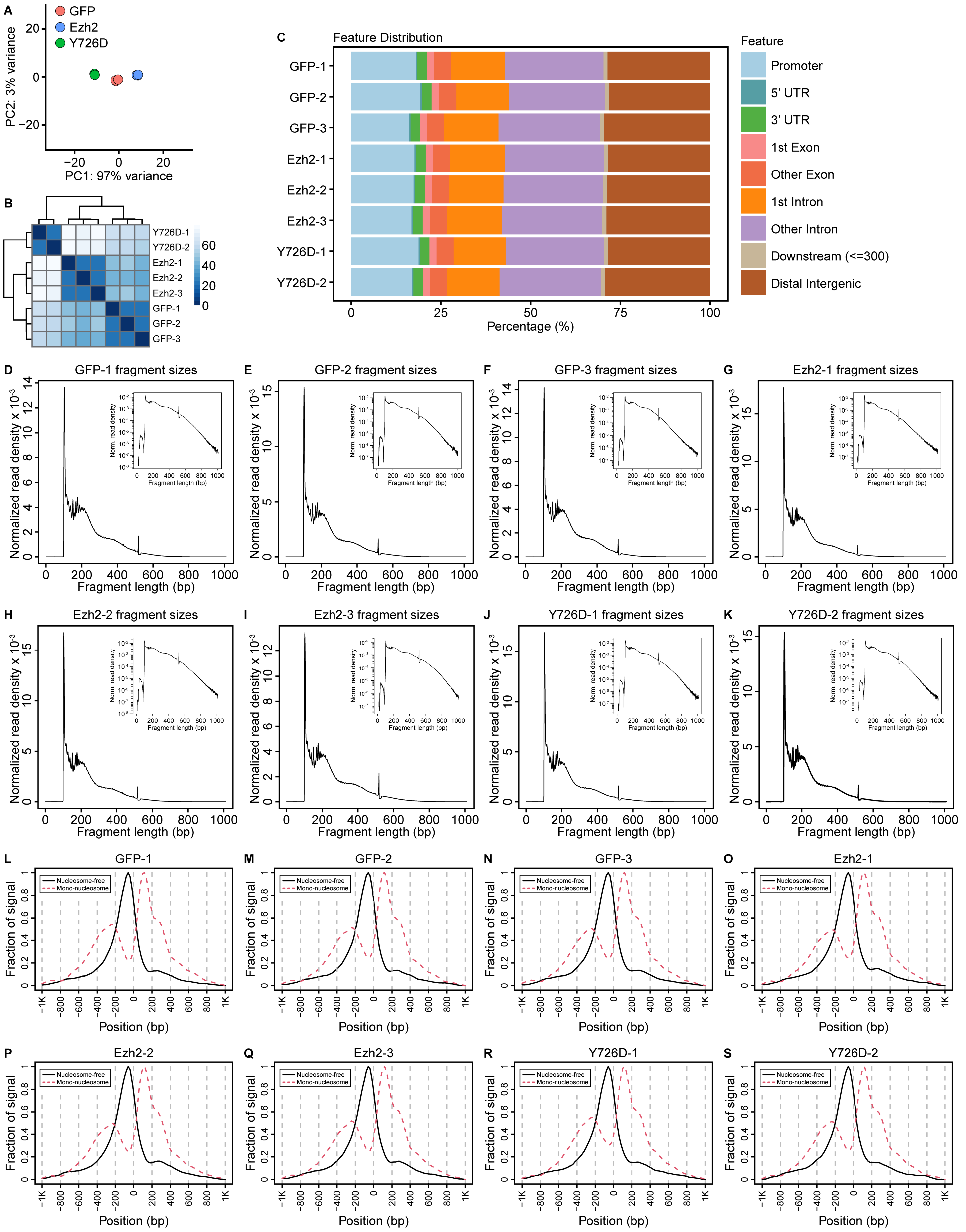

Supplemental Figure 5. Quality control of ATAC-seq.
(A-C) Principal component analysis (A), hierarchical clustering (B), and genomic feature distribution (C) of ATAC-seq libraries from control, *Ezh2* overexpression, and *Ezh2-Y726D* overexpression conditions.

(D-K) Fragment size distribution of ATAC-seq libraries showing typical enrichment around 100 and 200 bp, indicating nucleosome-free and mono-nucleosome-bound fragments, respectively.

(L-S) Transcription start site (TSS) enrichment of ATAC-seq libraries showing that nucleosome-free fragments (black) are enriched at TSS, whereas mono-nucleosome fragments (red dashed) are depleted at TSS but enriched at flanking regions.

Supplemental Figure 6

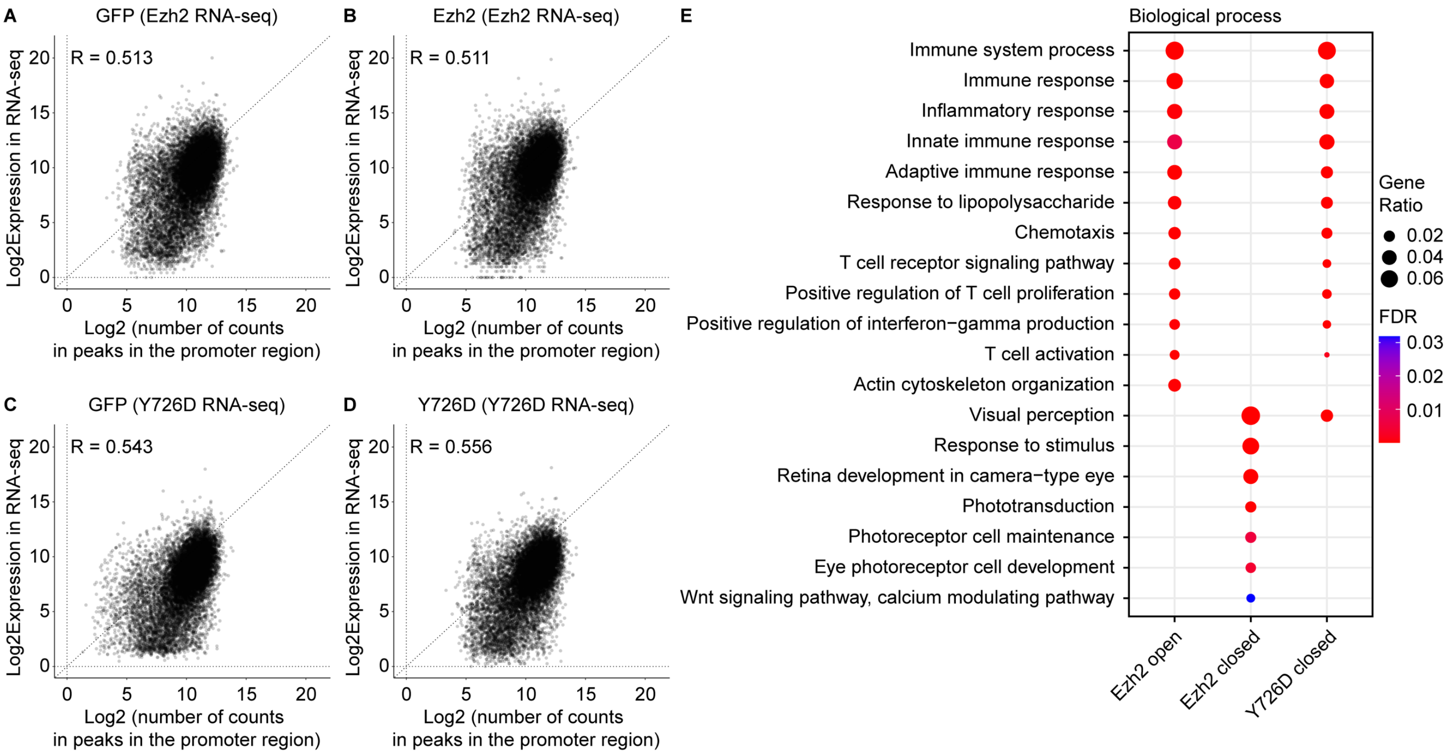

Supplemental Figure 6. ATAC-seq results are consistent with RNA-seq results.
(A-D) Pearson correlation between RNA expression in RNA-seq and chromatin accessibility at the promoter region in ATAC-seq within each condition.

(E) Gene ontology (GO) analysis of genes whose promoter regions became differentially accessible after *Ezh2* or *Ezh2-Y726D* overexpression. A subset of most significantly enriched GO terms in the biological process category are shown here.

Supplemental Figure 7

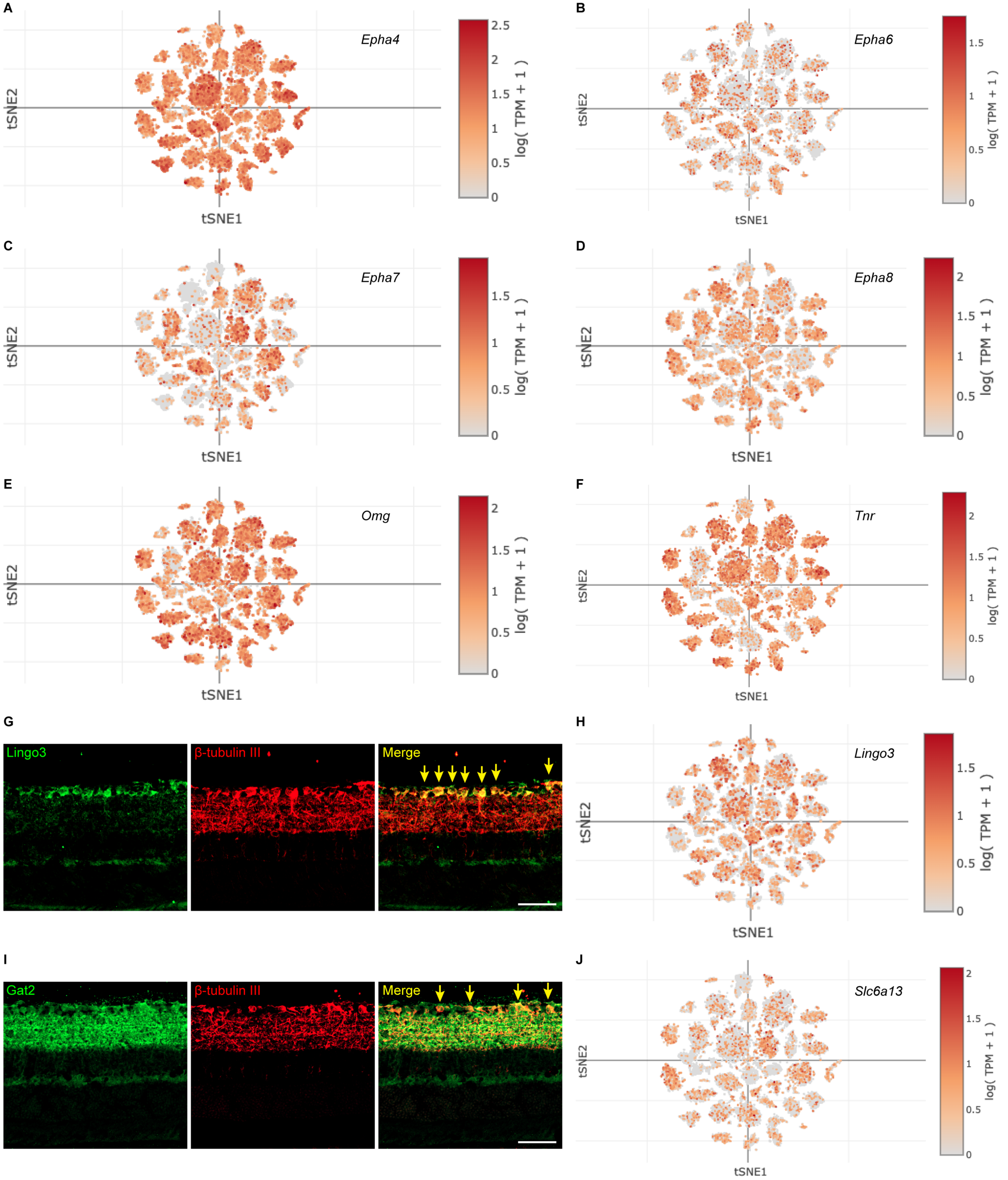

Supplemental Figure 7. Expression of a subset of genes transcriptionally suppressed by Ezh2 in RGCs. (A-F) Expression of *Epha4* (A), *Epha6* (B), *Epha7* (C), *Epha8* (D), *Omg* (E), and *Tnr* (F) mRNA in RGCs shown by t-SNE plots of RGC single-cell RNA-seq.

(G-J) Expression of Lingo3 (G, H) and Gat2 (encoded by *Slc6a13*) (I, J) protein and mRNA in RGCs shown by immunofluorescence of retinal sections and t-SNE plots of RGC single-cell RNA-seq. Yellow arrows indicate RGCs expressing Lingo3 (G) or Gat2 (I). Scale bar, 50 μm.

t-SNE plots are from https://singlecell.broadinstitute.org/single_cell/study/SCP509/mouse-retinal-ganglion-cell-adult-atlas-and-optic-nerve-crush-time-series (1).

Supplemental Figure 8

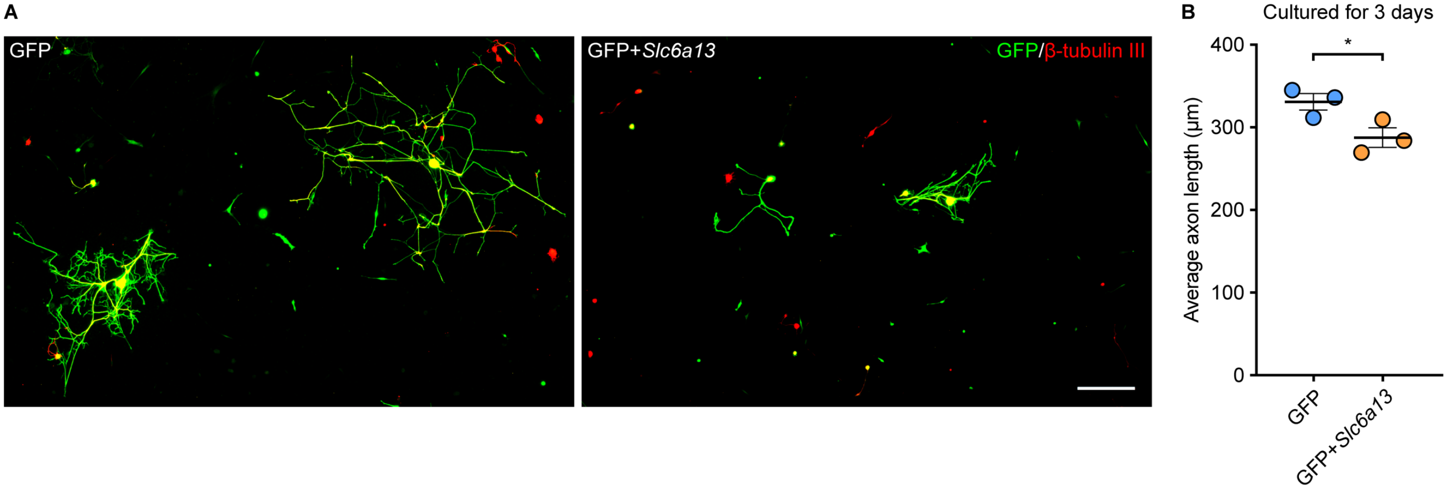

Supplemental Figure 8. *Slc6a13* overexpression impairs regenerative axon growth of DRG neurons in vitro.
(A) Representative immunofluorescence of cultured DRG neurons showing that *Slc6a13* overexpression impairs regenerative axon growth of DRG neurons in vitro. Cells were stained with anti-GFP (green) and anti-β-tubulin III (red). Scale bar, 100 μm.

(B) Quantification of the average length of the longest axon of each neuron in (A) (two-tailed t test, unpaired; P = 0.0481; n = 3 independent experiments; at least 60 neurons were analyzed for each condition in each independent experiment).

Supplemental Figure 9

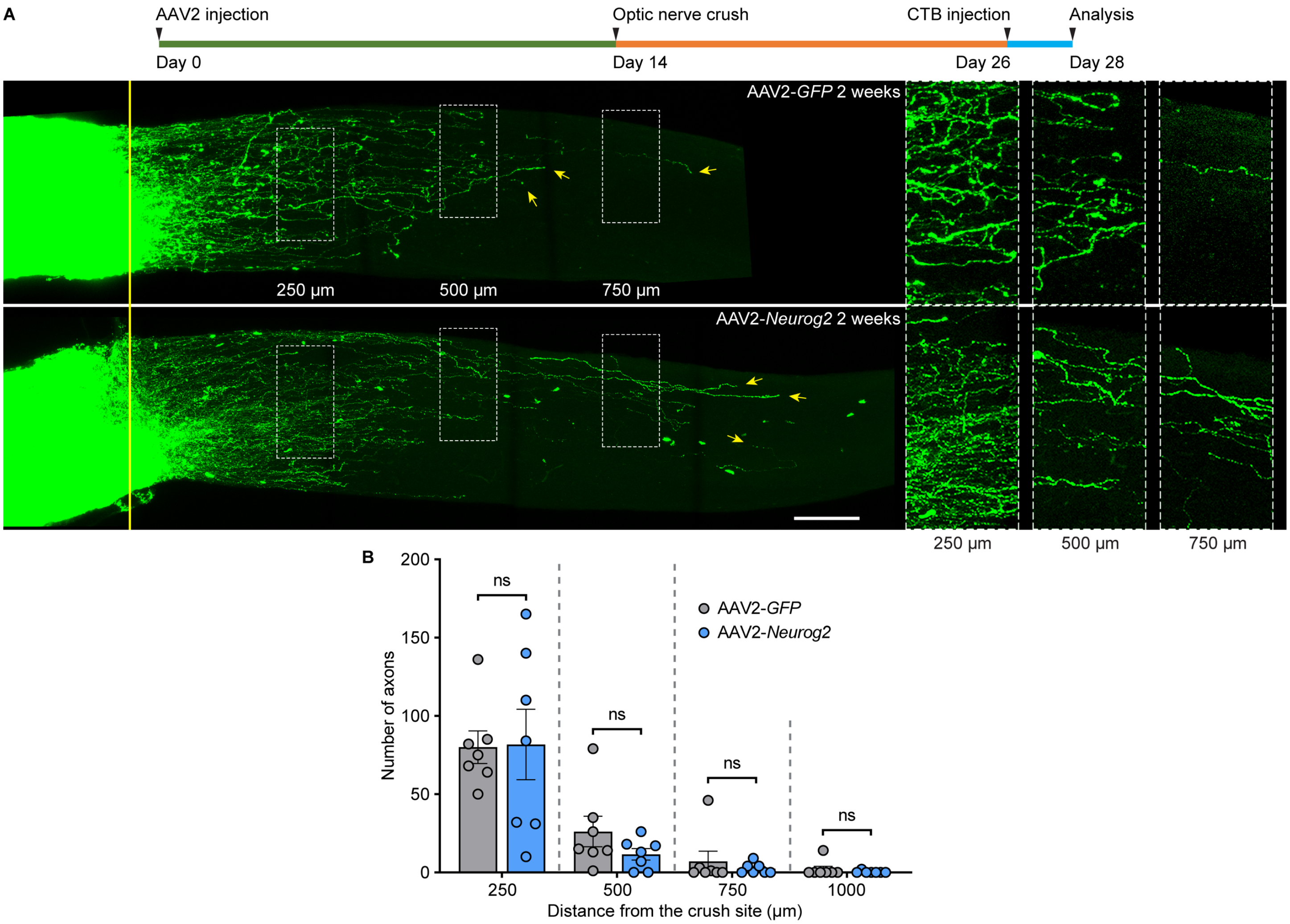

Supplemental Figure 9. *Neurog2* overexpression does not promote optic nerve regeneration.
(A) Top: Experimental timeline. Bottom: Representative images of optic nerves showing that *Neurog2* overexpression does not promote optic nerve regeneration. Columns on the right display enlarged images of the areas in white, dashed boxes on the left, showing axons at 250, 500, and 750 µm distal to the crush sites, which are aligned with the yellow line. Yellow arrows indicate longest axons in each nerve. Scale bar, 100 µm (50 µm for enlarged images).

(B) Quantification of optic nerve regeneration in (A) (two-tailed t test, unpaired; P = 0.9460 at 250 µm, P = 0.1854 at 500 µm, P = 0.4900 at 750 µm, P = 0.4128 at 1,000 µm; n = 7 mice for both).

Supplemental Figure 10

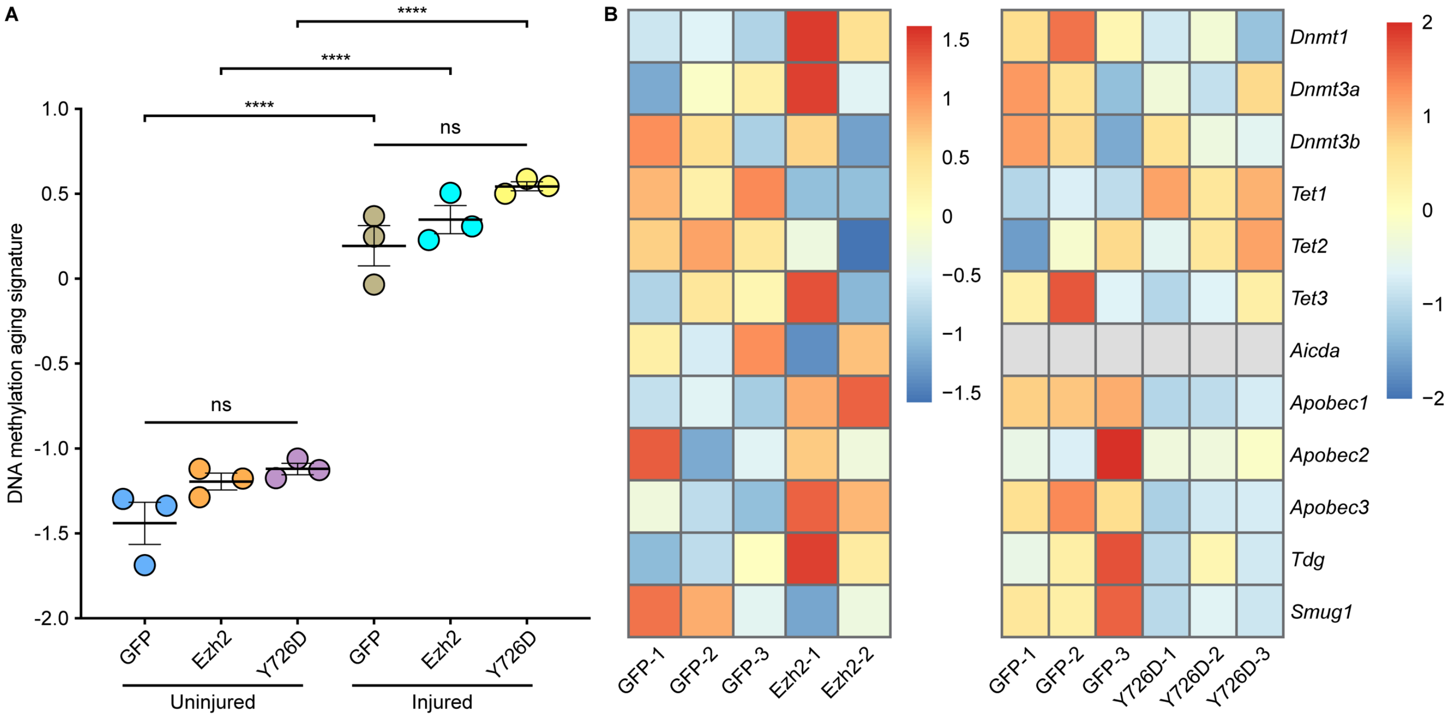

Supplemental Figure 10. *Ezh2* overexpression does not alter the epigenetic aging clock of RGCs. (A) DNA methylation aging signature of RGCs is increased by optic nerve injury, but not affected by *Ezh2* or *Ezh2-Y726D* overexpression (one-way ANOVA followed by Tukey's multiple comparisons; $P < 0.0001$; $n = 3$ reduced representation bisulfite sequencing libraries for all).

(B) Heatmaps of mRNA levels of 5mC DNA methyltransferases and demethylases showing that most of them are not significantly changed by *Ezh2* or *Ezh2-Y726D* overexpression in RGCs. Note that *Aicda* mRNA was not detected in the control vs. *Ezh2-Y726D* overexpression RNA-seq.

Supplemental Table 5. Target sequences of siRNAs and sequences of primers.

siRNA or primer	Sequence
Ezh2 siRNAs (pool of 4)	GCACAAGUCAUCCCGUUAA
	CAGAGAAUGUGGAUUUAUA
	GGGAUGAAGUUCUGGAUCA
	GGUAAAUGCUCUUGGUCAA
Non-targeting siRNAs (pool of 4)	UGGUUUACAUGUCGACUAA
	UGGUUUACAUGUUGUGUGA
	UGGUUUACAUGUUUUCUGA
	UGGUUUACAUGUUUUCCUA
Slc6a13 promoter region 1 forward primer	GTGGGCCGGGTCAAGTTTAG
Slc6a13 promoter region 1 reverse primer	GGCTGGCTATTTAAAGCGTGG
Slc6a13 promoter region 2 forward primer	GTAGAATCACAAGCAACCGGC
Slc6a13 promoter region 2 reverse primer	AGACAATCCGCACATGAGTGA
Lingo3 promoter region forward primer	GGCTCTGCCAAGTCCTAGTG
Lingo3 promoter region reverse primer	CGGGTGGCAACTGGAGTAAG
Omg promoter region 1 forward primer	TCGTCTTGTTGTGGGAGTCG
Omg promoter region 1 reverse primer	TCACAGCAACACAATGCAGC
Omg promoter region 2 forward primer	GCTGCATTGTGTTGCTGTGA
Omg promoter region 2 reverse primer	GTTGCGTATGCACCTCTTGC
Wfdc1 promoter region 1 forward primer	TGCCCAGCTTACAGTCACTC
Wfdc1 promoter region 1 reverse primer	CCCTGGGTAGTGGTTGGTTC
Wfdc1 promoter region 2 forward primer	TGTCCCCTCCCTATAGCACC
Wfdc1 promoter region 2 reverse primer	AGTTAGGATGGGCTGTGCTG

Supplemental Methods

Constructs. The mouse *Ezh2* open reading frame (ORF) was cloned into pAAV-*CMV* with 5' NheI and 3' XhoI restriction sites to obtain pAAV-*CMV-Ezh2*. pAAV-*CMV-Ezh2-Y726D* was constructed by mutating the 2,176th nucleotide of *Ezh2* ORF from a T to a G. The mouse *Slc6a13* ORF with 5' NheI and 3' NotI restriction sites was cloned into pAAV-*CMV* to obtain pAAV-*CMV-Slc6a13* or used to replace the *EGFP* ORF in p*CMV-EGFP* to obtain p*CMV-Slc6a13*. Mouse *Lingo3*, *Omg*, and *Wfdc1* ORF's were cloned into pAAV-*CMV* using 5' KpnI and 3' ApaI restriction sites to obtain pAAV-*CMV-Lingo3*, pAAV-*CMV-Omg*, and pAAV-*CMV-Wfdc1*, respectively. The mouse *Neurog2* ORF flanked by 5' BamHI and 3' EcoRV restriction sites was used to replace the *EYFP* ORF in pAAV-*Ef1a-EYFP* to obtain pAAV-*Ef1a-Neurog2*. Mouse *Lingo3* and *Neurog2* ORF's were synthesized by Integrated DNA Technologies with codon optimization. pAAV-*U6-shSlc6a13-CMV-EGFP* or pAAV-*U6-shLingo3-CMV-EGFP* was constructed by inserting CCGAATCAATAACATCCCATTTTCAAGAGAAATGGGATGTTATTGATTCGGTTTTTT or CCGCGGACAGCACAAGAATAATTCAAGAGATTATTCTTGTGCTGTCCGCGGTTTTTT into pAAV-*U6-CMV-EGFP* using 5' BamHI and 3' HindIII restriction sites. Enzymes used for molecular cloning in this study include NheI-HF (R3131), XhoI (R0146), NotI-HF (R3189), KpnI (R3142), ApaI (R0114), BamHI-HF (R3136), EcoRV-HF (R3195), HindIII-HF (R3104), and T4 DNA ligase (M0202) and were all purchased from New England Biolabs. Plasmid vectors were amplified using DH5α competent cells (Thermo Fisher Scientific 18258012) and purified with the Endofree Plasmid Maxi Kit (Qiagen 12362) for electroporation or virus packaging. AAV2-*GFP* (SL100812) and AAV2-*shScramble-GFP* (SL100815) were purchased from SignaGen Laboratories. All other AAV2 viruses were also packaged by SignaGen Laboratories.

Adult DRG neuronal culture. Lumbar DRGs were dissected from euthanized 6-8-week-old mice, digested with 1 mg/ml type I collagenase (Thermo Fisher Scientific 17100017) and 5 mg/ml dispase II (Thermo Fisher Scientific 17105041) at 37°C for 70 min, washed 3 times with HBSS, and dissociated into cell suspension by trituration in MEM containing 10% fetal bovine serum (FBS) and 1% penicillin/streptomycin (Thermo Fisher Scientific 10378016). Cells were filtered with a 100-μm cell strainer and pelleted by centrifugation (500 g, 5 min, room temperature).

For electroporation experiments, pelleted cells were resuspended with 100 μl nucleofection buffer (Mouse Neuron Nucleofector Kit, Lonza VPG-1001) containing 0.2 nmol siRNAs (siNT*,* Horizon Discovery *D-001810-10-05* or si*Ezh2*, Horizon Discovery L-040882-00-0005, see Table S5 for target sequences) and/or 10 μg plasmid vectors and electroporated with Nucleofector II (Lonza). Cells were then immediately resuspended in pre-warmed (37°C) MEM containing 5% FBS, 1% GlutaMAX-I (Thermo Fisher Scientific 35050061), 1% penicillin/streptomycin and antimitotic reagents (20 μM 5-Fluoro-2'-deoxyuridine, MilliporeSigma F0503 and 20 μM uridine, MilliporeSigma U3750), plated on coverslips pre-coated with 100 μg/ml poly-D-lysine (MilliporeSigma P6407) and 10 μg/ml laminin (Thermo Fisher Scientific 23017015), and cultured for 3 days at 37°C in a humidified incubator containing 5% CO_2. Culture medium was refreshed 6 hours after plating.

For replate experiments, electroporated cells were plated on pre-coated dishes and cultured for 3 days. Cells were then forced to detach from dishes by pipetting, replated on pre-coated coverslips, and cultured for 24 hours.

For culture of conditioning lesioned DRG neurons, L4/5 DRGs were dissected from *Ezh2*$^{f/f}$ and *Advillin-Cre; Ezh2*$^{f/f}$ mice 3 days after sciatic nerve transection (see ***Sciatic nerve crush or transection*** in Methods). After enzymatic digestion and dissociation, filtered cells were immediately plated on pre-coated coverslips and cultured for 24 hours.

Immunofluorescence of cultured cells. Cultured DRG cells were fixed with 4% PFA for 15 min at room temperature, washed 3 times with PBS, and blocked with PBST (0.3%) containing 10% goat serum for 1 hour at room temperature. After blocking, cells were incubated in mouse anti-β-tubulin III (1:1,000, BioLegend 801202) and/or chicken anti-GFP (1:1,000, Thermo Fisher Scientific A10262) for 1 hour at room temperature, washed 3×10 min with PBS, incubated in corresponding Alexa Fluor-conjugated secondary antibodies (1:500, Thermo Fisher Scientific) for 1 hour at room temperature, and washed 3×10 min again with PBS. All antibodies were diluted with PBST (0.3%) containing 10% goat serum. Coverslips were then mounted in Fluoroshield (MilliporeSigma F6182) onto microscope slides.

Analysis of in vitro DRG neuron axon growth. Fluorescent images of cultured DRG neurons were obtained with a Zeiss inverted fluorescence microscope controlled by the AxioVision software using a 5× objective. The longest axon of each neuron was manually traced and measured with the built-in “measure/curve spline” function of the AxioVision software. Only neurons with axons longer than twice the diameter of their somas were included. In most experiments, at least 60 neurons were analyzed in each condition. Measurement was done by experimenters blinded to experimental conditions.

Tissue clearing of optic nerves. Two days after intravitreal CTB injection, mice were anesthetized and transcardially perfused with PBS followed by 4% PFA. Optic nerves were dissected and post-fixed in 4% PFA overnight at 4°C. On the next day, optic nerves were washed 3 times with PBS, dehydrated in an ascending series of tetrahydrofuran (50%, 70%, 80%, 100% and 100%, v/v in distilled water, 20 min each, MilliporeSigma 186562), and cleared in a 1:2 mixed solution of benzyl alcohol and benzyl benzoate (BABB, MilliporeSigma 305197 and B6630). Incubations were done on an orbital shaker at room temperature. Nerves were stored in BABB and protected from light at room temperature before imaging.

RNA-seq and data analysis. RNA was isolated from FACS-enriched RGCs 3 days after ONC using the PicoPure RNA Isolation Kit (Thermo Fisher Scientific KIT0204) following the manufacture’s manual. RNA quality was verified using the Agilent Fragment Analyzer (Agilent Technologies). RNA-seq libraries were prepared using the TruSeq Stranded Total RNA Kit (Illumina) and quality checked by the Agilent Fragment Analyzer. Equimolar amounts of the finished libraries were then pooled and sequenced on an Illumina NextSeq 500 with 2×75 bp paired reads.

Raw FASTQ data were mapped to the mouse reference genome (GRCm38) using STAR aligner (version 2.7.0d) with default parameters. The number of counts per gene was estimated using the “quantMode” command in STAR. Quantified raw counts were used in DESeq2 (version 1.22.2) to obtain DEGs. Genes with less than 10 counts in total from six libraries were excluded from analysis. Genes with adjusted $P < 0.05$ and fold change > 1.5 were chosen as DEGs. Principal component analysis (PCA) and hierarchical clustering were also performed with the transformed count matrix in DESeq2. One library in the *Ezh2* overexpression condition was excluded from further data analysis due to low repeatability with two other libraries in the same condition. Normalized counts were used to produce heatmaps and scatter plots. GO analysis (biological process) was done using DAVID Bioinformatics Resources 6.8.

ATAC-seq and data analysis. ATAC-seq libraries were constructed from FACS-enriched RGCs (50,000 cells for each library) 3 days after ONC following a previously published protocol (2). Briefly, cells were pelleted by centrifugation (500 g, 5 min, 4°C), washed with ice-cold PBS, pelleted again by centrifugation (500 g, 5 min, 4°C), and lysed in 50 µl ice-cold lysis buffer. Immediately after lysis, nuclei were pelleted by centrifugation (500 g, 10 min, 4°C), resuspended in 50 µl transposase reaction mix (Tagment DNA TDE1 Enzyme and Buffer Kits, Illumina 20034197) and incubated at 37°C for 30 min. After the transposition reaction, the product was purified with the DNA Clean & Concentrator-5 Kit (Zymo Research D4003). 20 µl tagmented DNA was PCR amplified with NEBNext High-Fidelity PCR Master mix (New England Biolabs M0541) and forward and reverse UDI primers. Amplification was first performed for 5 cycles, following which 5 µl of each partially amplified library was used to perform qPCR to determine the additional number of PCR cycles needed for each library. Final amplified libraries were purified using 1.1× Ampure XP bead purification (Beckman Coulter A63880). Equimolar amounts of the finished libraries were then pooled and sequenced on an Illumina NovaSeq 6000 with 2×100 bp paired reads.

After removing adapters of Illumina reads with Cutadapt, pair-end ATAC-seq reads were mapped to the mouse reference genome (GRCm38) using Bowtie2 with default parameters. Qualified properly paired reads (MAPQ score > 10) were assessed by SAMTools. Duplicate reads were removed with MarkDuplicates function in Picard. After using MACS2 to call peak regions of each sample, we used multiBamSummary function in deepTools to calculate read counts for all samples. ChIPseeker was used to annotate genomic context of identified peaks. Gene annotation information was accessed using TxDb.Mmusculus.UCSC.mm10.knownGene. Differential accessibility analysis was performed by DESeq2. GO analysis (biological process) was done using DAVID Bioinformatics Resources 6.8. Fragment size distribution and transcription start site enrichment were performed by ATACseqQC.

RRBS and data analysis. Three days after ONC (injured conditions) or 17 days after AAV2 injection (uninjured conditions), DNA was extracted from FACS-enriched RGCs using the Quick-DNA Microprep Plus Kit (Zymo Research D4074) following the manufacture's manual. RRBS library preparation and sequencing were done by Zymo Research. Briefly, 10 ng genomic DNA was digested with 30 units of MspI. Fragments were ligated to pre-annealed adapters containing 5'-methyl-cytosine instead of cytosine according to Illumina's specified guidelines. Adaptor-ligated fragments ≥ 50 bp in size were recovered using the DNA Clean & Concentrator-5 Kit (Zymo Research D4003). The fragments were then bisulfite-treated using the EZ DNA Methylation-Lightning Kit (Zymo Research D5030). Preparative-scale PCR was performed, and the product was purified with the DNA Clean & Concentrator-5 Kit (Zymo Research D4003) for sequencing on an Illumina NovaSeq with 2×150 bp paired reads.

Sequencing reads from bisulfite-treated classic RRBS libraries were identified using standard Illumina base calling software and then raw FASTQ files were adapter, filled-in nucleotides, and quality trimmed using TrimGalore 0.6.4. FastQC 0.11.8 was used to assess the effect of trimming and overall quality distributions of the data. Alignment to the mm10 reference genome was performed using Bismark 0.19.0. Methylated and unmethylated read totals for each CpG site were called using MethylDackel 0.5.0. The methylation level of each sampled cytosine was estimated as the number of reads reporting a C, divided by the total number of reads reporting a C or T.

DNA methylation aging signature was estimated by the method described in a previous study (3). 1-month, 12-month, and 18-month mouse samples in that study were used as the training set in the predictive PCA model. Differential CpG sites were selected based on top 50% CpGs using biweight midcorrelation among three comparisons (i.e., AAV2-*GFP* uninjured vs AAV2-*GFP* injured, AAV2-*Ezh2* uninjured vs AAV2-*Ezh2* injured, and AAV2-*Ezh2-Y726D* uninjured vs AAV2-*Ezh2-Y726D* injured). The first principal component was chosen to represent the DNA methylation aging signature.

Analysis of single-cell RNA-seq data of RGCs. The data used to analyze the time course of transcriptional changes of PRC2 components in RGCs following ONC was downloaded from Gene Expression Omnibus (GSE137398). Expression matrices were integrated by Seurat (v4.1.0) with default parameters.

Detection of Gat2 and Lingo3 expression in RGCs. To detect Gat2 and Lingo3 expression in RGCs, retinal sections were stained with mouse anti-β-tubulin III (1:500, BioLegend 801202) and rabbit anti-Gat2 (1:100, Abcam ab229815) or rabbit anti-Lingo3 (1:300, Abcam ab169772) following the steps described in ***Immunofluorescence of retinal sections*** in Methods.

Supplemental References

1. Tran NM, Shekhar K, Whitney IE, Jacobi A, Benhar I, Hong G, et al. Single-Cell Profiles of Retinal Ganglion Cells Differing in Resilience to Injury Reveal Neuroprotective Genes. *Neuron.* 2019;104(6):1039-55 e12.
2. Buenrostro JD, Wu B, Chang HY, and Greenleaf WJ. ATAC-seq: A Method for Assaying Chromatin Accessibility Genome-Wide. *Curr Protoc Mol Biol.* 2015;109:21 9 1- 9 9.
3. Lu Y, Brommer B, Tian X, Krishnan A, Meer M, Wang C, et al. Reprogramming to recover youthful epigenetic information and restore vision. *Nature.* 2020;588(7836):124-9.
