# Supplementary material for: Histone methyltransferase Ezh2 coordinates mammalian axon regeneration via regulation of key regenerative pathways
Source: J Clin Invest. 2024 Feb 1;134(3):e163145. doi: 10.1172/JCI163145 (PMC10849760; doi:10.1172/JCI163145)

Full unedited gel for Figure 1A, Ezh2

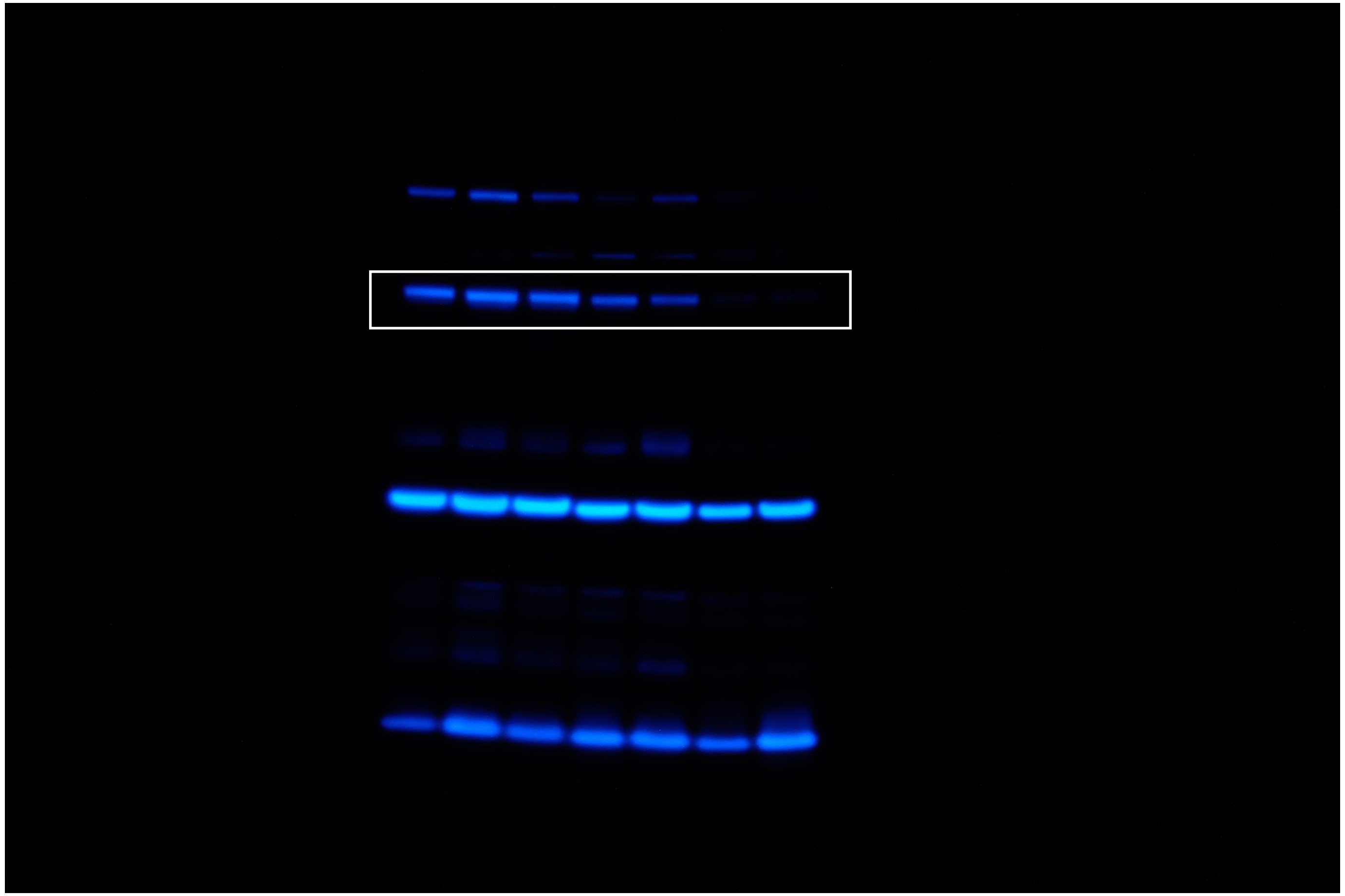

Full unedited gel for Figure 1A, β-actin

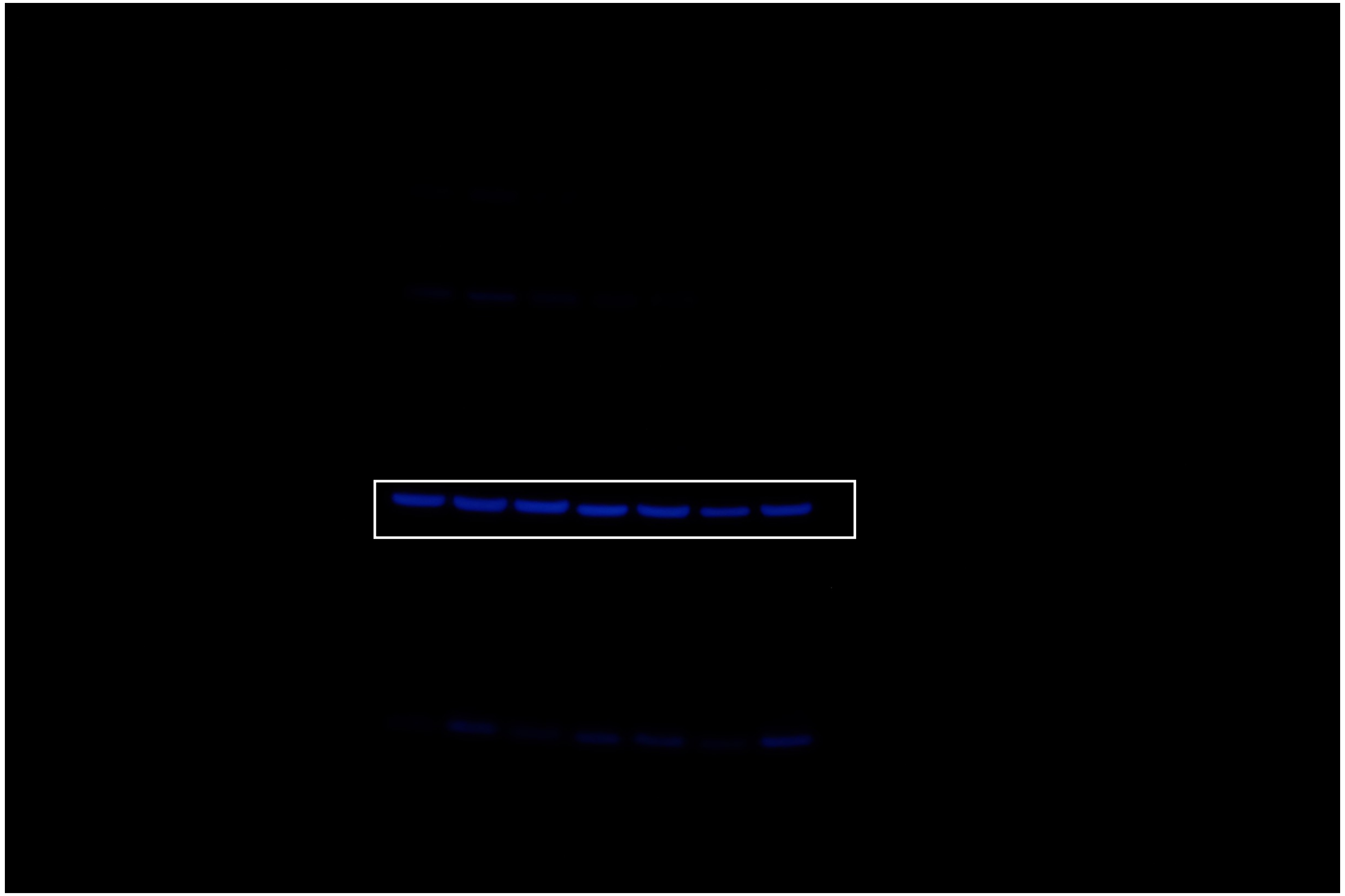

Full unedited gel for Figure 1B, Ezh2

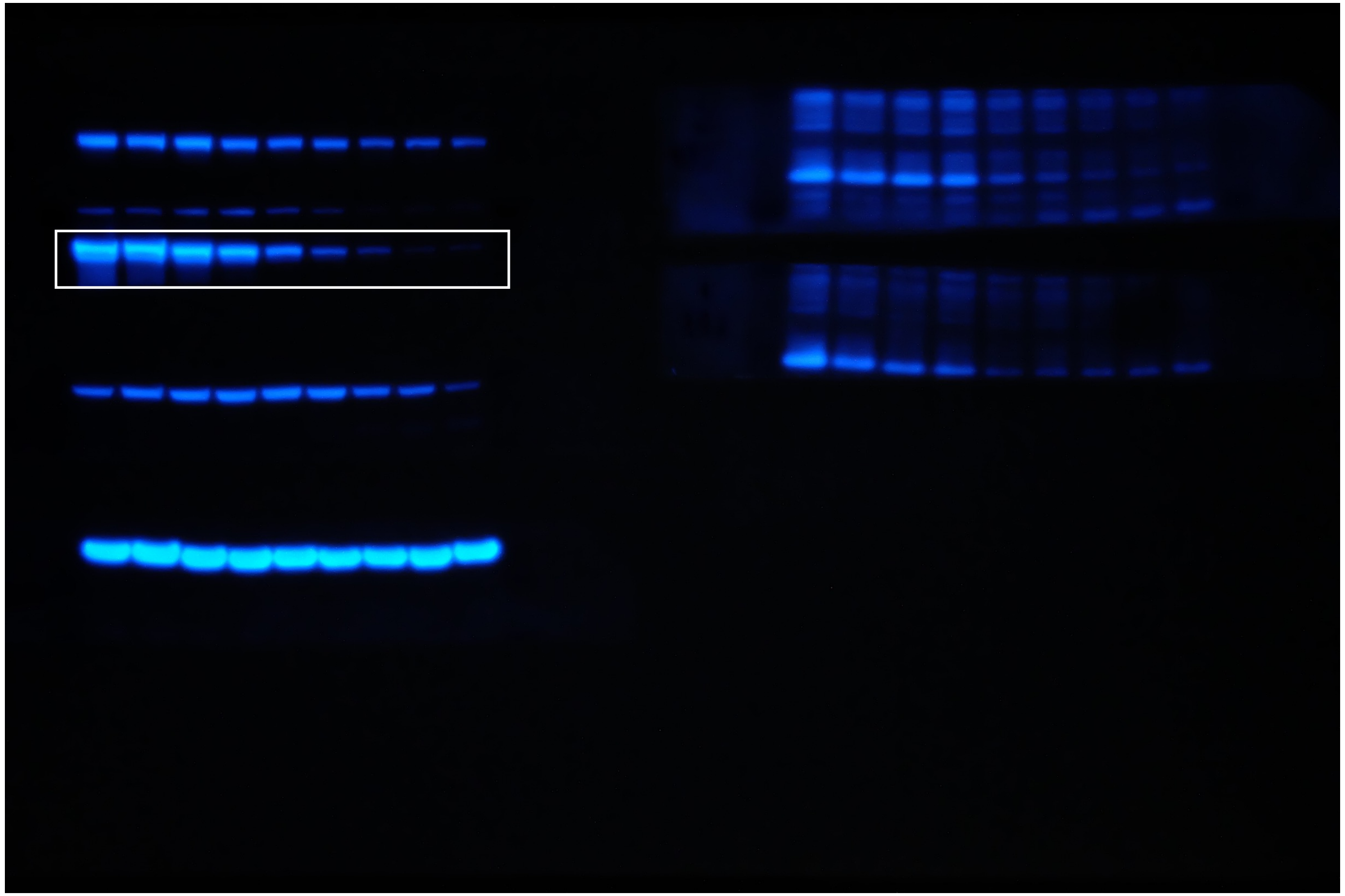

Full unedited gel for Figure 1B, β-actin

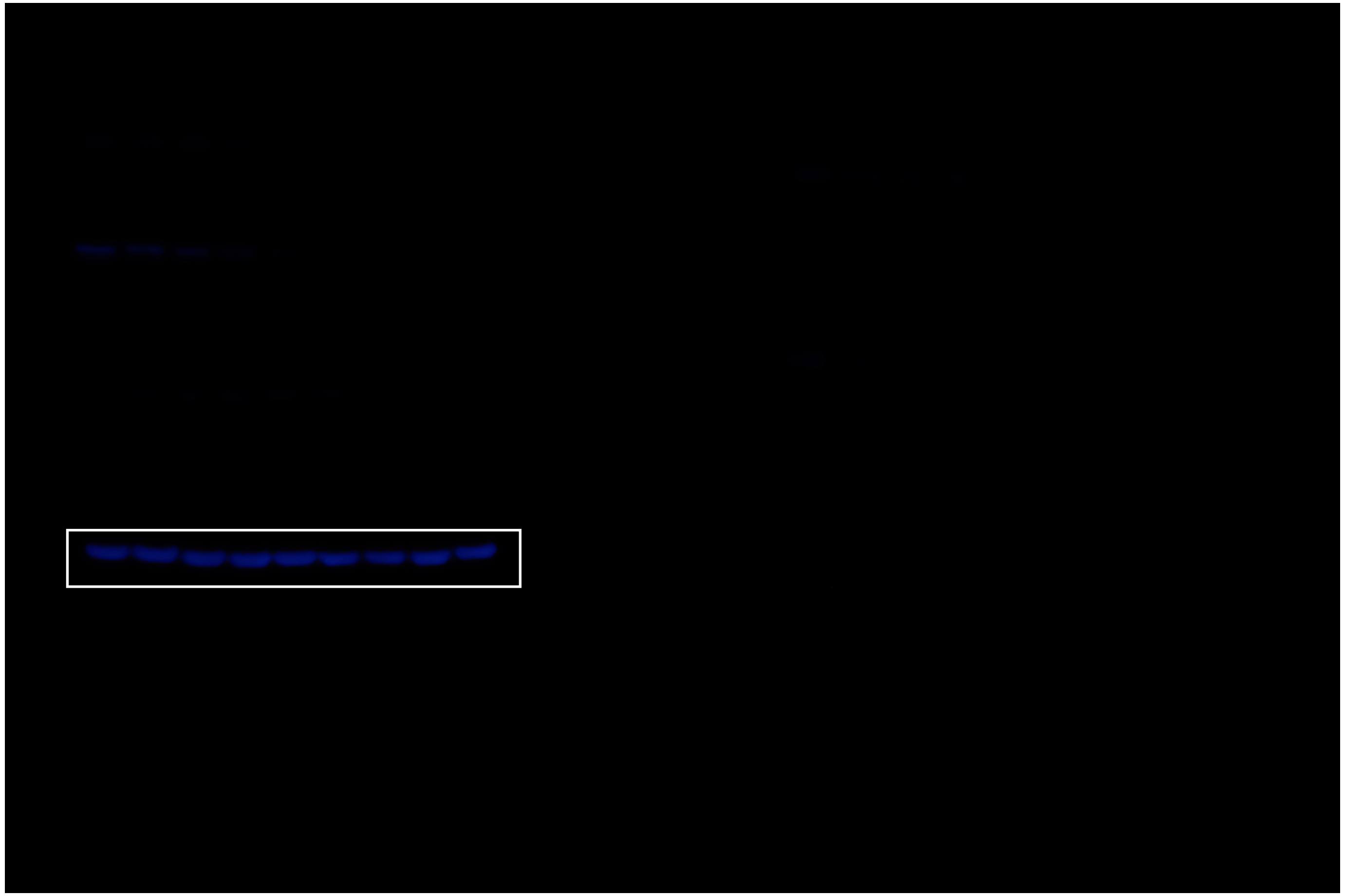

Full unedited gel for Figure 1C, Ezh2

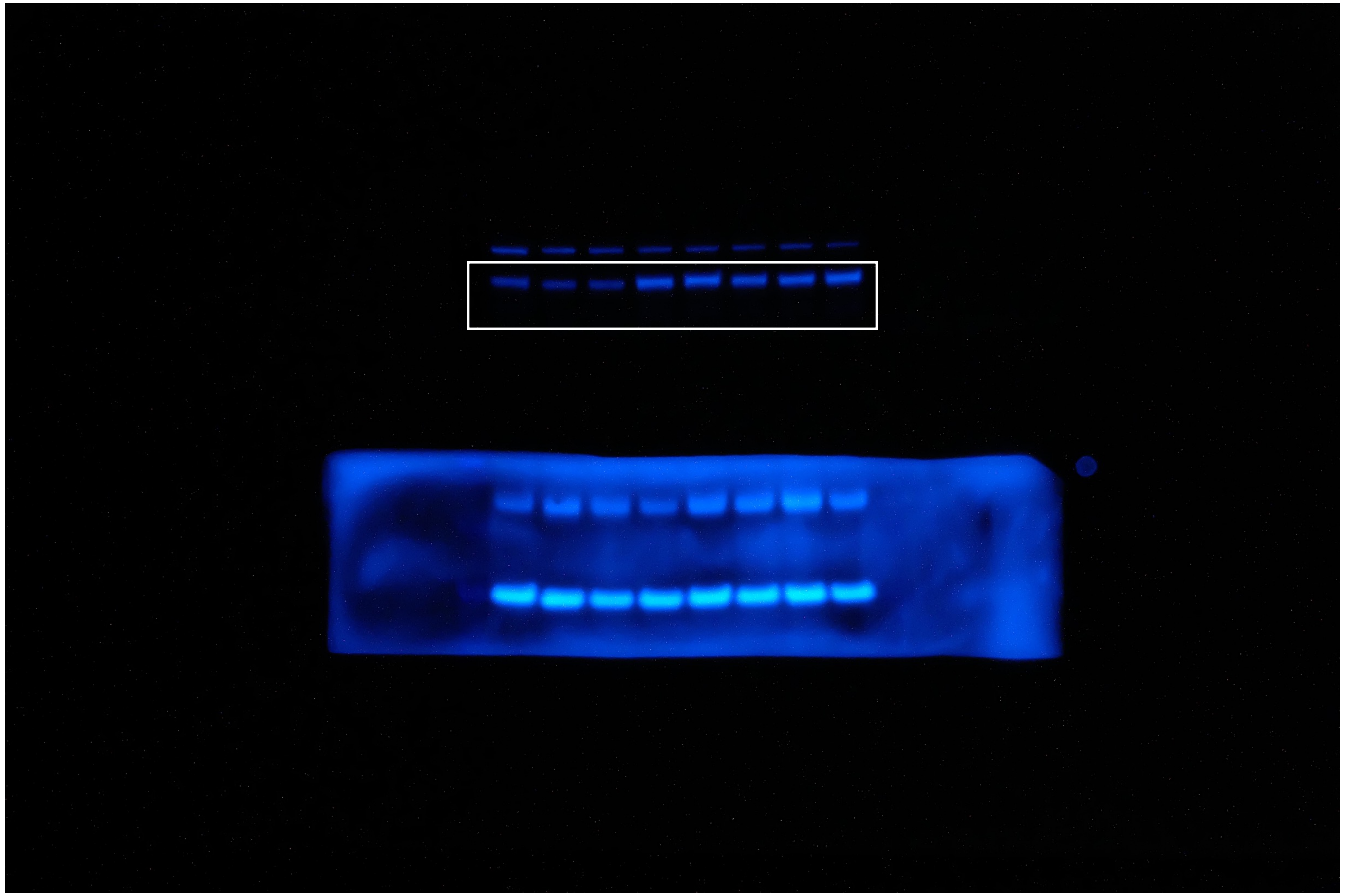

Full unedited gel for Figure 1C, Gapdh

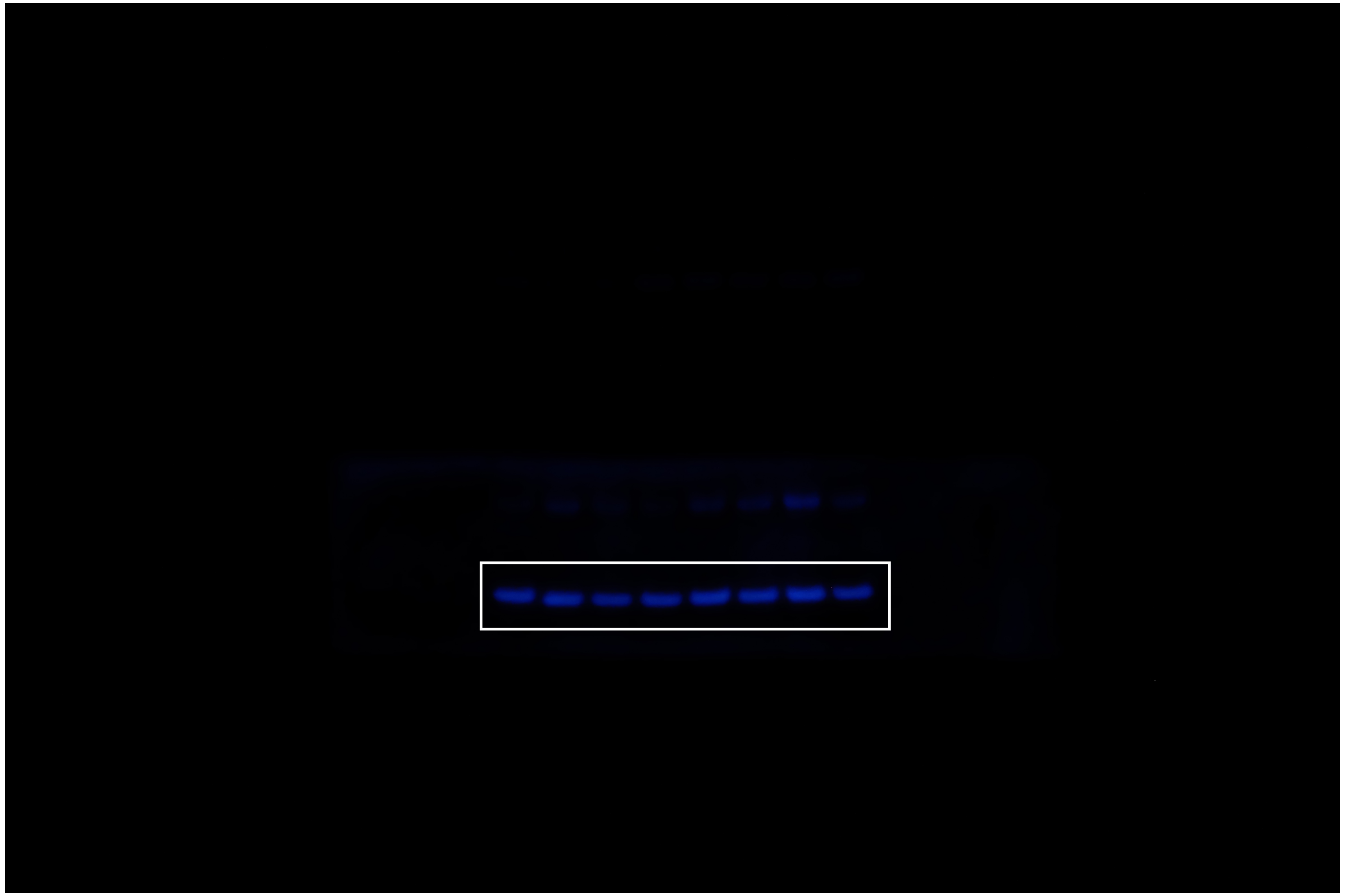

Full unedited gel for Figure 2G, Ezh2

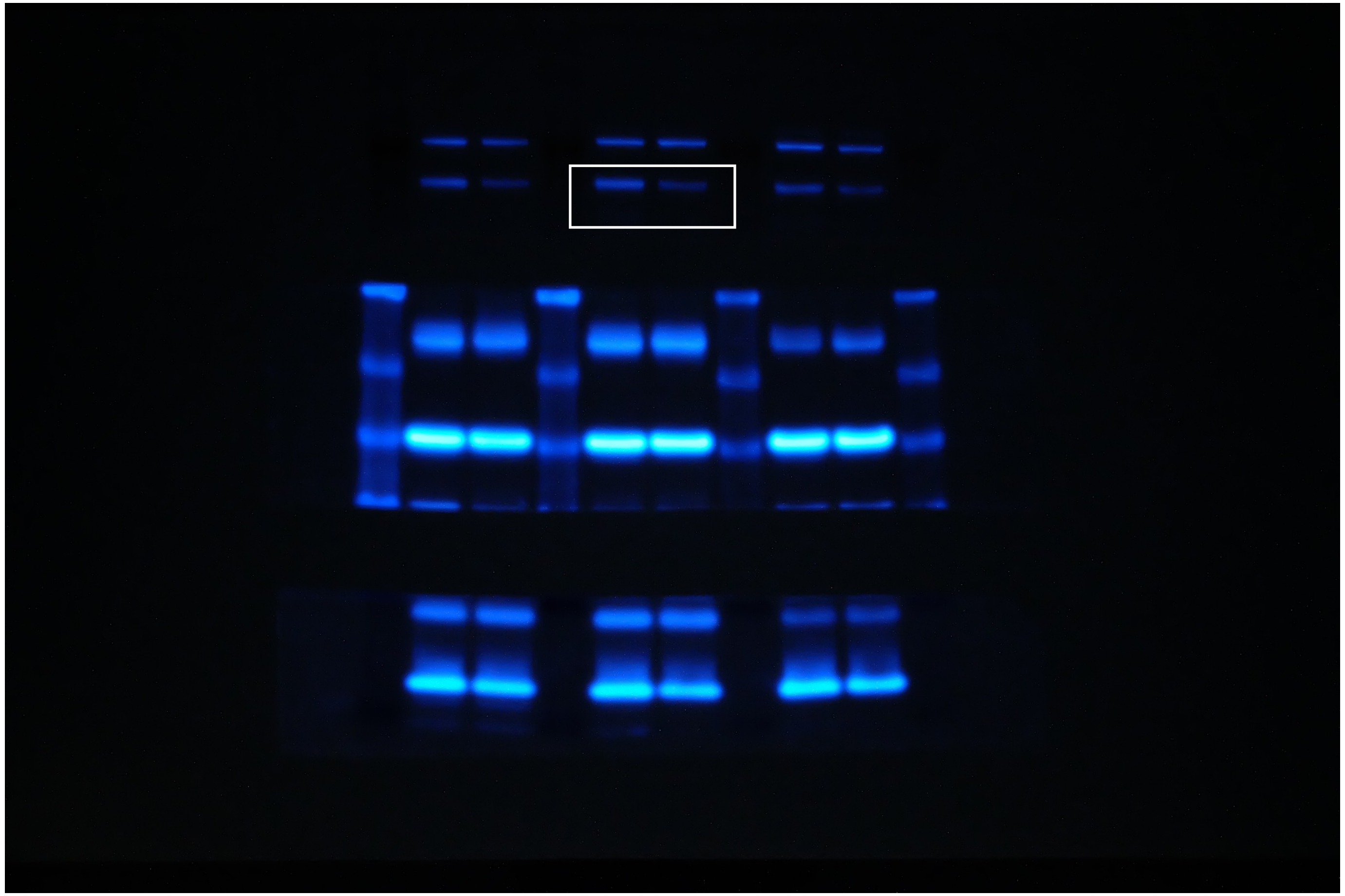

Full unedited gel for Figure 2G, β-actin

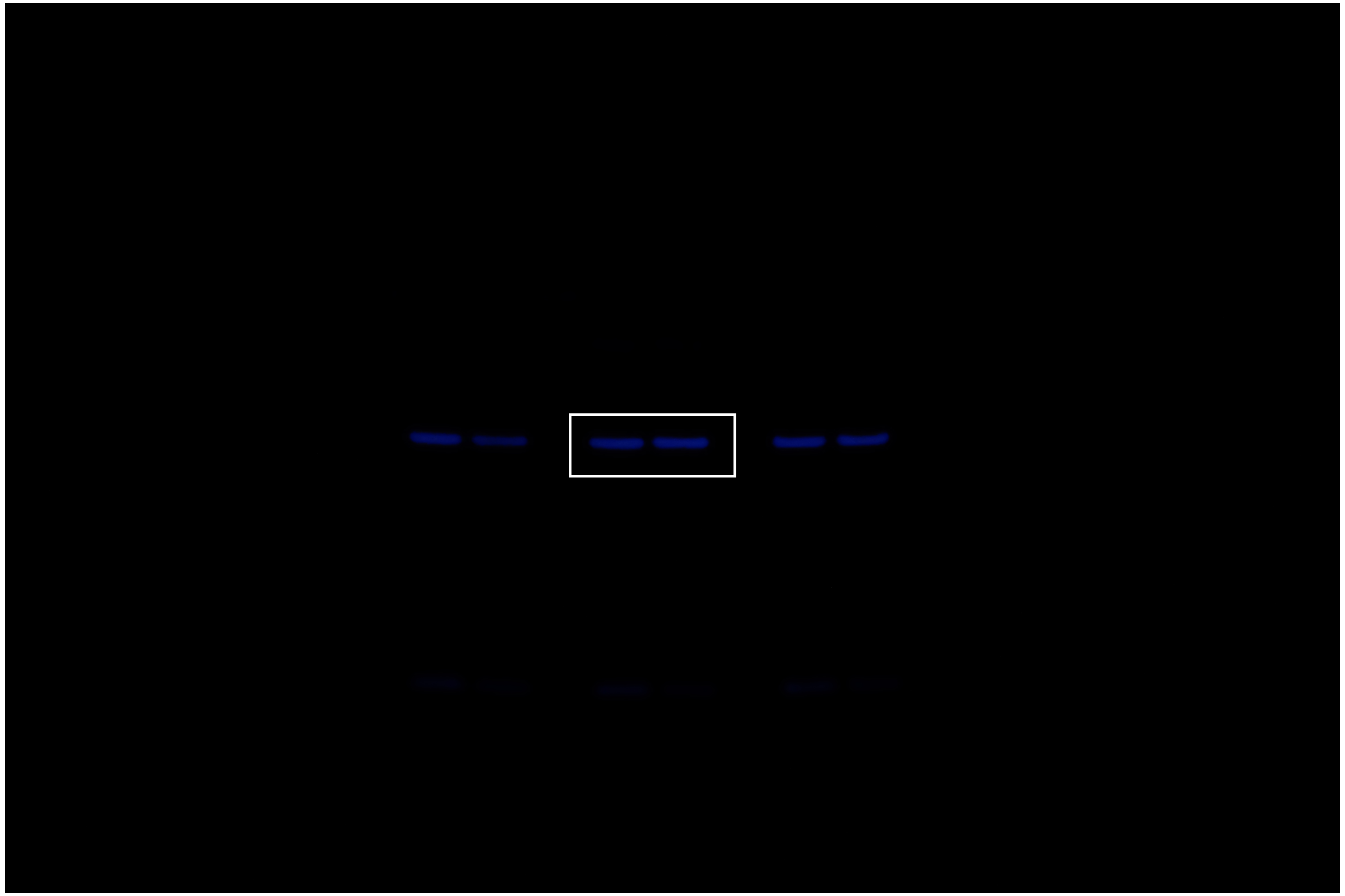

Full unedited gel for Figure 2G, H3K27me3

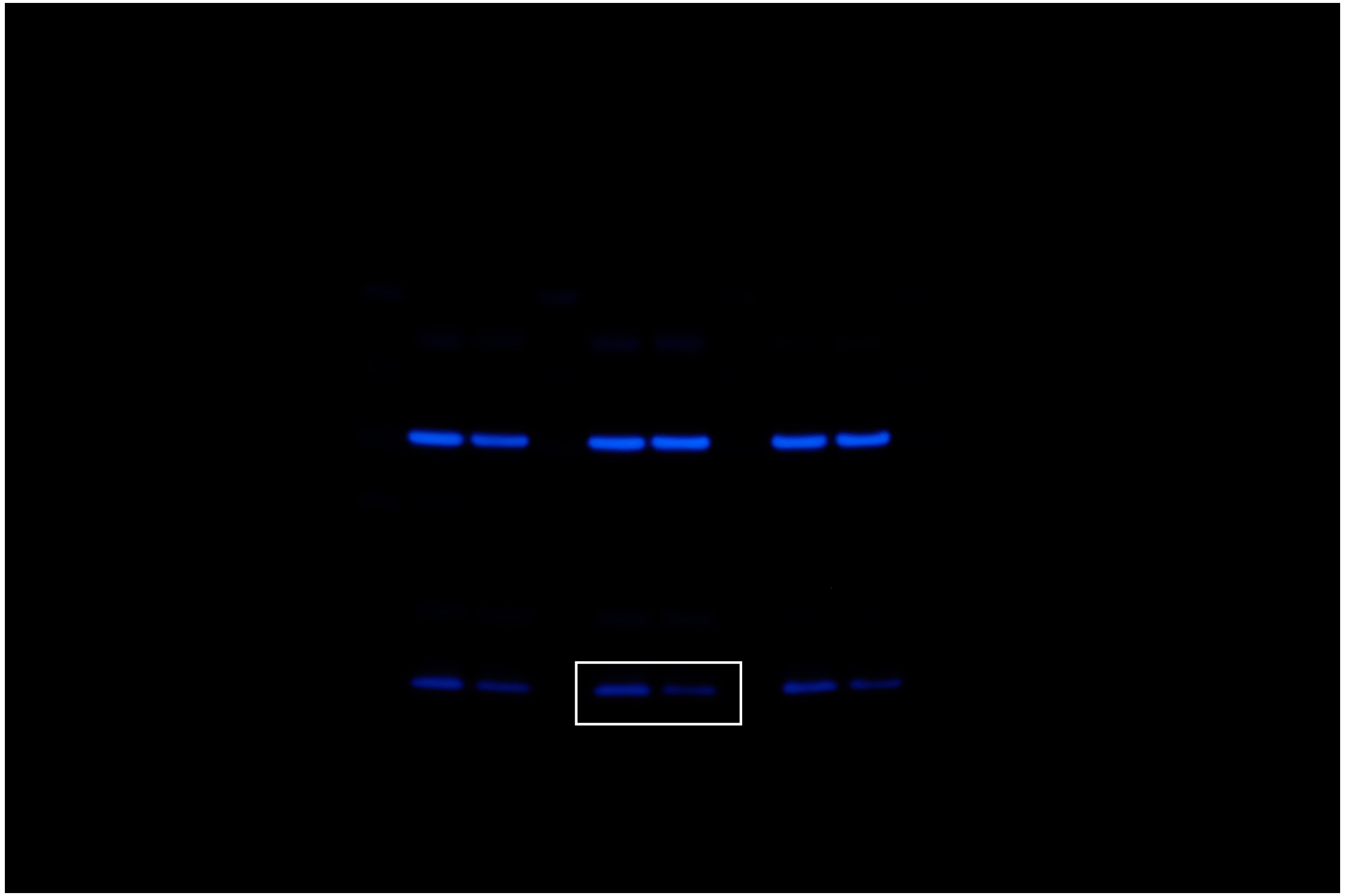

Full unedited gel for Figure 2G, H3

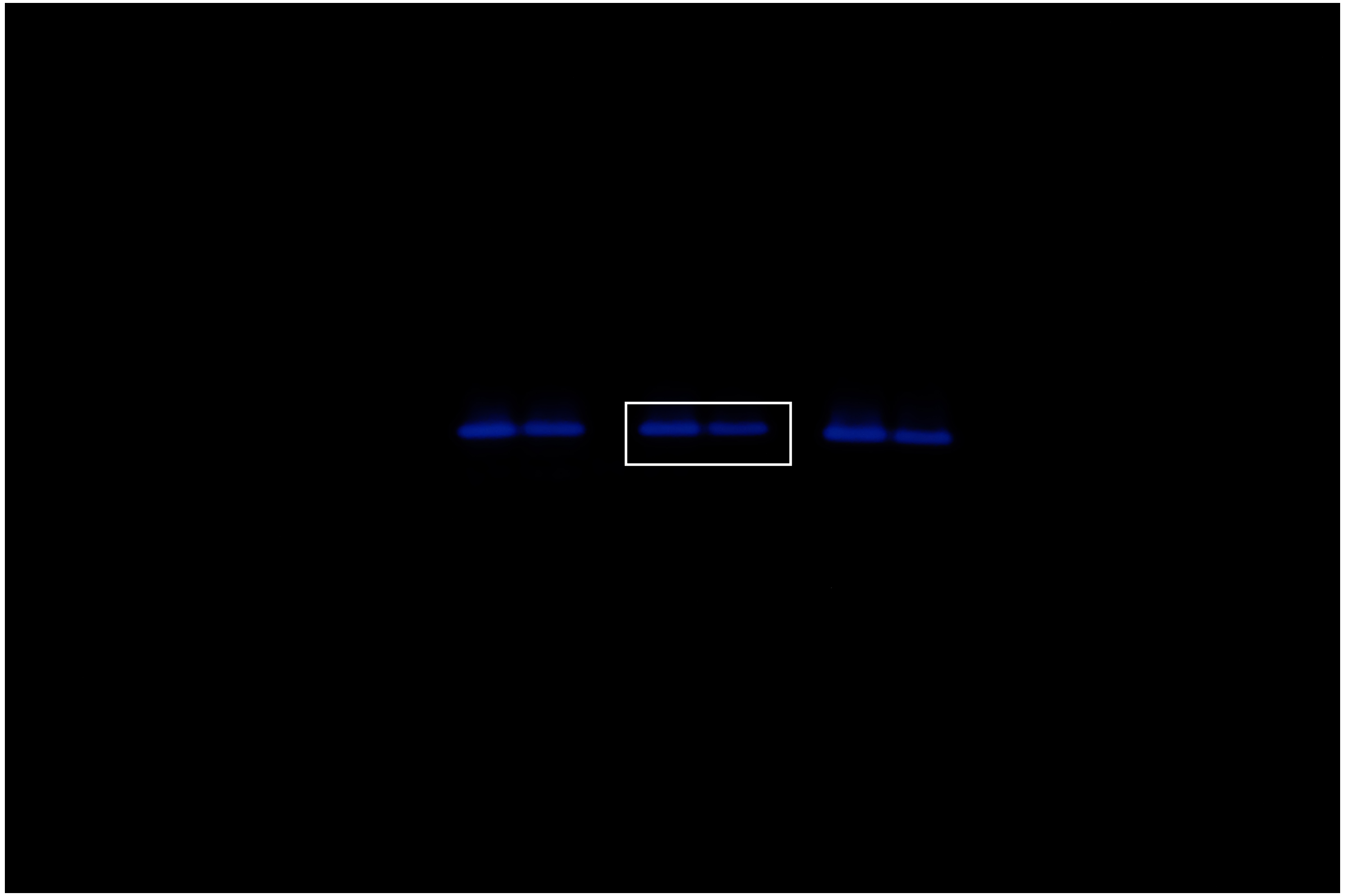

Full unedited gel for Supplemental Figure 1A, Ezh1

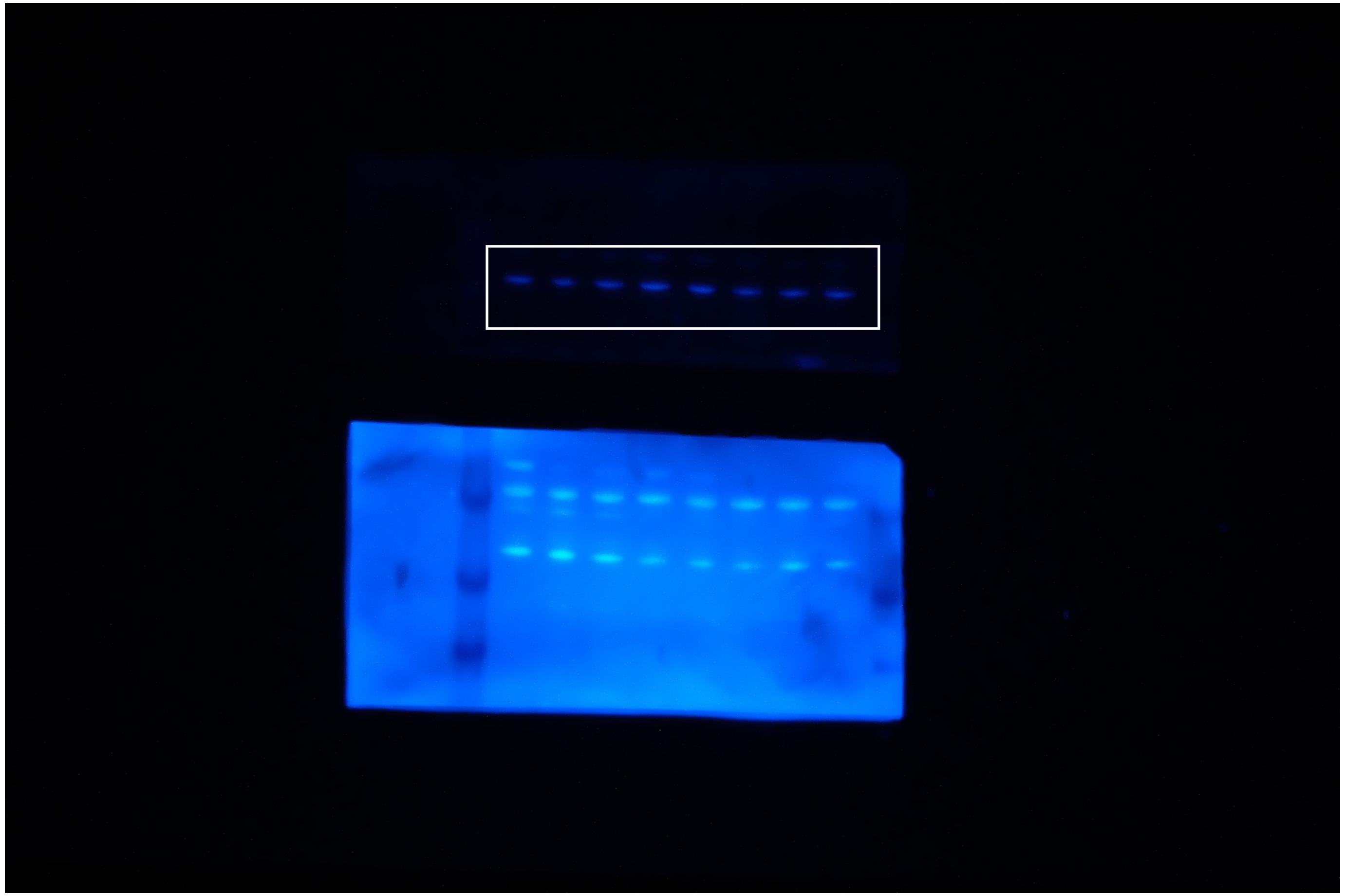

Full unedited gel for Supplemental Figure 1A, Suz12

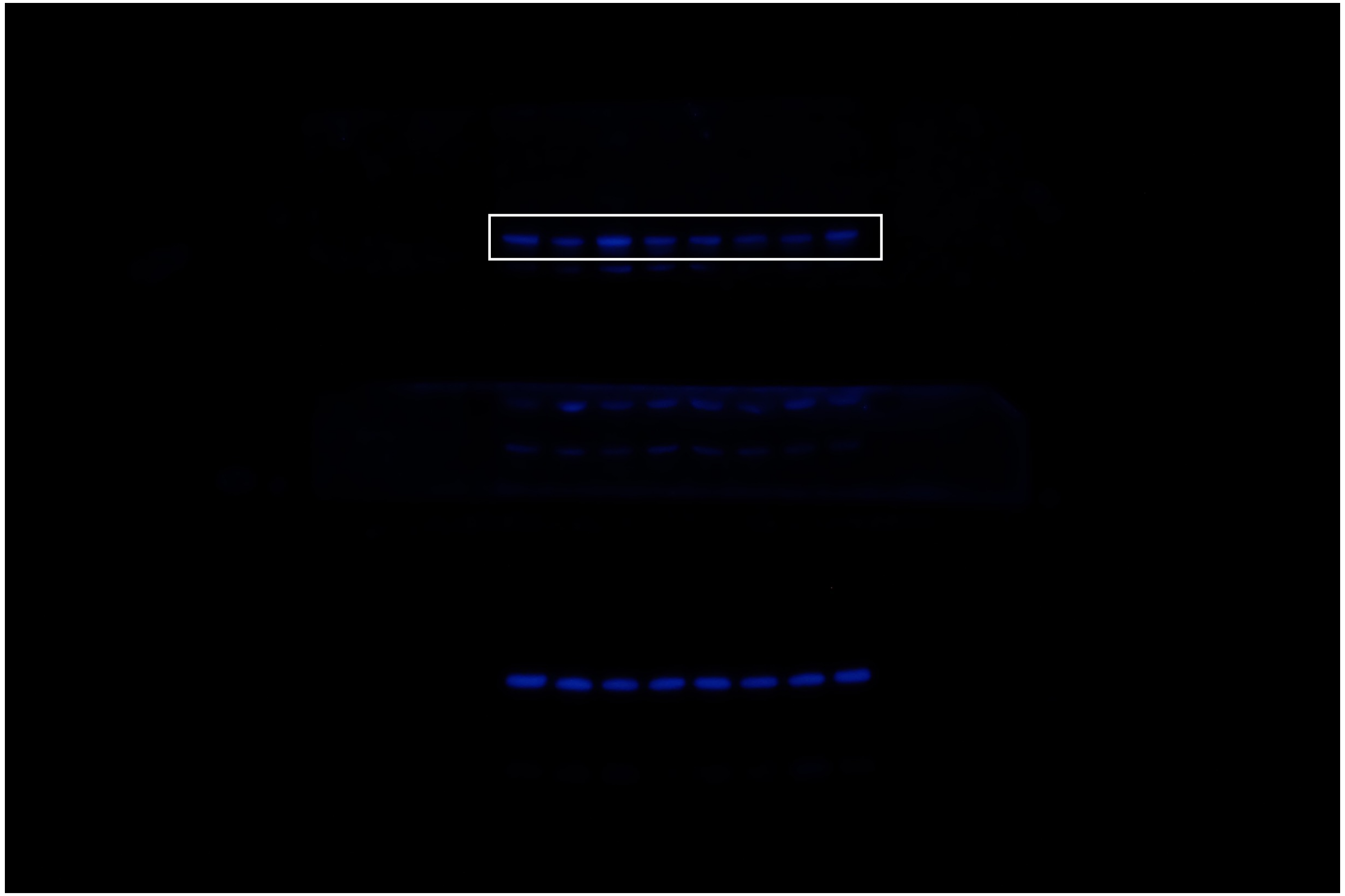

Full unedited gel for Supplemental Figure 1A, Eed

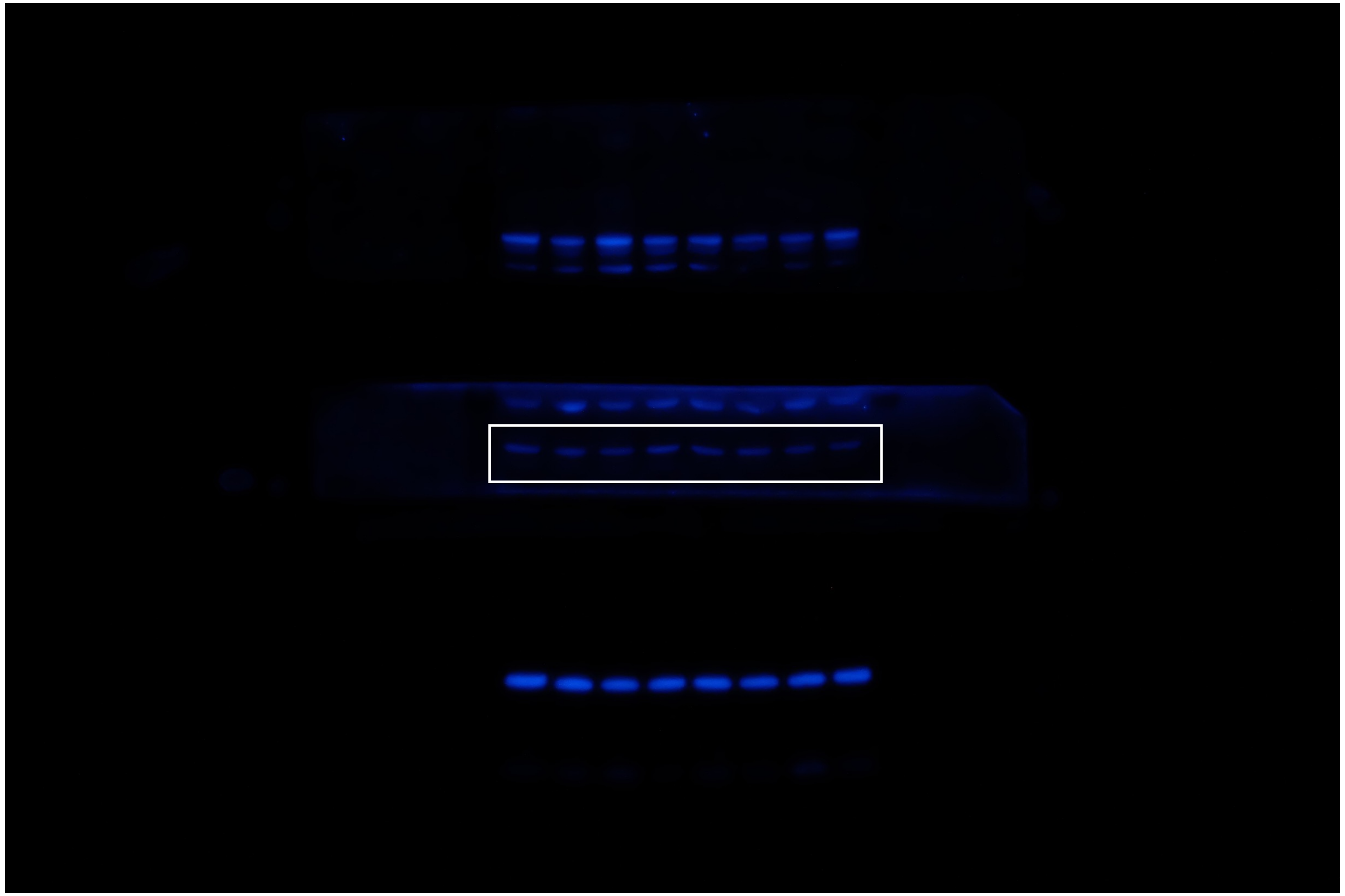

Full unedited gel for Supplemental Figure 1A, Rbap46/48

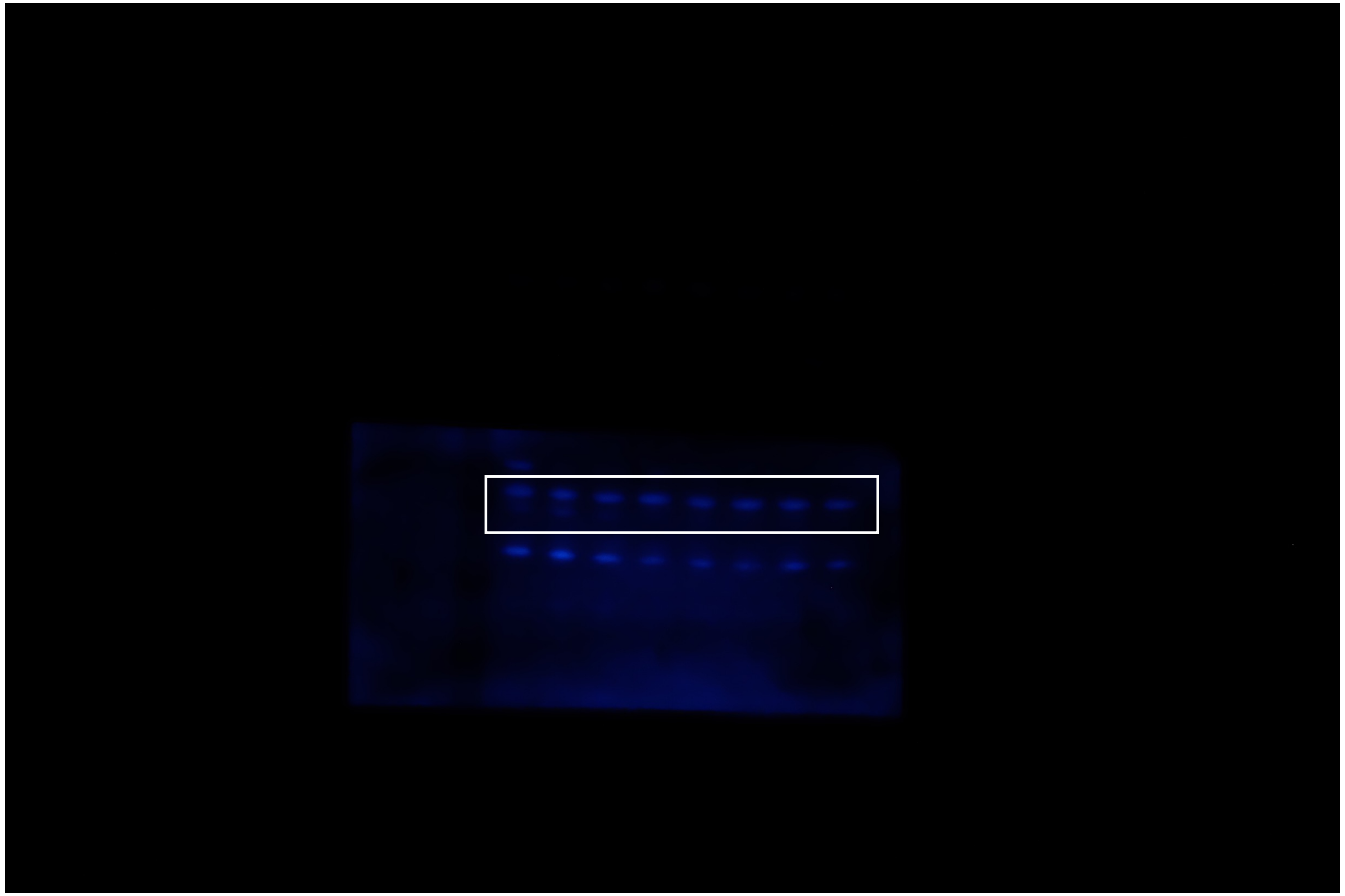

Full unedited gel for Supplemental Figure 1, Gapdh

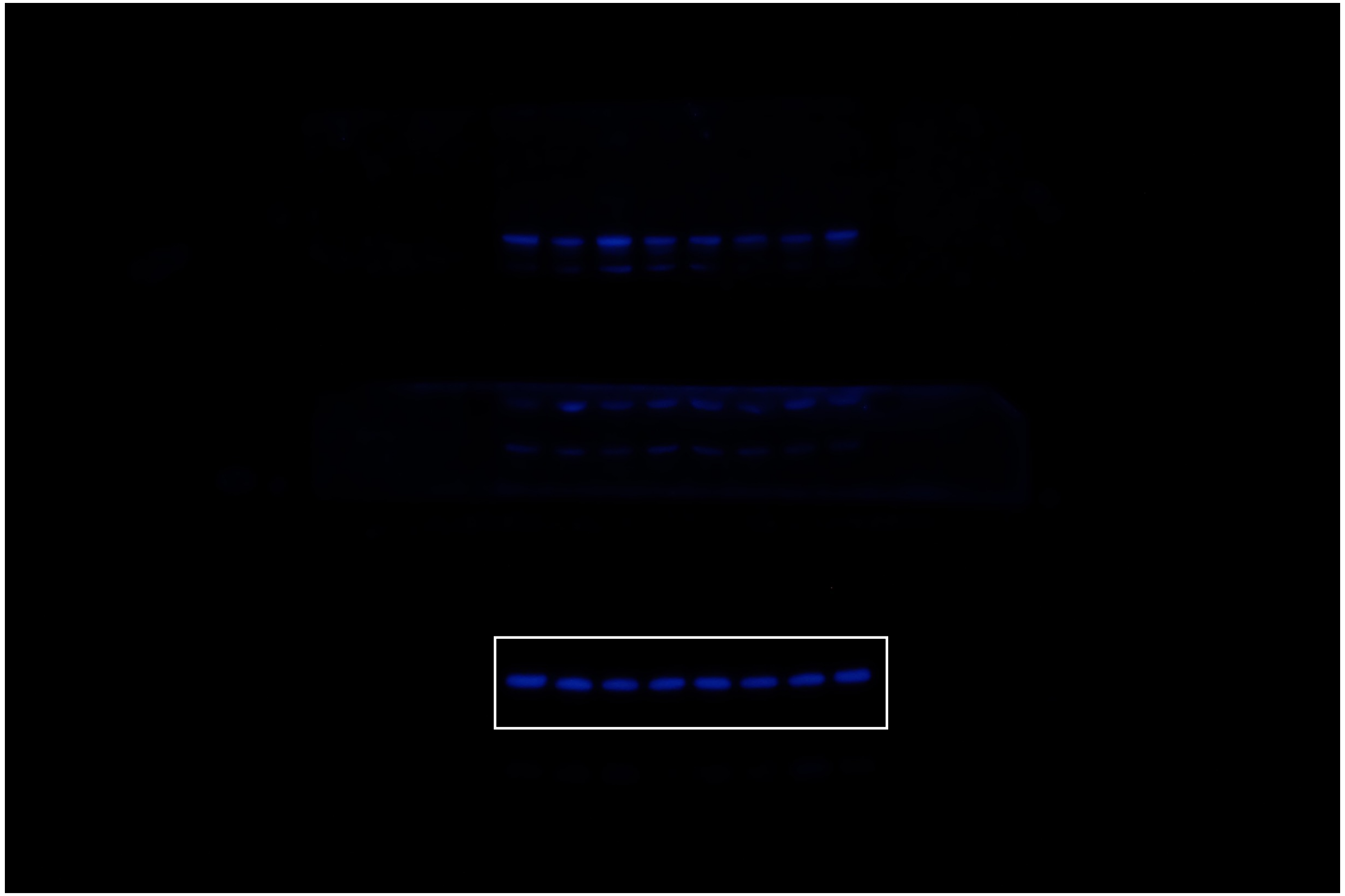

Full unedited gel for Supplemental Figure 1F, Ezh2

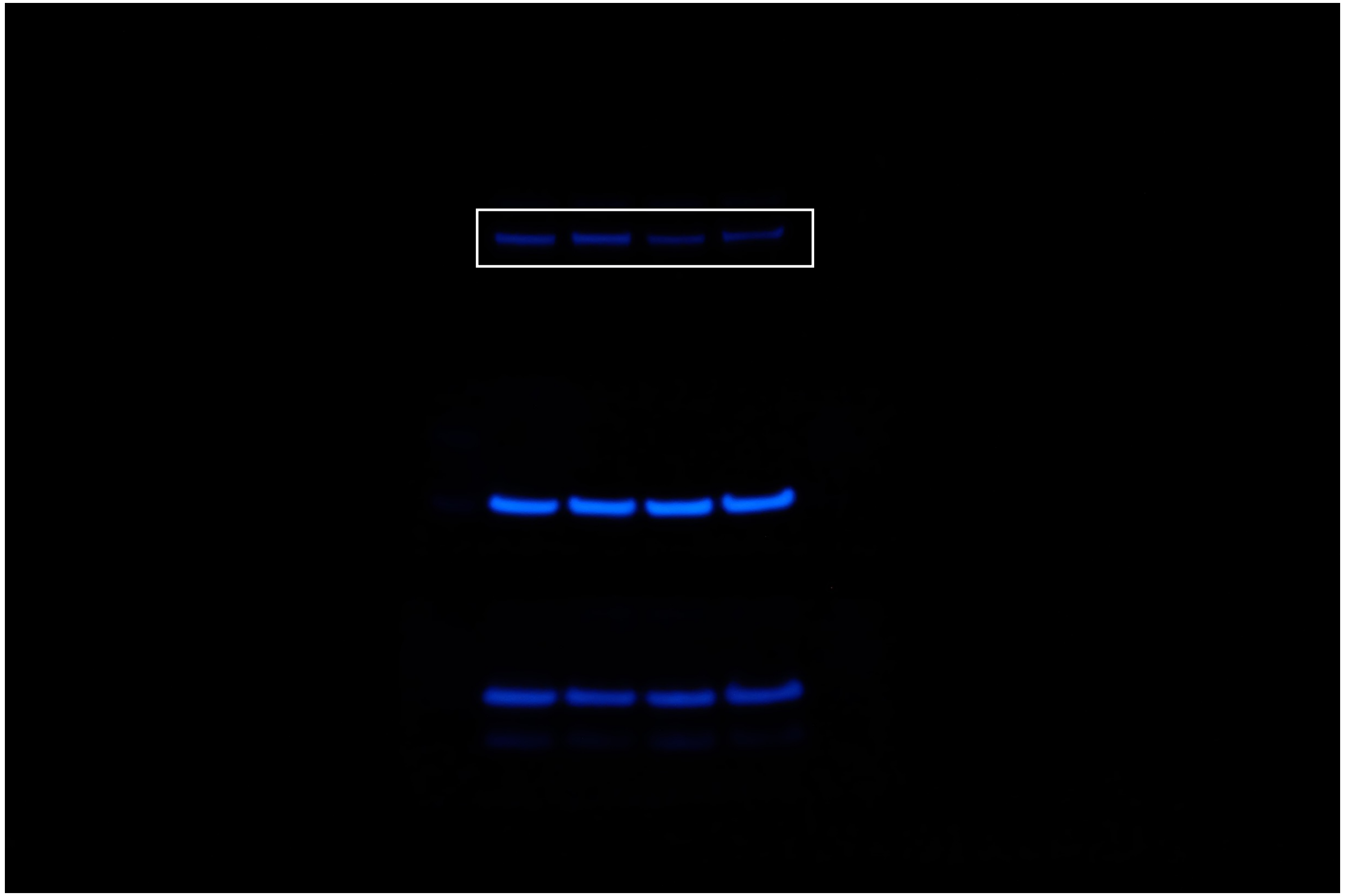

Full unedited gel for Supplemental Figure 1F, Gapdh

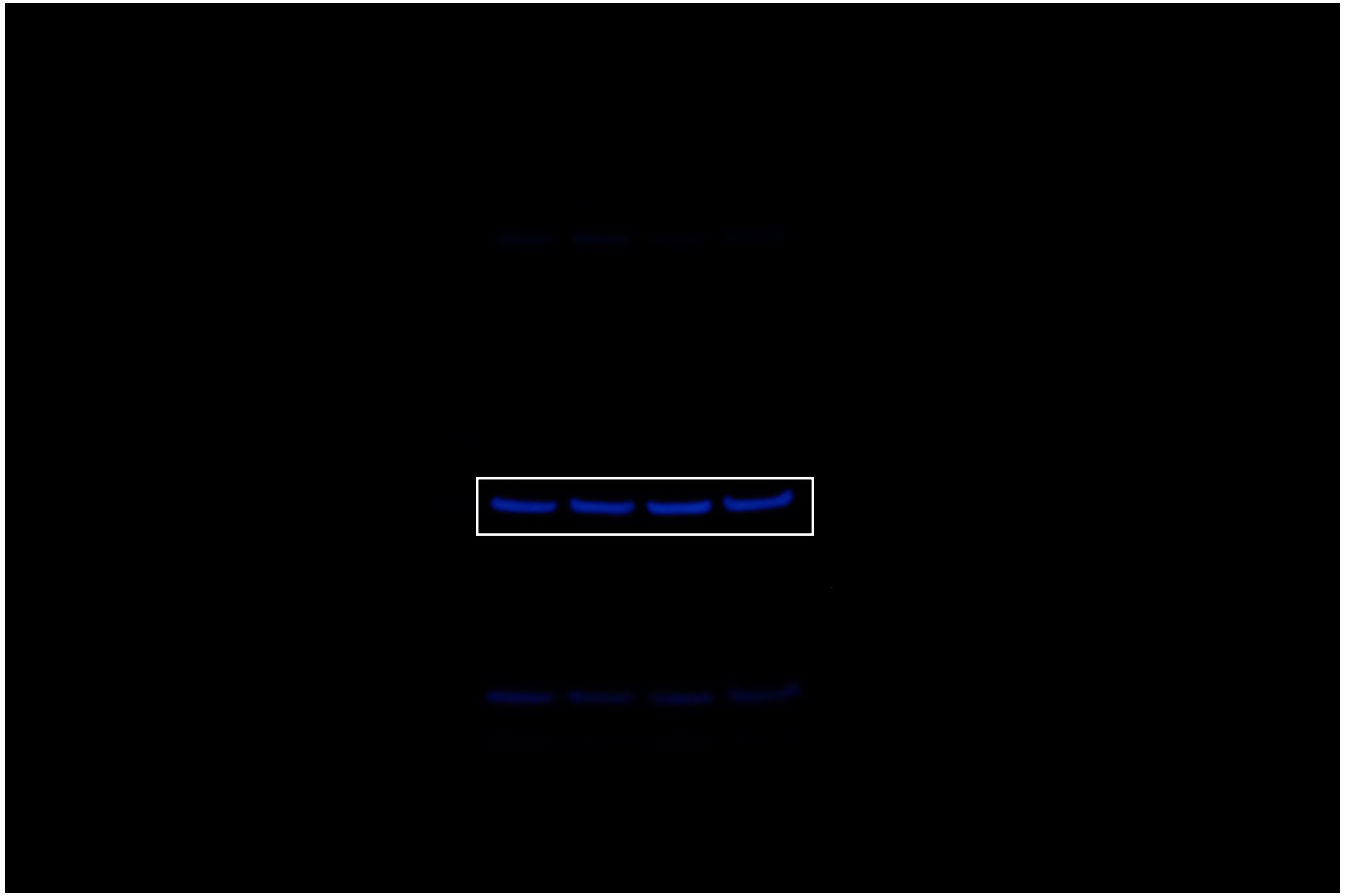

Full unedited gel for Supplemental Figure 1G, Ezh2

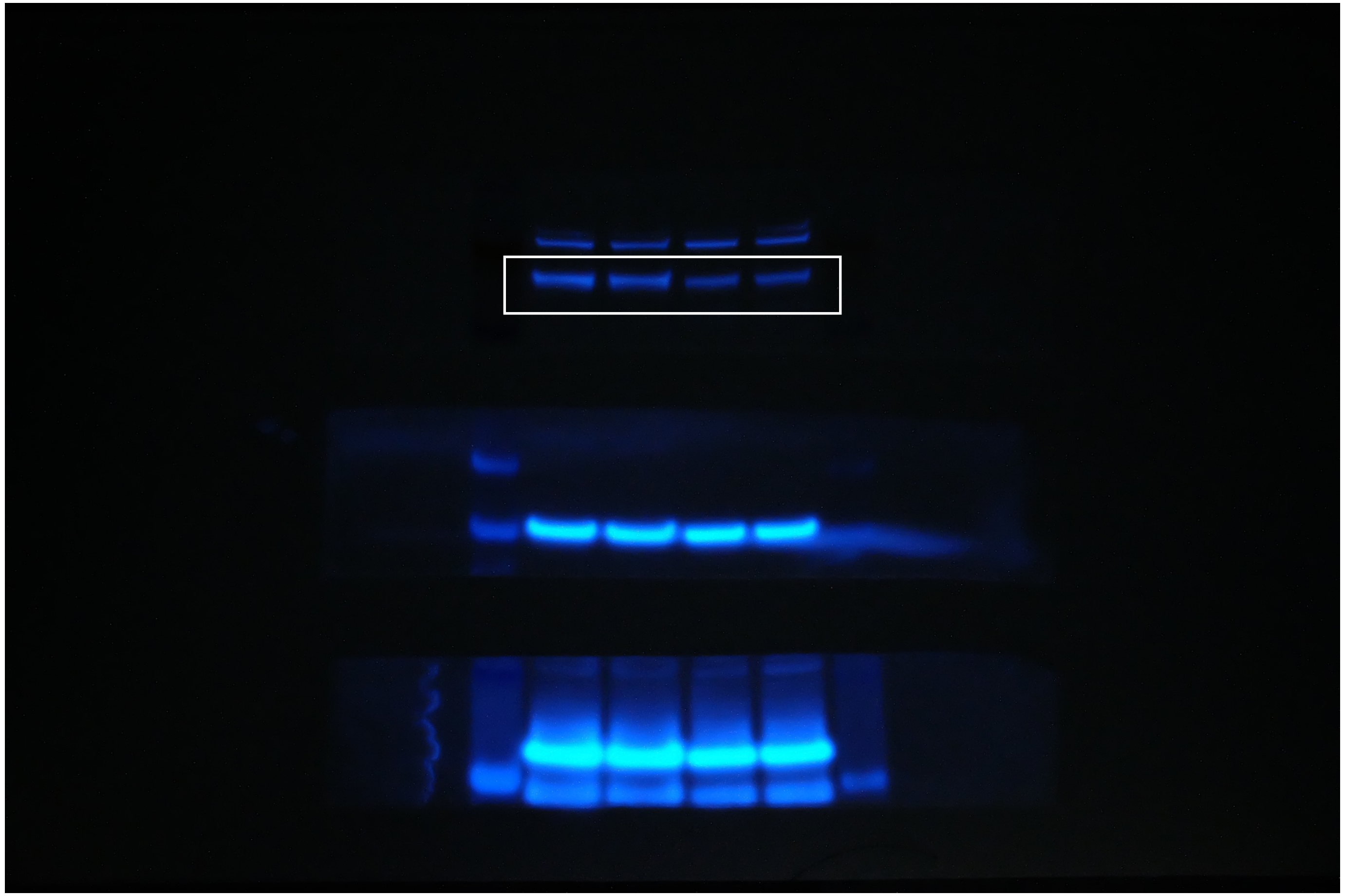

Full unedited gel for Supplemental Figure 1G, Gapdh

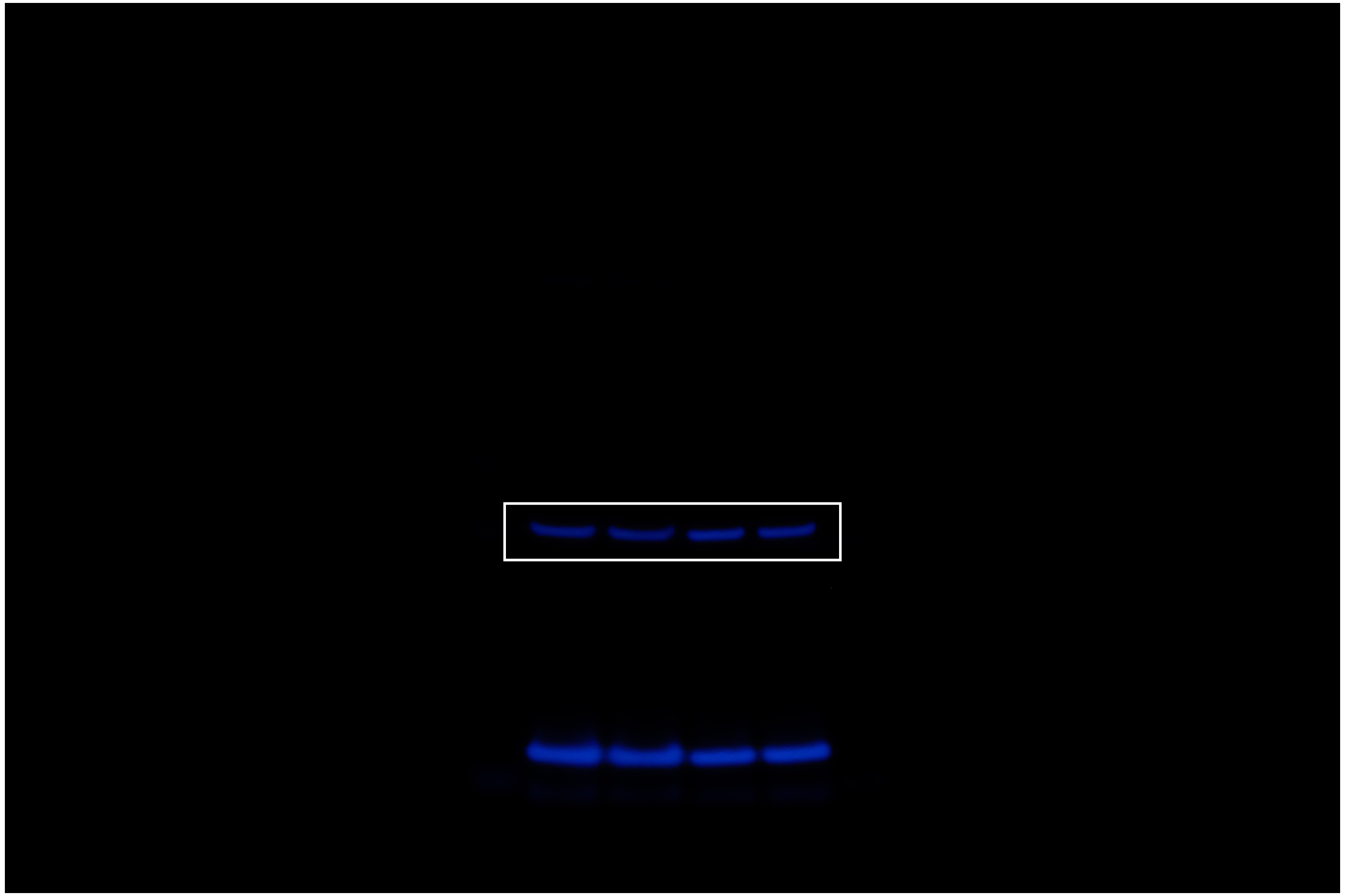

Full unedited gel for Supplemental Figure 3A, Ezh2

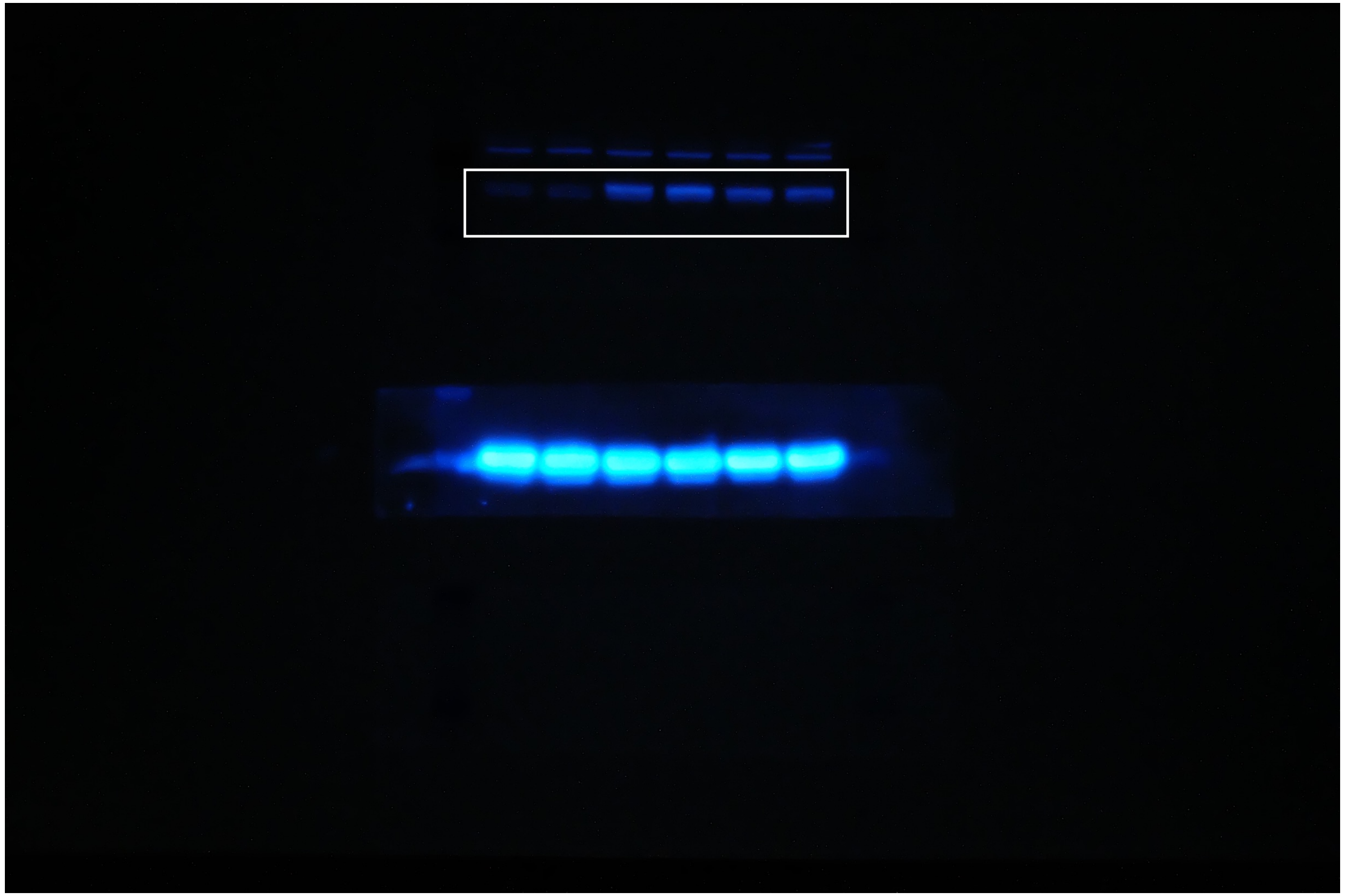

Full unedited gel for Supplemental Figure 3A, Gapdh

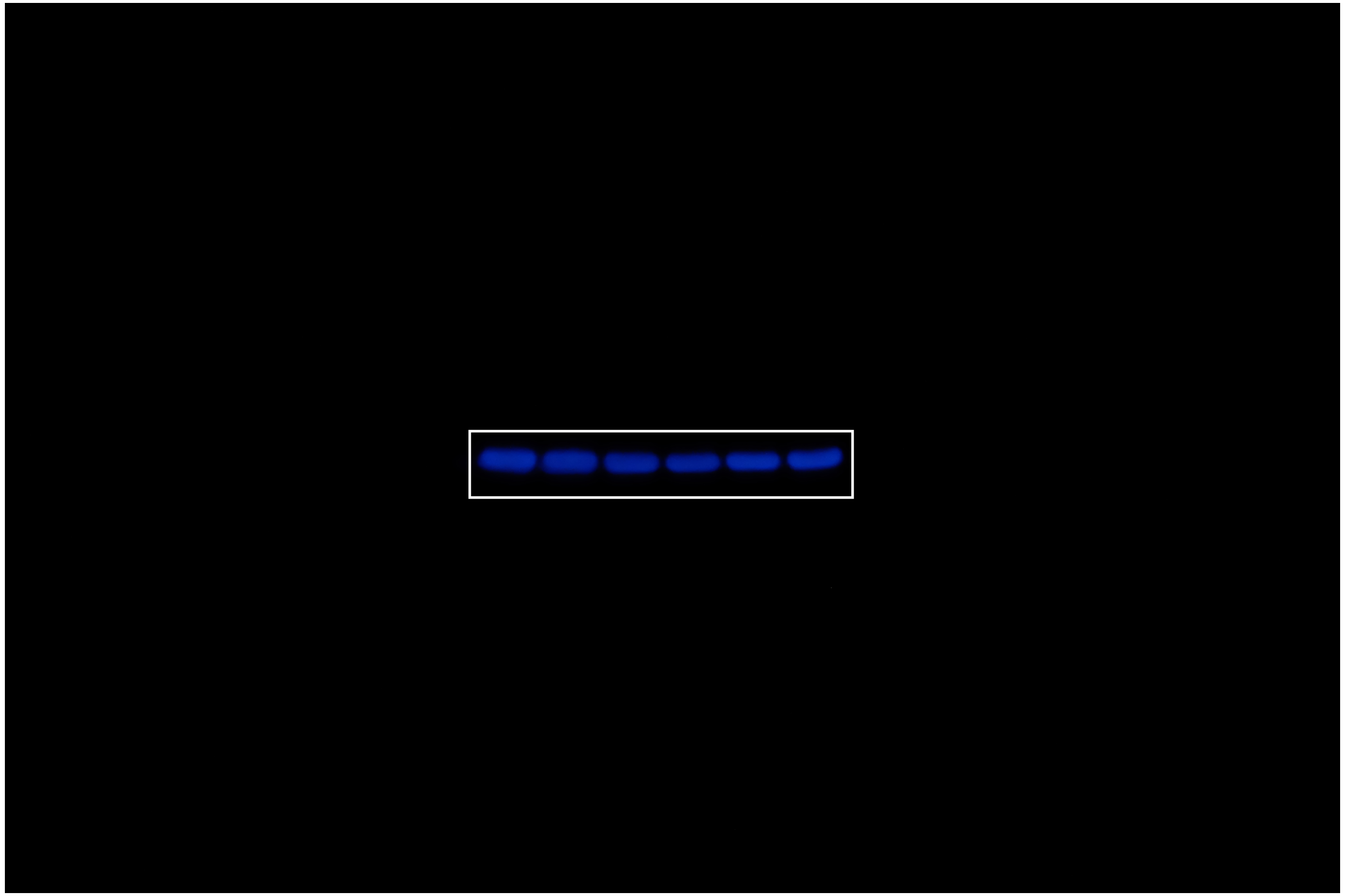

Full unedited gel for Supplemental Figure 3B, Ezh2

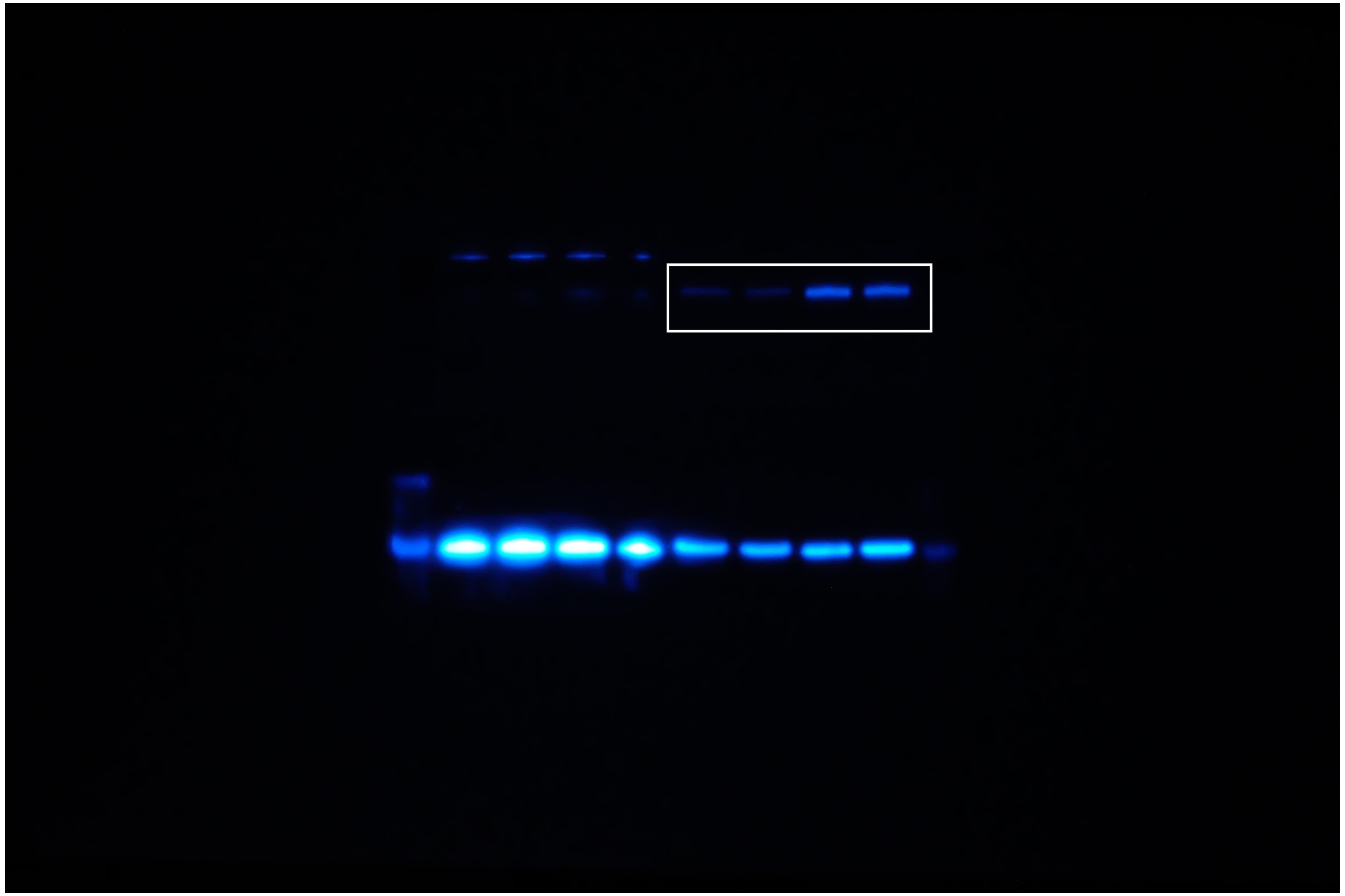

Full unedited gel for Supplemental Figure 3B, Gapdh

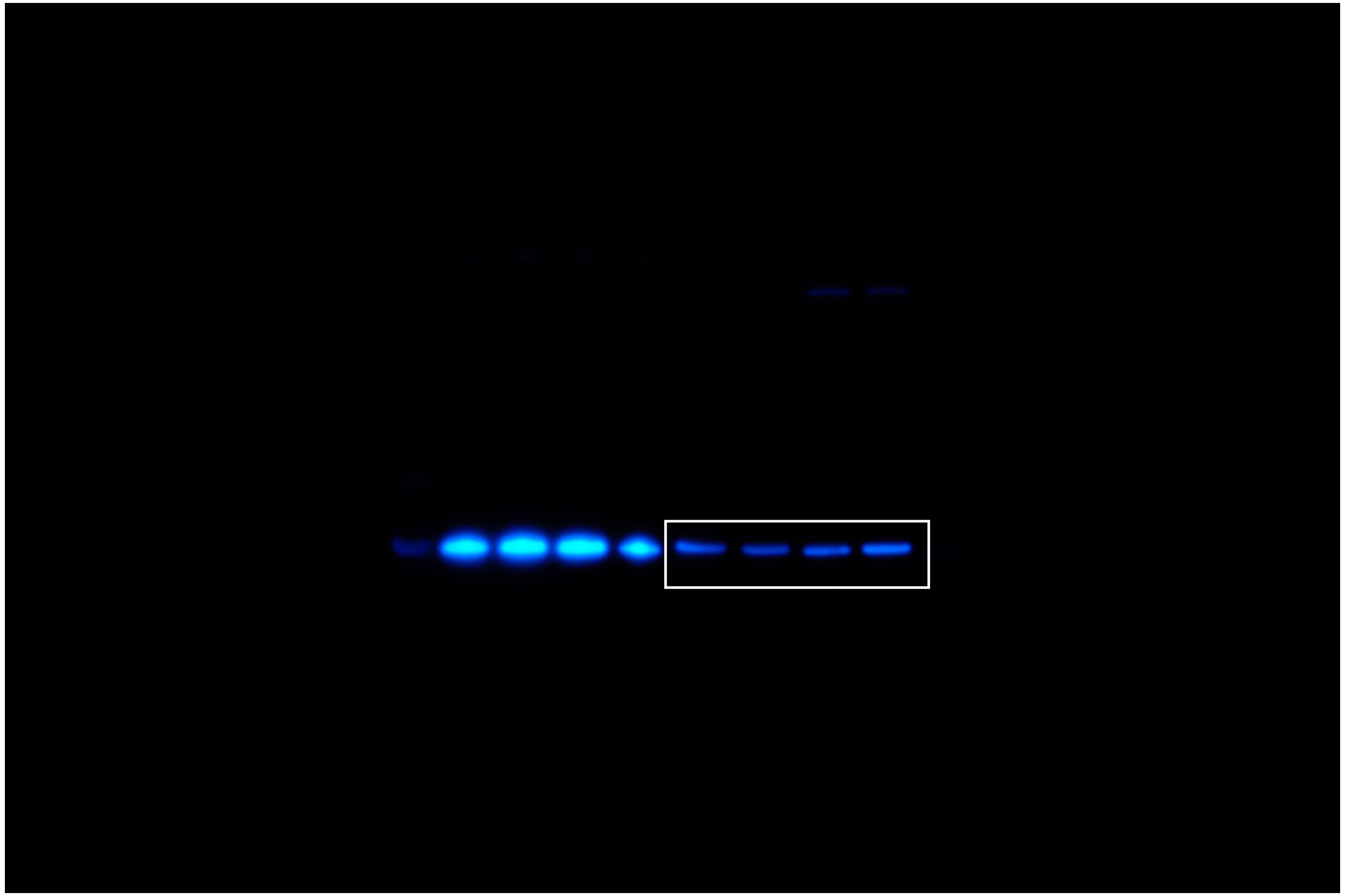

Supplement: Unedited blot and gel images [file jci-134-163145-s011.pdf]
